# Cellular Metabolic Signatures of Long COVID-19

**DOI:** 10.3390/idr18030050

**Published:** 2026-05-26

**Authors:** Sujata Srikanth, Diana Ivankovic, Lucia Gonzales, Delphine Dean, Luigi Boccuto

**Affiliations:** 1 Center for Innovative Medical Devices and Sensors (REDDI Lab), Clemson University, Clemson, SC 29634, USA; finou@clemson.edu; 2 School of Nursing, College of Behavioral, Social and Health Sciences, Clemson University, Clemson, SC 29634, USA; divanko@clemson.edu (D.I.); luciag@clemson.edu (L.G.); lboccut@clemson.edu (L.B.); 3 Department of Bioengineering, Clemson University, Clemson, SC 29634, USA

**Keywords:** long COVID-19, COVID-19, metabolism, SARS-CoV-2, metabolic changes, cellular metabolic signature

## Abstract

Background/Objectives: Long COVID-19 (LC-19), also known as Post-Acute COVID-19 Syndrome (PACS), is a chronic condition some people experience after an initial SARS-CoV-2 infection. The etiology of this complex, multifactorial disease remains largely unknown, although various theories have been propounded. This study aims to profile and compare the metabolic activity of cells of normal and LC-19 patients. Methods: A cohort of 20 individuals, 10 with LC-19 and 10 without LC-19, was selected based on their post-COVID-19 symptomatology. Saliva was tested for opportunistic viruses like Epstein–Barr virus (EBV) and Human Herpesvirus 6 (HHV-6). Lymphoblastoid cell lines derived from blood were analyzed using the Biolog Phenotype Mammalian Microarrays (PM-M1, PM-M6, and PM-M7) to assess metabolic activity across a wide array of growth substrates and effector molecules. Results: Unique metabolic profiles emerged across the controls and LC-19 groups. The SARS-CoV-2 infection causes an over two-fold enhanced utilization of glycolytic and anaerobic substrates and a reduced response to growth factors and effectors. The increased energy source utilization assessed in PM-M1 is unsustainable, and the LC-19 groups demonstrate this with a clear correlation with the number of LC-19 symptoms, demonstrating a trend consistent with metabolic reprogramming. The infection also results in a reduced response to growth factors and effectors, assessed in PM-M6 and PM-M7, with the level of reduction commensurate with the symptom burden. Conclusions: The data from the patient groups were analyzed and compared to construct a metabolic profile unique to individuals who developed LC-19, which could, in the future, be used for diagnosis and to identify targets for therapeutic intervention. Our study identified an LC-19-specific metabolic profile indicative of adaptive responses to stress, cellular dysfunction, and prolonged inflammation, leading to the reprogramming of bioenergetic pathways.

## 1. Introduction

Severe acute respiratory syndrome coronavirus 2 (SARS-CoV-2), the cause of COVID-19, emerged in 2019 and rapidly spread worldwide [1]. Infection ranges from asymptomatic to symptoms such as fever, cough, shortness of breath, fatigue, and loss of taste or smell [2,3]. During the early phase of the pandemic, more than six million deaths were reported globally, prompting widespread public health interventions to mitigate both human and socioeconomic consequences [4]. Most individuals recover from acute infection within 1 to 2 weeks and return to their pre-infection functional status [5]. However, a subset of patients testing negative after the acute phase experience persistent or newly emerging symptoms, including fatigue, dyspnea, chest pain, low-grade fever, headaches, cognitive impairment, anxiety or depression, and gastrointestinal disturbances [6,7].

This condition, commonly referred to as Long COVID (LC-19), has also been described as long-haul COVID or post-acute coronavirus syndrome. It is broadly defined as signs and symptoms that persist or develop after an acute SARS-CoV-2 infection. According to the World Health Organization, LC-19 is characterized by symptoms that begin during or after the initial illness and persist for at least two to three months without an alternative explanation [8]. Although the mechanisms underlying this condition remain incompletely understood, it affects individuals across all age groups and levels of initial disease severity. Patients report a wide range of respiratory, neurological, cardiovascular, gastrointestinal, and psychosocial symptoms that vary in onset, intensity, and duration [9,10].

Despite increasing research into pulmonary, cardiac, and neurological complications, the long-term morbidity of LC-19 remains incompletely defined [11]. Longitudinal studies indicate a slow recovery, with a relatively small proportion of patients achieving full recovery even after extended follow-up periods [12]. Severe acute infection or prolonged viral shedding increases the risk of developing persistent symptoms [13]. Mechanisms proposed to explain the diverse manifestations of LC-19 include viral persistence [14], autoimmune responses [15], chronic inflammation [16], mitochondrial dysfunction [17,18], dysregulation of cytokine signaling [19], alterations in the gut microbiome [20], and reactivation of latent viruses such as the Epstein–Barr virus [21]. However, no single unifying mechanism has yet been confirmed.

Most current research has focused on older adults, individuals with underlying health conditions, or pediatric populations, whereas young adults remain relatively understudied [3,9,22]. Emerging evidence suggests that a significant proportion of individuals aged 17–24 continue to experience symptoms weeks after infection, including fatigue, cognitive difficulties (“brain fog”), reduced exercise tolerance [23] and stigmatization due to physiological changes, even among individuals without prior mental health conditions [24,25]. The widespread functional impairment associated with LC-19 has implications at multiple levels, including reduced workforce participation, increased healthcare utilization, and diminished quality of life [26,27,28,29]. The health and socioeconomic effects can be explained by the Biopsychosocial (BPS) model of disease, which integrates biological, psychological, and social factors [30]. Within this context, the present study examines cellular metabolic alterations in affected individuals to better understand the biological mechanisms underlying the disease and to inform targeted interventions.

## 2. Materials and Methods

### 2.1. Research Design

This study employed a mixed-methods approach. A cross-sectional online survey of Clemson University residents was conducted to collect qualitative data on post-COVID-19 (post-C-19) symptomatology. The survey was then used to recruit volunteers for the quantitative portion of the study, namely, the metabolic profile assessment.

### 2.2. Survey

The survey was conducted among students and employees (although most respondents were students) on the Clemson University campus (Clemson, SC, USA). We obtained ethics approval from the Clemson University Institutional Review Board (IRB2023-0494). Participants were informed that their personal information and responses would be confidential. The survey was active from 9 May 2023 to 23 October 2023.

We designed the first survey using a Qualtrics^®^ template with questions about their post-C-19 health status. In addition to questions about the timing of C-19 infection and vaccine status, the questionnaire included questions about respondents’ post-C-19 symptoms. The extent to which symptoms affected their quality of life was assessed on a Likert scale. Respondents who replied to the Qualtrics^®^ survey were eligible to be part of a drawing for ten gift cards of $50.00 each.

The second survey, also conducted using a Qualtrics template, was then administered to those students in the first survey who indicated a willingness to participate in future surveys. This survey was more detailed, with questions about post-C-19 symptoms, pre-C-19 health status, and family medical history. The survey was active from 12 February 2024 to 26 March 2024. Respondents who completed the second survey were again eligible to enter a drawing for ten gift cards of $50.00 each.

### 2.3. Study Participant Recruitment

Participants were recruited based on responses to the second survey. Individuals with a clinical diagnosis of Long COVID (LC-19) or persistent post–COVID-19 symptomatology not attributable to alternative medical conditions were eligible for inclusion in the LC-19 group. An equal number of individuals who had fully recovered from COVID-19 and reported no persistent post-COVID-19 symptoms were selected as controls. Participants in both groups provided a peripheral blood sample and two saliva samples for analysis. The study protocol was reviewed and approved by the Clemson University Institutional Review Board (IRB2024-0198). All volunteers provided informed consent, and the study samples were de-identified. The participants’ age range was 21 to 29 years when the blood and two saliva samples were obtained. Table 1 provides details on the study participants.

### 2.4. SARS-CoV-2 Diagnostic Assay

RT-qPCR was used to assess SARS-CoV-2 infection status (and confirm that all were negative) among the study volunteers. One saliva sample from each participant was tested for C-19 status, and the other was frozen at −80 °C for further analysis. The saliva Nucleic Acid Amplification Test (NAAT), a quantitative reverse transcriptase-PCR (RT-qPCR) diagnostic test for C-19, used was the TigerSaliva multiplex RT-qPCR protocol, a modified version of the Emergency Use Authorization (EUA)-approved SalivaDirect protocol [31,32]. Briefly, 1 mL of saliva is collected from patients in standard 50 mL conical tubes. The saliva was heated to 95 °C for 30 min, after which 2 μL was loaded into test plates containing enzyme mix, primers, and probes. The assay measures the nucleocapsid phosphoprotein (N1) gene sequence of SARS-CoV-2 and the human RPP30 (Hs_RPP30) gene, used as a control for human DNA [33,34]. The Hs_RPP30 control contains a portion of the RPP30 gene (Ribonuclease P subunit p30), a conserved, housekeeping gene that encodes a protein component of the ribonuclease P (RNase P) complex. The RPP30 protein plays a well-established housekeeping role, is essential for basic cellular function, and is used as a stable internal reference in Nucleic Acid Amplification Tests (NAAT).

Standard curve analysis indicated that a Cycle threshold (Ct) of 33 for the RT-qPCR test corresponded to 1 viral copy per microliter (cpu) of saliva. Therefore, this value was used as the cutoff for SARS-CoV-2 RNA positivity. Samples were run in duplicate, and the average Ct value from both replicates was used for this analysis. Details of the probes, primers, and the amplification step cycle used are available in (Supplementary Materials Tables S1 and S2).

### 2.5. qPCR for EBV, HHV-6

qPCR was used to detect EBV and HHV-6 [35,36]. The test was performed using the CFX96 Touch Real-Time PCR Detection System (Bio-Rad Laboratories, Hercules, CA, USA). The assays were performed using the Luna Universal One-Step RT-qPCR Kit (New England Biolabs, Ipswich, MA, USA), with 4 μL of template (saliva) in a final reaction volume of 20 μL, containing a gene-specific TaqMan probe–primer set. Details of the probes, primers and the amplification step cycle used are available in (Supplementary Materials Tables S3 and S4).

### 2.6. Generation of Lymphoblastoid Cell Lines (LCLs)

Lymphoblastoid cell lines (LCLs) were generated for all participants by transducing patient peripheral blood mononuclear cells (PBMCs) with EBV using a well-established protocol [37]. Peripheral blood samples were obtained by venipuncture and collected in tubes containing anticoagulant citrate dextrose. The tubes were stored at room temperature and processed within 24 h of collection. PBMCs were prepared using Ficoll (Ficoll-Paque Plus, Fisher, Hampton, NH, USA, Lot #10255486). The PBMCs were collected (with no obvious red blood cell contamination), washed with PBS, and the cells re-suspended in 1 mL of Fetal Bovine Serum (Optima, Atlanta Biologicals, Flowery Branch, GA, USA, Lot # E17075) and 3 mL of RPMI medium (RPMI-1640, Corning Life Sciences, Corning, NY, USA). A 1 mL volume of EBV (VT-1492, ATCC, Manassas, VA, USA) was added, and cells were transferred to a culture dish containing phytohemagglutinin (Gibco-BRL, Fisher Scientific, Waltham, MA, USA). The culture dish was incubated at 37 °C at 10% CO_2_ for 5 to 7 days to ensure complete transformation. After the transformation was achieved, the cells were expanded in culture medium composed of RPMI medium modified with 15% FBS, 1% L-glutamine (Corning), and 1% antibiotic/antimycotic (Corning). The supernatant cell-free spent medium was removed every fourth day, and fresh medium was added. We harvested the LCLs and froze aliquots in liquid nitrogen once the cells were actively proliferating and expanding in culture, had a viability of over 50%, and were in sufficient numbers.

### 2.7. Cell Culturing and Passaging

Cells were thawed from liquid nitrogen and cultured in RPMI-1640 with 15% fetal bovine serum (FBS) and antibiotics in T-75 cm^2^ untreated, sterile cell culture flasks in an incubator at 37 °C with 5% flowing CO_2_. Before the experiments, LCLs were harvested and centrifuged at 1000 rpm for 30 min, and the resulting pellet was resuspended in Biolog media. The Trypan Blue Exclusion Assay was used to measure cell viability, and a 50% viability cutoff was established for the assays.

### 2.8. Biolog Phenotype Mammalian Microarrays

Metabolic profiling was assessed in LCLs using the Phenotype Mammalian MicroArray (PM-M) technology, developed by Biolog (Biolog, Hayward, CA, USA), to measure cellular production of reduced nicotinamide adenine dinucleotide (NADH) in the presence of different compounds. The production of NADH per well is monitored using a colorimetric redox dye chemistry [38]. The PM-M methodology uses 96-well plates preloaded with various chemical compounds to assess the cell’s capacity to produce energy across diverse metabolic environments, characterized by different energy sources (plates PM-M1 to M4) or external effectors (plates PM-M5 to M8). In our study, we used PM-M1 (carbon-based energy sources), PM-M6 (hormones & metabolic effectors), and PM-M7 (hormones & metabolic effectors). The cells were plated in modified Biolog IF-M1 medium containing penicillin/streptomycin (100 μg/mL), glutamine (0.3 mM), and FBS (5%). The plating medium for PM-M6 and PM-M7 plates contained no FBS but did include glucose (5.5 mM final concentration).

The plates were incubated with 20,000 lymphoblastoid cells per well in 50 μL, maintained at 37 °C in 5% CO_2_ for 48 h. During the 48 h incubation, for PM-M1, the only energy source available to the cells was the chemical in the well. For PM-M6 and PM-M7, the energy source was added glucose; however, metabolic activity was affected by hormones and other effectors. After the first incubation, Biolog Redox Dye Mix MB (10 μL/well) was added, and the plates were incubated under the same conditions for an additional 24 h. During that time, cellular metabolism reduces the tetrazolium dye in the media, resulting in a purple color that corresponds to the amount of NADH generated. The Omnilog (OmniLog, Hayward, CA, USA) measures color every 15 min to provide a kinetic metabolic profile. Further details on the Biolog Phenotype MicroArray were provided by Bochner et al. and Putluri et al. [38,39].

At the end of the 24 h incubation, the plates were analyzed utilizing a microplate spectrophotometer (Infinite M200, Tecan, Mannedorf, Switzerland) with readings at 590 and 750 nm. The first value (A590) corresponds to the redox dye’s highest absorbance peak, and the second value (A750) corresponds to the background noise. The relative absorbance (A590–750) was calculated for each well.

### 2.9. PhenoMetaboDiff and Statistical Analysis

The raw data used for analysis are the endpoint relative absorbance values for each well in the PM-M plate, measured by a spectrophotometer after a 24 h incubation, and the 96 kinetic optical density data points collected over the incubation time with the tetrazolium dye in the Omnilog. Readings were normalized to triplicate absorbance values from the corresponding empty plate (plates run with no cells, just media and dye). Our goal was to identify wells in which the levels of NADH generated by cells in one group differed significantly from those in other groups.

Data processing and computational analyses of the microarray data were performed using RStudio (R version 4.1.2, Boston, MA, USA) and the open-source PhenoMetaboDiff (v1.0.0, RStudio, Boston, MA, USA) software package [40]. The implementation code is publicly available at: https://anonymous.4open.science/r/phenoMetaboDiff-62D4 (accessed 13 September 2025). The PhenoMetaboDiff (PMD) package enables high-throughput identification, visualization, and statistical analysis of metabolomic data generated using the Biolog PM-M system.

Group comparisons were conducted using the Mann–Whitney U test. This nonparametric approach was selected due to the small sample size and the absence of normal distribution assumptions. Resulting *p*-values were adjusted for multiple comparisons using the Benjamini–Hochberg (BH) procedure to control the false discovery rate [41]. An adjusted *p*-value of <0.05 was considered statistically significant.

The web-based platform MetaboAnalyst (V 6.0, Natural Sciences and Engineering Research Council of Canada, Canada) was used for downstream analysis [42]. For wells demonstrating statistically significant differences, pathway enrichment analyses were performed, and pathway maps were generated in R to identify and characterize the biological pathways altered in patients. Heatmaps were also constructed to facilitate visual comparison between groups, enabling assessment of differential patterns and relative signal intensities across key metabolic variables.

## 3. Results

### 3.1. Survey

**Figure 1:**
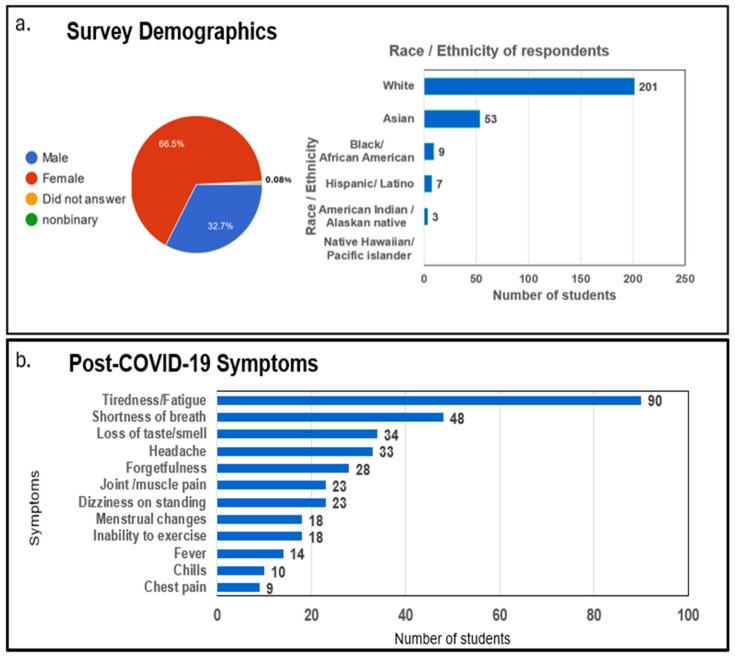
(**a**) Participant demographics, vaccination status, and vaccine details. (**b**) Symptoms lasting 3 months or longer post-C-19 that were not present before C-19.

At the time of sampling, the survey revealed that 81.6% of students had previously received a COVID-19 diagnosis, and 242 (91.3%) had received a vaccination. One hundred and thirty (49%) of the 266 respondents experienced symptoms lasting three or more months that they did not have before contracting C-19, which could be attributable to LC-19. The most frequently reported symptoms were tiredness/fatigue (69.2%), shortness of breath (36.3%), loss of taste/smell (26.2%), and headache (25.4%). Other common symptoms included forgetfulness, dizziness upon standing, menstrual changes, and exercise intolerance (Figure 1).

### 3.2. Choosing the Cohort for the Metabolic Profile

The survey results were used to decide upon the cohort for the metabolic profile. The two groups we identified for the study were:

- Post-COVID-19 Controls (Post-C-19 controls), namely those who recovered from C-19 completely and showed no post-C-19 symptoms.
- LC-19, those with post-C-19 symptoms not attributable to any other condition and those with a clinical diagnosis of LC-19.

We contacted an equal number of survey respondents from each group who indicated a willingness to provide saliva and blood samples for the metabolic profile study. Those who were available were asked to provide saliva and blood samples, and the first 10 respondents in each group were chosen for the study.

The participant demographics are detailed in Table 1. For analysis purposes, we stratified the LC-19 group into three subgroups: LC-19_a, comprising all probable LC-19 patients (those with any LC-19 symptoms); LC-19_b, comprising those with more than 4 symptoms or a confirmed diagnosis; and LC-19_c, comprising those with an official diagnosis given by a qualified healthcare professional (e.g., medical doctor) based on established clinical criteria, evaluation, and documentation.

To assess the effect of SARS-CoV-2 on metabolic processes, we also included a group of 50 healthy controls whose lymphoblastoid cell lines were established before the COVID-19 pandemic. These individuals had no exposure to SARS-CoV-2 and are referred to as Pre-COVID-19 controls (Pre-C-19 controls).

### 3.3. Salivary Virus Status

All the saliva samples from the volunteers were tested for C-19 and found negative, indicating the absence of active infection at the time of blood donation. To exclude potential metabolic effects attributable to opportunistic infections in the LC-19 cohort, all study samples were screened for EBV and human herpesvirus 6 (HHV-6) infection. Some samples tested positive for EBV; however, the positivity did not show a consistent pattern across the aforementioned groups. None of the saliva samples tested positive for Human herpesvirus 6 (HHV-6).

### 3.4. Creation of the Lymphoblastoid Cell Lines (LCLs)

Lymphoblastoid cell lines (LCLs) are a well-established in vitro model for investigating cellular metabolic phenotypes. Because LCLs are derived from B lymphocytes and retain donor-specific genetic and epigenetic characteristics, they provide a stable and reproducible platform for examining immune-metabolic alterations. In the context of Long COVID, where immune dysregulation and persistent metabolic abnormalities have been implicated, LCLs offer a controlled system to evaluate intrinsic metabolic signatures while minimizing confounding influences present in whole-blood or plasma-based analyses. LCLs created by transducing PBMCs with EBV showed a marked difference between the controls and the LC-19 group. The success rate for LCL creation in the LC-19 groups was 100%, with all 12 blood samples yielding healthy cell lines. On the other hand, in the control group, the success rate was only 72%, with only 13 of 18 blood samples yielding successful cell line creation. We included the first ten cell lines in each group that yielded enough cells for the experimental study on a random, first-served basis, a well-established and ethically accepted strategy for samples in experimental studies.

### 3.5. Biolog Phenotype Mammalian Microarrays

#### 3.5.1. PM-M1 Plate (Carbon Energy Sources)

PM-M1 has a range of diverse carbon-based energy sources, including simple sugars, polysaccharides, and carboxylic acids. These are substrates and intermediary compounds in cells’ energy production pathways (glycolysis, the citric acid cycle, and oxidative phosphorylation), and the cell’s ability to utilize them helps elucidate these pathways.

**Figure 2:**
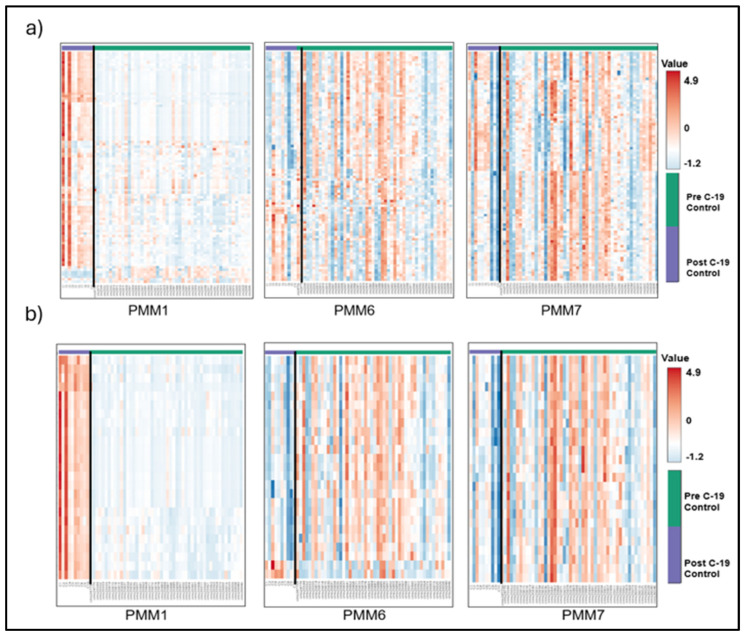
Hierarchical clustering heatmap of plates PM-M1 (energy sources), PM-M6 (hormones and metabolic effectors), PM-M7 (hormones and metabolic effectors), comparing pre-C-19 (green) and post-C-19 (purple) controls, (**a**) in the whole plate, (**b**) the top 25 significant wells.

To assess the effect of SARS-CoV-2 infection on metabolism, we compared cellular utilization of energy sources between the pre-C-19 and post-C-19 control groups, with 76 of 93 wells (81.7%) showing a significant difference (Supplementary Materials File S1, p. 5). In all the wells, the post-C-19 control group shows a dramatic increase in energy utilization by a factor of 2–3 (Figure 2a). Further examination indicated that the wells with significant differences (*p* < 0.05) contain substrates and intermediary compounds in the energy production pathways: glycolysis, citric acid cycle, ketone bodies, oxidative phosphorylation, and pathways utilizing alternative carbon-based energy sources, such as carboxylic acids or complex polysaccharides (Supplementary Materials File S1, p. 5). The increased utilization of these compounds indicates that the SARS-CoV-2 infection changes resting cellular activity levels, leading to accelerated energy production rates that can be interpreted as a metabolic shift in response to distress, similar to that observed during inflammatory responses (Supplementary Materials Table S5).

Hierarchical clustering heatmaps for plates PM-M1, PM-M6, and PM-M7 comparing post-C-19 controls with pre-C-19 controls provide a visual representation of the notable distinctions between the two groups (Figure 2).

**Figure 3:**
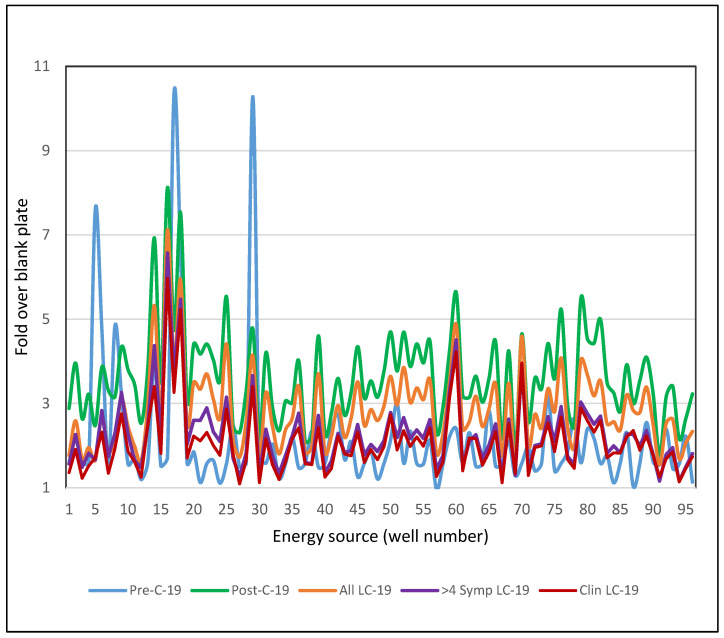
Utilization of energy sources in the PM-M1 plate by the different cohorts. The *x*-axis shows the well numbers, corresponding to different energy sources analyzed by the Biolog system (Biolog, Hayward, CA, USA).

For plate PM-M1, comparison of pre-C-19 controls with the LC-19 group overall (All LC-19) revealed that 50 out of 93 (53.8%) compounds led to higher NADH levels in the LC-19 cells, confirming the stimulatory effect of the SARS-CoV-2 infection on the major metabolic pathways involved in energy production (Figure 3). However, 8 wells showed increased NADH levels in the pre-C-19 controls, including glucose, dextrin, glycogen, and mannose, an epimer of glucose. Interestingly, glucose, dextrin, and mannose showed similarly significant reductions in energy production in post-C-19 cells compared to pre-C-19 cells. This trend suggests that some pathways utilizing high-efficiency energy sources have been saturated in individuals undergoing C-19, and exposure to these energy sources fails to generate the same amount of energy as in individuals who have never been exposed to the SARS-CoV-2 virus.

When we stratified the LC-19 cohort by symptoms, we noticed notable and informative differences. The subgroup of LC-19 with >4 symptoms showed 36 compounds (38.7%) with lower energy levels than pre-C-19 controls, and 15 (16.1%) with higher levels. Interestingly, among the compounds associated with reduced NADH production, the usual glucose, dextrin, glycogen, and mannose were accompanied by other carbohydrates (galactose, fucose, sorbose, xylitol), a key intermediate of the Krebs cycle (α-keto-glutaric acid), and carboxylic acids (acetic acid and butyric acid), suggesting an exacerbation of the exhaustion of diverse energetic pathways. The subgroup with an official LC-19 diagnosis showed only 9 (9.7%) compounds with increased energy production and 4 (4.3%) with decreased energy production (glucose, mannose, fucose, and α-keto-glutaric acid (Supplementary Materials File S1, p. 17).

Since data indicate significant changes in energy metabolism due to SARS-CoV-2 infection, we also compared the LC-19 group with post-C-19 controls. While there is a discernible difference in energy resource utilization between groups, combining all LC-19 patients into a single group (LC-19_a) did not yield more informative profiles than symptom-based stratification. This trend was consistent across all PM-M plates used in the study, indicating that the metabolic perturbations induced by SARS-CoV-2 infection persist beyond the acute phase of the disease (Supplementary Materials File S1, p. 7). The LC-19_b (>4 symptoms) versus post-C-19 controls comparison highlighted statistically significant differences in 69 of the 93 chemicals (74.2%) (Supplementary Materials File S1, p. 9). Finally, 60 of the 93 chemicals (64.5%) showed statistically significant differences between the LC-19_c (official diagnosis) group and post-C-19 controls (Supplementary Materials File S1, p. 11). Although across all three strata, the LC-19 patient group showed decreased utilization of metabolites compared with the Post-C-19 controls, our findings clearly demonstrate that individuals with greater symptom burden or an official diagnosis exhibit greater dysfunction in cellular energy metabolism.

Across all LC-19 subgroups, the utilization of compounds in the cellular energy metabolism pathway is higher than in pre-C-19 controls but lower than in post-C-19 controls, reflecting disruptions in cellular energy pathways. This finding suggests that cells respond to SARS-CoV-2 by increasing the utilization of energy sources, but, over time, are unable to sustain the stress response, and their ability to metabolize these compounds decreases (Figure 3).

**Figure 4:**
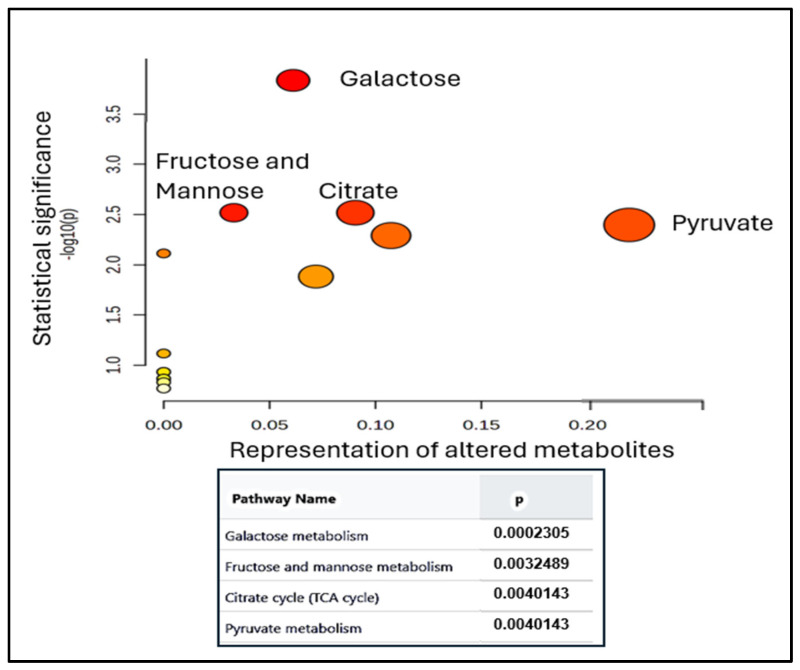
Pathway analysis PM-M1 (energy sources): LC-19 and post-C-19 controls. The color of circles represented significance (*p*-values), and their size indicated the degree of representation of the metabolite in the enriched pathways.

Using the pathway analysis feature of Metaboanalyst, a web-based platform used for metabolomic data analysis, we mapped the compounds whose utilization showed significant change between the LC-19 and control groups. Pathway map analysis measures both the representation of altered metabolites within the pathway (*x*-axis) and the statistical significance of pathway enrichment (*y*-axis). Analysis for the PM-M1 plate metabolic profile identified four major pathways exhibiting significant alterations: galactose metabolism, pyruvate metabolism, fructose and mannose metabolism, and the citric acid cycle. Changes in activity in all the pathways were significant (Figure 4).

#### 3.5.2. PM-M6 and PM-M7 Plates (Metabolic Effectors)

These two PM-M microplates differ from PM-M1 since they measure the metabolic response to various metabolic effectors in the presence of a constant energy source (glucose). Therefore, the data gathered from these plates reveal a dynamic metabolic process involving one or more pathways for each compound. Moreover, each chemical is titrated at six increasing concentrations, enabling us to explore dose-dependent responses that mimic different metabolic scenarios.

PM-M6 microplates are coated with different hormones and other metabolic effectors, and each chemical is present at 6 different concentrations. The measurement of NADH produced in these wells shows how the cells metabolize glucose in the plating medium while the effector is present. Responses to growth factors and cytokines indicate alterations in signaling pathways that could be linked to immune dysregulation and repair mechanisms.

A comparison of the pre-C-19 and post-C-19 controls showed that 70 of the 90 wells (77.8%) were remarkably different, even if they were significant for the unadjusted *p*-values but not for the adjusted ones (Supplementary Materials Table S6). Most wells showed that, compared to pre-C-19 controls, post-C-19 wells exhibited reduced NADH production, indicating a cellular lack of response to hormones and other effectors. PMM6 heat maps in Figure 2 visualize this difference between the groups, suggesting potential differences in sensitivity or signaling efficiency between the two groups.

Comparing the LC-19 group with pre-C-19 controls, stratified by symptoms, revealed some interesting differences. A comparison of LC-19_a (all probable LC-19) with pre-C-19 controls showed a significant difference between the two groups, with 66 wells showing lower NADH levels in LC-19 cells across at least two concentrations for each compound (Supplementary Materials File S1, p. 27). It is worth noting that one well showed higher NADH production in LC-19: 3-isobutyl-1-methylxanthine, which also had three concentration points with lower NADH. When compared with pre-C-19 controls, LC-19_b (>4 symptoms) cells showed statistically significant reductions in NADH levels in 77 of the 90 wells (85.6%), encompassing at least 3 concentrations for each metabolic effector (Supplementary Materials File S1, p. 29). The trend was even more evident when we compared LC-19_c (official diagnosis) with pre-C-19 controls: 84 of the 90 wells (93.3%) showed significantly reduced energy production in LC-19_c cells (Supplementary Materials File S1, p. 31). The LC-19 cells in all groups exhibited less than half the activity of the control cells in response to all the tested metabolic effectors. The diminished or disrupted responses of all LC-19 groups to the hormones and signaling molecules present in the wells indicate that SARS-CoV-2 infection reduces cells’ sensitivity to these signaling molecules.

**Figure 5:**
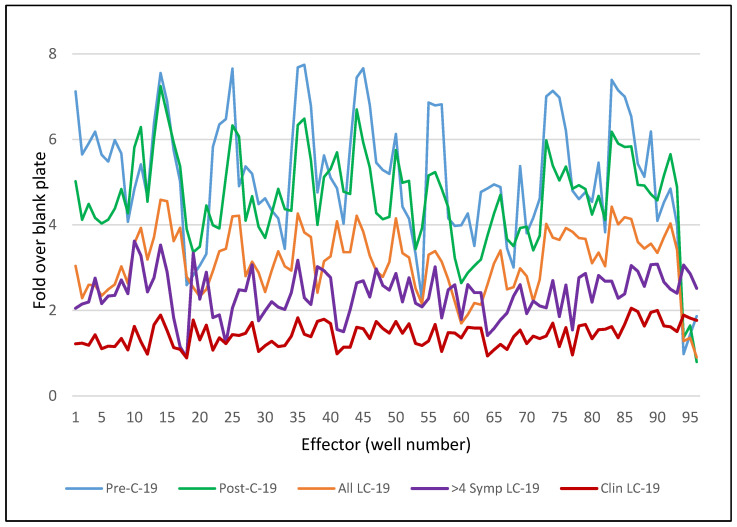
Response to effectors in the PM-M6 (hormones and metabolic effectors), plate by the different cohorts. The *x*-axis represents the well numbers, which correspond to different hormones and metabolic effectors analyzed by the Biolog system.

Comparing LC-19 patients with the post-C-19 group, across all LC-19 subgroups, cellular response to compounds in the PM-M6 plate indicated reduced response to effectors. There is a graded decrease in response, with the complete LC-19 group of 10 samples showing a slight reduction that failed to reach statistical significance for the adjusted *p*-values, while a substantially greater reduction in those with more than 4 symptoms (75/90 wells, 83.3%, encompassing all compounds except dibutyryl-cAMP) and in those with a clinical LC-19 diagnosis (78/90 wells, 86.7%, including at least three concentrations for each metabolic effector) (Figure 5).

The post-C-19 group shows a reduced response compared to the pre-C-19 group, and this response is further reduced in the LC-19 cohort. This finding suggests that cells respond to SARS-CoV-2 by exhibiting a decreased response to effectors; however, over time, even this diminished response cannot be maintained, with greater decreases observed in the LC-19 subgroups and the least response shown by those with a clinical LC-19 diagnosis.

PM-M7 microplates are coated with cytokines, growth factors, hormones, and other metabolic effectors, different from those in plate PM-M6. A comparison of pre-C-19 and post-C-19 controls showed significant differences in 65 of 90 wells (72.2%) (Supplementary Materials Table S7). In all wells, post-C-19 cells produce lower levels of NADH, indicating reduced metabolic activity. A heat map comparing the two controls shows that the post-C-19 response to the hormones and effectors was lower than the pre-C-19 one (Figure 1b).

The LC-19_a (all LC-19) showed significantly lower metabolic response than pre-C-19 controls in 65 of 90 wells (72.2%), including at least one concentration for each metabolic effector in PM-M7. Comparing pre-C-19 controls with the LC-19 group, stratified by subgroups, revealed differential cellular activation patterns. The LC-19_b (>4 symptoms) showed significantly reduced energetic response to 58 of the 90 chemicals (64.4%) as compared to pre-C-19. Since LCLs are obtained from primary immune cells, it is noteworthy that LC-19_b LCLs exhibited significantly reduced responses to any concentration of two major pro-inflammatory cytokines: interleukin 1 beta (IL-1β) and interleukin 6 (IL-6). These findings may explain the greater predisposition to reinfections or comorbidities, or the manifestation of a broader range of COVID-related symptoms.

On the other hand, the LC-19_c (official diagnosis) cells exhibited less than half the activity of the pre-C-19 cells in a vast majority of wells, although no compound reached adjusted *p*-values < 0.05.

**Figure 6:**
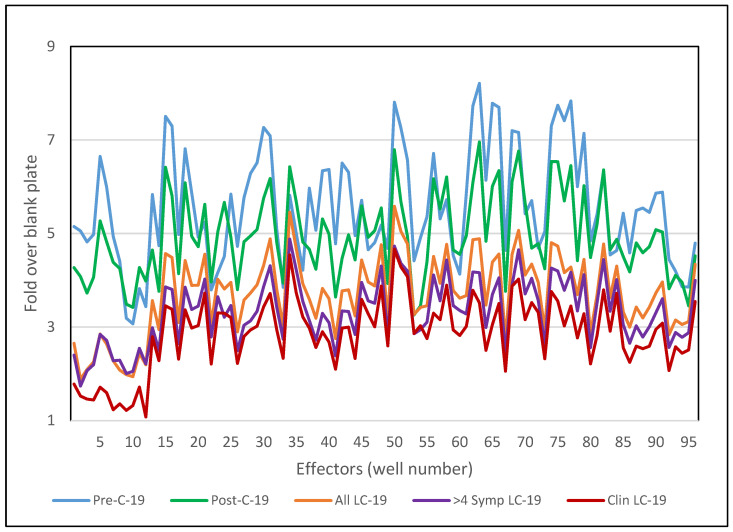
Response to effectors in the PM-M7 (hormones and metabolic effectors) plate by the different cohorts. The *x*-axis represents the well numbers, which correspond to different hormones and metabolic effectors analyzed by the Biolog system.

Comparison of the stratified LC-19 groups and the Pre-C-19 controls, as shown on the PM-M7 plate graphed in Figure 6, provides an excellent visual representation of the cellular responses to the compounds in the wells. The LC-19 cells overall showed a lack of response to the growth factors and effectors within the wells. The difference becomes more pronounced with stratification, and cells from individuals with >4 symptoms and those with an official diagnosis emerge as the most unresponsive to the effectors.

Comparing the LC-19 patients with the post-C-19 group in response to compounds on the PM-M7 plate again indicates a reduced response to the effectors, with unadjusted *p*-values reaching statistical significance but not the adjusted ones. Similarly to the response of cells to the effectors in the PM-M6 plate, the post-C-19 group exhibits a reduced response compared to the pre-C-19 group, although no wells reached significance for unadjusted *p*-values, and this response is further reduced in the LC-19 cohort. There is also a graded decrease in response, with the complete LC-19 group of 10 samples showing a slight reduction, followed by a greater reduction in those with more than 4 symptoms, and the least response in those with a clinical LC-19 diagnosis (Figure 6). Although the difference is not statistically significant, the trend is revealing: the LC-19 group is unable to respond, which is correlated with symptom burden. This finding suggests that cells react to SARS-CoV-2 by exhibiting decreased responses to effectors; however, over time, even this response cannot be maintained, with greater decreases observed in the LC-19 cohort and the least response observed in those with a clinical LC-19 diagnosis (Supplementary Materials File S1, pp. 35, 37, 39).

## 4. Discussion

LC-19 has emerged as a significant health concern due to the lack of clear diagnostic guidelines. Several approaches to achieve this have been proposed, including metabolomics-based machine learning modeling of the disease and biomarker discovery [43,44,45]. This is particularly crucial in young adults, a group traditionally considered to be at lower risk for chronic conditions. The lingering effects can compromise academic performance, workforce participation, and overall quality of life. Given that young adults represent a critical segment of the future workforce, the long-term consequences of reduced productivity and health may be hard to estimate.

The survey results were striking, with a large proportion (44–49%) of otherwise healthy students potentially showing symptoms indicative of LC-19. Consistent with the published literature, our survey revealed that a majority of those affected experienced musculoskeletal sequelae, with more females than males reporting symptoms [46,47]. Long COVID has a significant impact on the health, well-being, and productivity of the young adult population [46]. This high prevalence is noteworthy, as reduced or compromised work capacity in this demographic translates into substantial long-term economic losses.

The dramatic difference in LCL generation efficiency we observed between the LC-19 and control groups (68% vs. 100%) in our study was unexpected. This difference may be explained by LC-19-induced immune exhaustion, in which the immune system is too depleted to respond effectively to new threats, as seen with EBV in this instance [47]. EBV-mediated lymphocyte immortalization depends on the establishment of a latent episomal viral genome and sustained host cell proliferation, rather than viral genome integration into host DNA [48,49]. It is unlikely that immune exhaustion would interfere with EBV infection and maintenance in the cell, as these processes are primarily virus-driven and mediated through CD21-dependent infection and EBNA1-supported episome maintenance [50]. Instead, immune exhaustion is more likely to affect post-infection lymphoblastoid growth by allowing unimpaired host cell proliferation and survival rate. Chronically activated or exhausted lymphocytes exhibit metabolic dysfunction and altered cell-cycle regulation; additionally, persistent exposure to inflammatory cytokines, such as IL-6, promotes exhaustion phenotypes [51,52,53]. The increased efficiency of lymphoblastoid cell line generation in exhausted immune states, as seen in LC-19, most likely reflects enhanced post-infection proliferation rather than more effective EBV infection or episome maintenance. A recently published paper by Chen-Camato et al. showed that, in patients with LC-19, differential gene expression analysis of PBMCs revealed dysregulated pathways related to antigen presentation, cytokine signaling, and immune activation [54]. Altered expression of genes involved in B-cell development, macrophage activation, and cytokine-mediated signaling further supports the presence of both adaptive and innate immune perturbations. LC-19 patients also have chronic inflammation, which may account for the body’s reduced ability to mount a coordinated defense against new infections [16,22]. In children with LC-19, hyperinflammation caused by the C-19 virus has been reported to cause periods of immune hypo-responsiveness [55].

Unlike several prior studies, we did not observe a resurgence of latent viral expression, such as EBV or HHV-6, in our cohort [21]. This discrepancy may be attributable to the limited sample size, which could have reduced our statistical power to detect low-level or heterogeneous viral reactivation. Consequently, larger and longitudinal studies will be necessary to more definitively assess the role of latent viral resurgence in this context.

Metabolic phenotyping offers insights at the whole-organism level and, in our case, may provide insights into LC-19 symptomatology and help identify and dissect the metabolic basis of different phenotypes of this complex disease. The metabolic profile in the LC-19 cohort offers insight into the human response to SARS-CoV-2 and the development of chronic disease. Overall, the data collected from the PM-M arrays delineate distinct profiles for individuals who contracted SARS-CoV-2 and developed Long COVID (LC-19) and for those who did not (post-C-19 controls). While both groups showed higher energy production for most energy sources, only the LC-19 cells generated strikingly reduced NADH levels in response to metabolic effectors.

The increased response to PM-M1 plate substrates, with higher metabolic activity even in control cells after the pandemic, may indicate a cellular response to the stress of the C-19 viral infection. This is understandable, as almost everyone has been exposed to the virus and is likely constantly re-exposed to it at subclinical levels. C-19 Surveillance Data in the United States indicates the presence of the virus in the body long after a negative C-19 test [56]. This continual exposure to the virus would lead the human body to constantly respond to stress by altering the metabolic profile of energy utilization, as reflected in our post-C-19 control population cells.

Metabolic activity in LC-19 cells is lower than that of post-C-19 control cells, indicating abnormalities in the glycolytic pathway and the TCA cycle, as well as compromised mitochondrial energy production. Moreover, energy production in response to metabolic effectors, as shown by the findings from PM-6 and PM-M7 arrays, is similar in pre- and post-C-19 cells but is dramatically lower in the LC-19 cohort. This metabolic trend demonstrates attenuated energy utilization by the LC-19 cohort, suggesting dysfunction in cellular bioenergetic pathways, which could explain fatigue and muscle weakness, the most common symptoms observed in LC-19 patients [9].

In line with evidence that mitochondrial dysfunction underlies hallmark symptoms of long COVID—such as cognitive impairment, fatigue, muscle weakness, breathlessness, and cardiac manifestations, our findings in the LC-19 cohort demonstrate impaired energy substrate utilization, consistent with deficits in bioenergetics, oxidative stress, immune dysregulation, and vascular/endothelial dysfunction. A recent study by Charles et al. (2025) also clearly indicates that mitochondrial dysfunction in LC-19 patients is reflected in the PBMC metabolic activity, with the degree of dysfunction parallel with the increased severity of LC-19 [57]. Our study also reiterates that mitochondrial dysfunction is a biomarker for LC-19 and could be a valuable tool for diagnosing the condition. Consistent with our observations, multiple studies have recognized altered energy utilization and fatigue as defining features of LC-19. Under these conditions, assessment of respiratory capacity may be essential for both diagnostic evaluation and the monitoring of treatment efficacy in LC-19 [58].

The decreased metabolic activity in response to hormones, cytokines, and other effectors in both the PM-M6 and PM-M7 plates by the cells of the LC-19 cohort, compared to the post-C-19 control cohort, indicates reduced sensitivity/adaptability to signaling molecules, impaired signaling capacity, and possible cellular dysfunction. The observed lack of responsiveness to IL-6 and TNF-α in LC-19 cells raises the possibility of a compensatory host response involving increased cytokine production, which may account for the elevated IL-6 levels reported by Simón-Rueda et al. and Jacobs et al. [59,60]. The cells diminished or disrupted response to the metabolic effectors present in the PM-M6 and PM-M7 plate may indicate a disease-related metabolic signature. This reduced response suggests impaired receptor-mediated signaling and diminished cellular adaptability to extrinsic regulatory cues. The compromised signal transduction pathways and altered cellular responsiveness reflect a loss of regulatory control, consistent with dysregulated intercellular communication and impaired homeostatic signaling.

Consistent with previous reports, our results indicate that metabolic imbalances correlate with symptom burden, such that individuals with greater symptom burden exhibit more extensive metabolic profile changes [61]. It is noteworthy to report that stratification of the LC-19 groups for comparison yields distinct and meaningful differences. In many comparisons, pooling all LC-19 samples, regardless of the number of symptoms, yields fewer, less significant differences, whereas the group with more than 4 symptoms shows larger, more marked differences. Another critical point is that the group with more than 4 symptoms is very similar to the official LC-19 diagnosis group in terms of metabolic profile.

The long-term effects of viral infections have been recognized for over a century, with the term “post-viral syndrome” used to describe persistent physiological and neurological issues that can persist for months or even years after the initial illness resolves. This is not a new phenomenon but a well-documented aspect of virology.

However, what is unique about the LC-19 situation is the number of people who were exposed to the LC-19 virus in the same time window. This unique situation, where clear cause-and-effect is evident, enables researchers to examine patient metabolic data collectively to identify biomarkers for diagnosis, study disease mechanisms, pinpoint targets for therapeutic intervention, and accelerate exploration of other genetic and environmental factors.

Researchers have investigated predictors and biomarkers of LC-19; however, no definitive indicator currently exists to predict susceptibility or confirm the diagnosis [44]. Rather than a single marker, it may be better to examine a profile or trend that can accurately predict [62]. A multi-marker approach would increase both sensitivity and specificity, a method like that currently used in the diagnosis of other diseases [63].

## 5. Conclusions

Our small study has identified unique metabolic profiles associated with different stages of SARS-CoV-2 infection. Collectively, these findings suggest that immune dysregulation and features consistent with immune exhaustion may contribute to the persistence and progression of long COVID. In COVID-19, accumulating evidence associates exhausted T-cell phenotypes with adverse clinical outcomes, including severe disease, delayed viral clearance, and persistent symptoms linked to Long COVID [64]. This also suggests the need for further research with larger, better-defined cohorts to better characterize the correlations among disease stages, clinical symptoms, and metabolic abnormalities. In our study, we did not stratify the LC-19 cohort by the infecting SARS-CoV-2 variant. We acknowledge that differences among infecting viral variants could introduce variability and may have influenced the observed outcomes. Infection with different SARS-CoV-2 variants is associated with varying patterns of LC-19 symptoms, suggesting that viral genetic differences may modify post-acute outcomes [65].

Validation of metabolic and molecular biomarkers offers the potential for earlier diagnosis, prediction of disease severity, and development of personalized treatment strategies. Additionally, the large number of LC-19 patients will enable us to conduct large-scale, comprehensive studies to more precisely delineate the long-term impact of SARS-CoV-2 on human health. A deeper understanding of the underlying pathophysiology, including immune dysregulation, mitochondrial dysfunction, and involvement of the autonomic nervous system, will be essential for understanding disease diagnosis and progression.

Longitudinal and extensive cohort studies would help to characterize disease trajectories, identify risk factors, and evaluate the impact of therapeutic interventions. Integrating multi-omics and systems biology approaches to reveal comprehensive disease signatures and novel therapeutic targets, along with studies on health policy, access to care, and patient-centered models, must be at the forefront of all LC-19 patient care for optimal results. Together, these research directions can fill knowledge gaps, enhance clinical management, and inform effective strategies to address the complex and evolving challenges of LC-19.

## Figures and Tables

**Table 1:** Demographic information of the study participants.

Demographic Group	LC-19 Status	
	Control	LC-19
Total	10	10
Gender		
Male	5	3
Female	5	7
Age (years)		
Range	21–29	21–46
Mean	25.4	25.6
LC-19 status		
Long COVID Dx		3
>4 Symptoms		3
<3 Symptoms		6
Demographics		
White	10	8
Black	0	1
Asian	0	1
LCL Creation		
Success	72%	100%
Symptoms		
Tiredness		12
Shortness of Breath		9
Inability to exercise		7
Headache		7
Joint/muscle pain		6
Forgetfulness		5
Dizziness upon standing		3
Chest pain		2
Menstrual changes		4
Loss of taste/smell		2

## Data Availability

The data are available upon reasonable request.

## Supplementary Materials

The following supporting information can be downloaded at: https://www.mdpi.com/article/10.3390/idr18030050/s1, Table S1: Reagents for COVID-19 diagnostic assay; Table S2: Touchdown RT-qPCR protocol. Thermocycling conditions for one-step RT-qPCR SARS-CoV-2 diagnostic assay; Table S3: Reagents for EBV and HHV-6 PCR assay; Table S4: Touchdown qPCR protocol. Thermocycling conditions for PCR for EBV and HHV-6; Table S5: PM-M1: Significant (*p* < 0.05) changes in metabolic activity in the utilization of carbon and nitrogen energy sources in PM-M1 by individuals with LC-19 compared to pre-C-19 controls; Table S6: PM-M6: Wells with significant (*p* < 0.05) differences in metabolic activity in response to hormones and modulators in cells from individuals with the LC-19 compared to pre-C-19 controls; Table S7: PM-M7: Wells with significant (*p* < 0.05) changes in metabolic activity in response to hormones and modulators in cells from individuals with the LC-19 compared to pre-C-19 controls; File S1: Biolog data.

## Abbreviations

The following abbreviations are used in this manuscript:

C-19	COVID-19
LC-19	Long COVID-19
