# Supplementary material for: Cellular Metabolic Signatures of Long COVID-19
Source: Infect Dis Rep. 2026 May 26;18(3):50. doi: 10.3390/idr18030050 (PMC13299657; doi:10.3390/idr18030050)
Supplement: Supplementary file 1 [file idr-18-00050-s001.zip › Supp. Folder Biolog data.pdf]

INDEX

	Page
Index	1
Well contents of PM-M1	2
Well contents of PM-M6	3
Well contents of PM-M7	4
PMM1_Post-C-19 Control to Pre-C-19 Control	5
PMM1_Post-C-19 Control to LC-19	7
PMM1_Post-C-19 Control to LC-19 more than 4 symptoms	9
PMM1_Post-C-19 Control to LC-19 Official Diagnosis	11
PMM1_Pre-C-19 Control to LC-19	13
PMM1_Pre-C-19 Control to LC-19 more than 4 symptoms	15
PMM1_Pre-C-19 Control to LC-19 Official Diagnosis	17
PMM6_Post-C-19 Control to Pre-C-19 Control	19
PMM6_Post-C-19 Control to LC-19	21
PMM6_Post-C-19 Control to LC-19 more than 4 symptoms	23
PMM6_Post-C-19 Control to LC-19 Official Diagnosis	25
PMM6_Pre-C-19 Control to LC-19	27
PMM6_Pre-C-19 Control to LC-19 more than 4 symptoms	29
PMM6_Pre-C-19 Control to LC-19 Official Diagnosis	31
PMM7_Post-C-19 Control to Pre-C-19 Control	33
PMM7_Post-C-19 Control to LC-19	35
PMM7_Post-C-19 Control to LC-19 more than 4 symptoms	37
PMM7_Post-C-19 Control to LC-19 Official Diagnosis	39
PMM7_Pre-C-19 Control to LC-19	41
PMM7_Pre-C-19 Control to LC-19 more than 4 symptoms	43
PMM7_Pre-C-19 Control to LC-19 Official Diagnosis	45

Well contents of PM-M1

Well number	Well location	Compound
1	A01	NegativeControl
2	A02	NegativeControl
3	A03	NegativeControl
4	A04	a-Cyclodextrin
5	A05	Dextrin
6	A06	Glycogen
7	A07	Maltitol
8	A08	Maltotriose
9	A09	Maltose
10	A10	D-Trehalose
11	A11	D-Cellobiose
12	A12	Gentiobiose
13	B01	D-Glucose-6-Phosphate
14	B02	D-Glucose-1-Phosphate
15	B03	L-Glucose
16	B04	D-(+)-Glucose
17	B05	D-(+)-Glucose
18	B06	D-(+)-Glucose
19	B07	3-MethylGlucose
20	B08	a-Methyl-D-Glucoside
21	B09	b-Methyl-D-Glucoside
22	B10	Salicin
23	B11	D-Sorbitol
24	B12	N-Acetyl-D-Glucosamine
25	C01	D-GlucosaminicAcid
26	C02	D-GlucuronicAcid
27	C03	ChondroitinSulfateC
28	C04	Mannan
29	C05	D-Mannose
30	C06	a-Methyl-D-Mannoside
31	C07	D-Mannitol
32	C08	N-Acetyl-D-Mannosamine
33	C09	D-Melezitose
34	C10	Sucrose
35	C11	Palatinose
36	C12	Turanose
37	D01	D-Tagatose
38	D02	L-Sorbose
39	D03	L-Rhamnose
40	D04	L-Fucose
41	D05	D-Fucose
42	D06	D-Fructose-6-Phosphate
43	D07	D-Fructose
44	D08	Stachyose
45	D09	D-Raffinose
46	D10	Lactitol
47	D11	Lactulose
48	D12	a-D-Lactose

Well number	Well location	Compound
49	E01	MelibionicAcid
50	E02	D-Melibiose
51	E03	D-Galactose
52	E04	a-Methyl-D-Galactoside
53	E05	b-Methyl-D-Galactoside
54	E06	n-acetyl-neuraminicacid
55	E07	Pectin
56	E08	Sedoheptulosan
57	E09	Thymidine
58	E10	Uridine
59	E11	Adenosine
60	E12	Inosine
61	F01	Adonitol
62	F02	L-Arabinose
63	F03	D-Arabinose
64	F04	b-Methyl-D-Xyloside
65	F05	Xylitol
66	F06	m-Inositol
67	F07	i-Erythritol
68	F08	1,2-Propanediol
69	F09	2-Aminoethanol
70	F10	D,L-a-GlycerolPhosphate
71	F11	Glycerol
72	F12	CitricAcid
73	G01	TricarballylicAcid
74	G02	L-LacticAcid(DL)
75	G03	MethylD-Lactate
76	G04	MethylPyruvate
77	G05	PyruvicAcid
78	G06	a-Keto-GlutaricAcid
79	G07	SuccinamicAcid
80	G08	SuccinicAcid
81	G09	MonoMethylSuccinate
82	G10	L-MalicAcid
83	G11	D-MalicAcid
84	G12	m-TartaricAcid
85	H01	AcetoaceticAcid
86	H02	g-AminoButyricAcid
87	H03	a-Keto-ButyricAcid
88	H04	D,L-a-Hydroxy-ButyricAcid
89	H05	b-Hydroxy-ButyricAcid
90	H06	g-Hydroxy-ButyricAcid
91	H07	ButyricAcid
92	H08	2,3-Butanediol
93	H09	3-Hydroxy2-Butanone
94	H10	PropionicAcid
95	H11	AceticAcid
96	H12	HexanoicAcid

Well contents of PM-M6

Well number	Well location	Compound
1	A01	NegativeControl
2	A02	NegativeControl
3	A03	NegativeControl
4	A04	NegativeControl
5	A05	NegativeControl
6	A06	NegativeControl
7	A07	Dibutyryl-cAMP
8	A08	Dibutyryl-cAMP
9	A09	Dibutyryl-cAMP
10	A10	Dibutyryl-cAMP
11	A11	Dibutyryl-cAMP
12	A12	Dibutyryl-cAMP
13	B01	3-Isobutyl-1-Methylxanthine
14	B02	3-Isobutyl-1-Methylxanthine
15	B03	3-Isobutyl-1-Methylxanthine
16	B04	3-Isobutyl-1-Methylxanthine
17	B05	3-Isobutyl-1-Methylxanthine
18	B06	3-Isobutyl-1-Methylxanthine
19	B07	Caffeine
20	B08	Caffeine
21	B09	Caffeine
22	B10	Caffeine
23	B11	Caffeine
24	B12	Caffeine
25	C01	Epinephrine
26	C02	Epinephrine
27	C03	Epinephrine
28	C04	Epinephrine
29	C05	Epinephrine
30	C06	Epinephrine
31	C07	Norepinephrine
32	C08	Norepinephrine
33	C09	Norepinephrine
34	C10	Norepinephrine
35	C11	Norepinephrine
36	C12	Norepinephrine
37	D01	L-Leucine
38	D02	L-Leucine
39	D03	L-Leucine
40	D04	L-Leucine
41	D05	L-Leucine
42	D06	L-Leucine
43	D07	Creatine
44	D08	Creatine
45	D09	Creatine
46	D10	Creatine
47	D11	Creatine
48	D12	Creatine

Well number	Well location	Compound
49	E01	Triiodothyronine
50	E02	Triiodothyronine
51	E03	Triiodothyronine
52	E04	Triiodothyronine
53	E05	Triiodothyronine
54	E06	Triiodothyronine
55	E07	Thyroxine
56	E08	Thyroxine
57	E09	Thyroxine
58	E10	Thyroxine
59	E11	Thyroxine
60	E12	Thyroxine
61	F01	Dexamethasone
62	F02	Dexamethasone
63	F03	Dexamethasone
64	F04	Dexamethasone
65	F05	Dexamethasone
66	F06	Dexamethasone
67	F07	Hydrocortisone
68	F08	Hydrocortisone
69	F09	Hydrocortisone
70	F10	Hydrocortisone
71	F11	Hydrocortisone
72	F12	Hydrocortisone
73	G01	Progesterone
74	G02	Progesterone
75	G03	Progesterone
76	G04	Progesterone
77	G05	Progesterone
78	G06	Progesterone
79	G07	beta-Estradiol
80	G08	beta-Estradiol
81	G09	beta-Estradiol
82	G10	beta-Estradiol
83	G11	beta-Estradiol
84	G12	beta-Estradiol
85	H01	4,5-a-Dihydrotestosterone
86	H02	4,5-a-Dihydrotestosterone
87	H03	4,5-a-Dihydrotestosterone
88	H04	4,5-a-Dihydrotestosterone
89	H05	4,5-a-Dihydrotestosterone
90	H06	4,5-a-Dihydrotestosterone
91	H07	Aldosterone
92	H08	Aldosterone
93	H09	Aldosterone
94	H10	Aldosterone
95	H11	Aldosterone
96	H12	Aldosterone

Well contents of PM-M7

Well number	Well location	Compound	Well number	Well location	Compound
1	A01	NegativeControl	49	E01	hGH(Somatotropin)
2	A02	NegativeControl	50	E02	hGH(Somatotropin)
3	A03	NegativeControl	51	E03	hGH(Somatotropin)
4	A04	NegativeControl	52	E04	hGH(Somatotropin)
5	A05	NegativeControl	53	E05	hGH(Somatotropin)
6	A06	NegativeControl	54	E06	hGH(Somatotropin)
7	A07	Insulin	55	E07	IGF-I
8	A08	Insulin	56	E08	IGF-I
9	A09	Insulin	57	E09	IGF-I
10	A10	Insulin	58	E10	IGF-I
11	A11	Insulin	59	E11	IGF-I
12	A12	Insulin	60	E12	IGF-I
13	B01	Resistin	61	F01	FGF-1(aFGF)
14	B02	Resistin	62	F02	FGF-1(aFGF)
15	B03	Resistin	63	F03	FGF-1(aFGF)
16	B04	Resistin	64	F04	FGF-1(aFGF)
17	B05	Resistin	65	F05	FGF-1(aFGF)
18	B06	Resistin	66	F06	FGF-1(aFGF)
19	B07	Glucagon	67	F07	PDGF-AB
20	B08	Glucagon	68	F08	PDGF-AB
21	B09	Glucagon	69	F09	PDGF-AB
22	B10	Glucagon	70	F10	PDGF-AB
23	B11	Glucagon	71	F11	PDGF-AB
24	B12	Glucagon	72	F12	PDGF-AB
25	C01	Ghrelin	73	G01	IL-1beta
26	C02	Ghrelin	74	G02	IL-1beta
27	C03	Ghrelin	75	G03	IL-1beta
28	C04	Ghrelin	76	G04	IL-1beta
29	C05	Ghrelin	77	G05	IL-1beta
30	C06	Ghrelin	78	G06	IL-1beta
31	C07	Leptin	79	G07	IL-2
32	C08	Leptin	80	G08	IL-2
33	C09	Leptin	81	G09	IL-2
34	C10	Leptin	82	G10	IL-2
35	C11	Leptin	83	G11	IL-2
36	C12	Leptin	84	G12	IL-2
37	D01	Gastrin	85	H01	IL-6
38	D02	Gastrin	86	H02	IL-6
39	D03	Gastrin	87	H03	IL-6
40	D04	Gastrin	88	H04	IL-6
41	D05	Gastrin	89	H05	IL-6
42	D06	Gastrin	90	H06	IL-6
43	D07	Exendin-3	91	H07	IL-8
44	D08	Exendin-3	92	H08	IL-8
45	D09	Exendin-3	93	H09	IL-8
46	D10	Exendin-3	94	H10	IL-8
47	D11	Exendin-3	95	H11	IL-8
48	D12	Exendin-3	96	H12	IL-8

PMM1_Post-C-19 Control to Pre-C-19 Control

Well	Well compound	Mean Pre-C-19 Control	Mean Post-C-19 Control	Unadjusted p-value	Adjusted p-value	Fold difference in NADH production
B08	a-Methyl-D-Glucoside	1.85657	4.40779	1e-05	0.00E+00	2.374
A10	D-Trehalose	1.56219	3.79791	0.00E+00	1.00E-05	2.431
B01	D-Glucose-6-Phosphate	1.60757	3.84196	0.00E+00	1.00E-05	2.390
B03	L-Glucose	1.51636	3.49122	0.00E+00	1.00E-05	2.302
B04	D-(+)-Glucose	1.72926	8.122	0.00E+00	1.00E-05	4.697
B09	b-Methyl-D-Glucoside	1.12641	4.17413	0.00E+00	1.00E-05	3.706
B10	Salicin	1.57695	4.41177	0.00E+00	1.00E-05	2.798
B12	N-Acetyl-D-Glucosamine	1.11007	3.5498	0.00E+00	1.00E-05	3.198
C01	D-GlucosaminicAcid	1.58488	5.53291	0.00E+00	1.00E-05	3.491
C07	D-Mannitol	1.58322	4.20894	0.00E+00	1.00E-05	2.658
C12	Turanose	1.48064	4.01875	0.00E+00	1.00E-05	2.714
D03	L-Rhamnose	1.4896	4.60654	0.00E+00	1.00E-05	3.092
D09	D-Raffinose	1.26062	4.3523	0.00E+00	1.00E-05	3.453
D12	a-D-Lactose	1.21043	3.14869	0.00E+00	1.00E-05	2.601
E01	MelibionicAcid	1.58374	3.76591	0.00E+00	1.00E-05	2.378
E02	D-Melibiose	2.13685	4.70141	0.00E+00	1.00E-05	2.200
E04	a-Methyl-D-Galactoside	1.58663	4.69429	0.00E+00	1.00E-05	2.959
E06	n-acetyl-neuraminicacid	1.57827	4.41423	0.00E+00	1.00E-05	2.797
E12	Inosine	2.4077	5.63882	0.00E+00	1.00E-05	2.342
F03	D-Arabinose	1.5118	3.64755	0.00E+00	1.00E-05	2.413
F06	m-Inositol	1.52132	4.4791	0.00E+00	1.00E-05	2.944
F10	D,L-a-GlycerolPhosphate	1.58118	4.65106	0.00E+00	1.00E-05	2.942
F12	CitricAcid	1.39962	3.60933	0.00E+00	1.00E-05	2.579
G03	MethylD-Lactate	1.40616	3.5912	0.00E+00	1.00E-05	2.554
G04	MethylPyruvate	1.58485	5.23809	0.00E+00	1.00E-05	3.305
G07	SuccinamicAcid	1.59495	5.4922	0.00E+00	1.00E-05	3.443
G10	L-MalicAcid	1.58019	4.99331	0.00E+00	1.00E-05	3.160
H03	a-Keto-ButyricAcid	1.03195	3.00024	0.00E+00	1.00E-05	2.907
H04	D,L-a-Hydroxy-ButyricAcid	1.59071	3.54861	0.00E+00	1.00E-05	2.231
H06	g-Hydroxy-ButyricAcid	1.62256	3.24812	0.00E+00	1.00E-05	2.002
H09	3-Hydroxy2-Butanone	1.46315	3.40786	0.00E+00	1.00E-05	2.329
H12	HexanoicAcid	1.13598	3.22896	0.00E+00	1.00E-05	2.842
B05	D-(+)-Glucose	10.36269	4.73415	1e-05	1.00E-05	0.457
B11	D-Sorbitol	1.64605	4.02738	1e-05	1.00E-05	2.447
C05	D-Mannose	10.28163	4.778	1e-05	1.00E-05	0.465
E07	Pectin	1.56693	3.96491	1e-05	1.00E-05	2.530
E09	Thymidine	0.96323	2.31526	1e-05	1.00E-05	2.404
G01	TricarballylicAcid	1.58466	3.34869	1e-05	1.00E-05	2.113
G12	m-TartaricAcid	1.11971	3.25455	1e-05	1.00E-05	2.907
H01	AcetoaceticAcid	1.58353	2.81342	1e-05	1.00E-05	1.777
A04	a-Cyclodextrin	1.59033	3.22404	1e-05	1.00E-05	2.027
B07	3-MethylGlucose	1.58448	3.07868	1e-05	1.00E-05	1.943
E08	Sedoheptulosan	2.11955	4.47795	2e-05	1.00E-05	2.113
F08	1,2-Propanediol	2.10624	4.25053	2e-05	1.00E-05	2.018
G09	MonoMethylSuccinate	2.17271	4.43058	2e-05	1.00E-05	2.039
B02	D-Glucose-1-Phosphate	3.95925	6.93162	3e-05	1.00E-05	1.751
A07	Maltitol	1.58443	3.30438	3e-05	1.00E-05	2.086
A01	NegativeControl	1.58551	2.88492	4.00E-05	1.00E-05	1.820
D07	D-Fructose	1.66904	2.72179	4e-05	1.00E-05	1.631
D10	Lactitol	1.57803	3.13076	5e-05	1.00E-05	1.984
C10	Sucrose	1.57505	3.06044	5e-05	1.00E-05	1.943
F04	b-Methyl-D-Xyloside	1.58318	3.0346	5e-05	1.00E-05	1.917
D11	Lactulose	1.87232	3.53636	8e-05	5.00E-05	1.889
G11	D-MalicAcid	1.74859	3.49274	1.20E-04	1.50E-04	1.997
A05	Dextrin	7.61495	2.48184	1.70E-04	2.20E-04	0.326
A02	NegativeControl	2.20522	3.96209	1.70E-04	2.90E-04	1.797

H02	g-AminoButyricAcid	2.30666	3.92155	2.90E-04	2.90E-04	1.700
A12	Gentiobiose	1.19621	2.53234	3.40E-04	5.60E-04	2.117
G08	SuccinicAcid	2.39827	4.53644	3.40E-04	5.60E-04	1.892
E11	Adenosine	2.21986	4.27884	4.60E-04	7.40E-04	1.928
C04	Mannan	1.59352	3.31283	5e-04	7.90E-04	2.079
E10	Uridine	1.58002	2.93783	6.70E-04	1.04E-03	1.859
G05	PyruvicAcid	1.88115	2.92693	8.30E-04	1.25E-03	1.556
H05	b-Hydroxy-ButyricAcid	2.5496	4.09856	8.30E-04	1.25E-03	1.608
A11	D-Cellobiose	1.72578	3.44325	9.60E-04	1.41E-03	1.995
D01	D-Tagatose	1.59661	2.09824	1.03E-03	1.47E-03	1.314
E05	b-Methyl-D-Galactoside	2.34254	3.87863	1.03E-03	1.47E-03	1.656
A03	NegativeControl	1.59126	2.64097	1.06E-03	1.50E-03	1.660
C09	D-Melezitose	1.22451	2.35218	1.10E-03	1.53E-03	1.921
F01	Adonitol	1.58473	3.18128	2.18E-03	2.99E-03	2.007
C11	Palatinose	2.20497	3.02439	2.49E-03	3.36E-03	1.372
F09	2-Aminoethanol	1.29311	1.88127	2.83E-03	3.78E-03	1.455
D08	Stachyose	2.09888	3.27371	1.68E-02	2.22E-02	1.560
H10	PropionicAcid	1.57642	2.1573	3.14E-02	4.07E-02	1.368
D06	D-Fructose-6-Phosphate	2.73262	3.59776	3.30E-02	4.22E-02	1.317
D04	L-Fucose	1.5848	2.27723	3.47E-02	4.38E-02	1.437
G02	L-LacticAcid(DL)	3.18956	4.42419	4.20E-02	5.24E-02	1.387
F02	L-Arabinose	2.30891	3.1432	5.31E-02	6.54E-02	1.361
A06	Glycogen	4.73697	3.8674	7.26E-02	8.83E-02	0.816
H07	ButyricAcid	1.55827	1.77859	8.62E-02	1.03E-01	1.141
A08	Maltotriose	4.85219	3.16627	1.02E-01	1.21E-01	0.653
A09	Maltose	3.18192	4.34863	1.10E-01	1.29E-01	1.367
C03	ChondroitinSulfateC	1.49428	2.31256	1.19E-01	1.38E-01	1.548
F07	i-Erythritol	1.57845	2.16029	1.34E-01	1.52E-01	1.369
G06	a-Keto-GlutaricAcid	2.64561	2.47503	1.34E-01	1.52E-01	0.936
H08	2,3-Butanediol	2.39019	3.16439	1.39E-01	1.56E-01	1.324
C08	N-Acetyl-D-Mannosamine	2.03632	2.91362	1.45E-01	1.60E-01	1.431
B06	D-(+)-Glucose	6.50388	7.52753	2.62E-01	2.86E-01	1.157
E03	D-Galactose	3.00931	3.77191	3.26E-01	3.52E-01	1.253
F05	Xylitol	2.82566	3.59424	3.46E-01	3.69E-01	1.272
D02	L-Sorbose	2.33868	2.23731	3.77E-01	3.98E-01	0.957
C06	a-Methyl-D-Mannoside	1.71951	2.19216	4.10E-01	4.28E-01	1.275
F11	Glycerol	1.97046	2.56248	4.81E-01	4.97E-01	1.300
C02	D-GlucuronicAcid	2.46903	2.444	6.99E-01	7.14E-01	0.990
H11	AceticAcid	2.22383	2.64894	9.45E-01	9.55E-01	1.191
D05	D-Fucose	2.38144	2.78772	9.60E-01	9.60E-01	1.171

PMM1_Post-C-19 Control to LC-19

Well	Well Compound	Mean Post-C-19 Control	Mean LC-19	Unadjusted p-value	Adjusted p-value	Fold difference in NADH production
A01	NegativeControl	2.88492	1.77126	2.09E-03	1.60E-01	0.614
A02	NegativeControl	3.96209	2.59192	1.47E-02	1.60E-01	0.654
A03	NegativeControl	2.64097	1.67861	6.84E-03	1.60E-01	0.636
A04	a-Cyclodextrin	3.22404	1.95511	6.84E-03	1.60E-01	0.606
A06	Glycogen	3.8674	2.68037	2.88E-02	1.60E-01	0.693
A07	Maltitol	3.30438	1.8428	1.47E-02	1.60E-01	0.558
A08	Maltotriose	3.16627	2.20141	5.38E-02	1.60E-01	0.695
A09	Maltose	4.34863	3.10467	6.30E-02	1.60E-01	0.714
A10	D-Trehalose	3.79791	2.4438	6.30E-02	1.60E-01	0.643
A11	D-Cellobiose	3.44325	1.98125	2.32E-02	1.60E-01	0.575
A12	Gentiobiose	2.53234	1.64788	4.33E-02	1.60E-01	0.651
B01	D-Glucose-6-Phosphate	3.84196	2.96379	3.55E-02	1.60E-01	0.771
B03	L-Glucose	3.49122	2.74921	4.33E-02	1.60E-01	0.787
B06	D-(+)-Glucose	7.52753	5.94091	8.92E-02	1.60E-01	0.789
B08	a-Methyl-D-Glucoside	4.40779	3.49641	1.05E-01	1.60E-01	0.793
B11	D-Sorbitol	4.02738	3.09851	4.33E-02	1.60E-01	0.769
B12	N-Acetyl-D-Glucosamine	3.5498	2.66852	2.88E-02	1.60E-01	0.752
C01	D-GlucosaminicAcid	5.53291	4.41218	7.53E-02	1.60E-01	0.797
C06	a-Methyl-D-Mannoside	2.19216	1.74863	1.05E-01	1.60E-01	0.798
C07	D-Mannitol	4.20894	3.25998	1.05E-01	1.60E-01	0.775
C09	D-Melezitose	2.35218	1.80516	6.30E-02	1.60E-01	0.767
C10	Sucrose	3.06044	2.38016	1.05E-01	1.60E-01	0.778
C11	Palatinose	3.02439	2.64368	6.30E-02	1.60E-01	0.874
D01	D-Tagatose	2.09824	1.83373	4.33E-02	1.60E-01	0.874
D02	L-Sorbose	2.23731	1.98336	1.05E-01	1.60E-01	0.886
D04	L-Fucose	2.27723	1.84668	7.53E-02	1.60E-01	0.811
D05	D-Fucose	2.78772	2.23762	8.92E-02	1.60E-01	0.803
D07	D-Fructose	2.72179	2.19758	1.05E-01	1.60E-01	0.807
D08	Stachyose	3.27371	2.66399	7.53E-02	1.60E-01	0.814
D09	D-Raffinose	4.3523	3.51434	4.33E-02	1.60E-01	0.807
D10	Lactitol	3.13076	2.47767	8.92E-02	1.60E-01	0.791
D11	Lactulose	3.53636	2.85652	5.24E-02	1.60E-01	0.808
E02	D-Melibiose	4.70141	3.64624	8.92E-02	1.60E-01	0.776
E04	a-Methyl-D-Galactoside	4.69429	3.85582	7.53E-02	1.60E-01	0.821
E05	b-Methyl-D-Galactoside	3.87863	3.0225	8.92E-02	1.60E-01	0.779
E06	n-acetyl-neuraminicacid	4.41423	3.36981	6.30E-02	1.60E-01	0.763
E07	Pectin	3.96491	3.09101	8.92E-02	1.60E-01	0.780
E09	Thymidine	2.31526	1.81888	8.92E-02	1.60E-01	0.786
F01	Adonitol	3.18128	2.39749	8.92E-02	1.60E-01	0.754
F02	L-Arabinose	3.1432	2.57401	1.05E-01	1.60E-01	0.819
F03	D-Arabinose	3.64755	3.17261	1.05E-01	1.60E-01	0.870
F04	b-Methyl-D-Xyloside	3.0346	2.45596	5.24E-02	1.60E-01	0.809
F05	Xylitol	3.59424	2.87647	8.92E-02	1.60E-01	0.800
F06	m-Inositol	4.4791	3.47102	7.53E-02	1.60E-01	0.775
F07	i-Erythritol	2.16029	1.7571	8.92E-02	1.60E-01	0.813
F11	Glycerol	2.56248	2.01592	7.53E-02	1.60E-01	0.787
F12	CitricAcid	3.60933	2.74696	6.30E-02	1.60E-01	0.761
G01	TricarballylicAcid	3.34869	2.41684	5.24E-02	1.60E-01	0.722
G04	MethylPyruvate	5.23809	4.07872	5.24E-02	1.60E-01	0.779
G05	PyruvicAcid	2.92693	2.40562	4.33E-02	1.60E-01	0.822
G06	a-Keto-GlutaricAcid	2.47503	1.95228	6.30E-02	1.60E-01	0.789
G07	SuccinamicAcid	5.4922	4.0255	2.88E-02	1.60E-01	0.733
G09	MonoMethylSuccinate	4.43058	3.18521	4.33E-02	1.60E-01	0.719
G10	L-MalicAcid	4.99331	3.54324	4.33E-02	1.60E-01	0.710
G11	D-MalicAcid	3.49274	2.5208	3.55E-02	1.60E-01	0.722
H01	AcetoaceticAcid	2.81342	2.37099	7.53E-02	1.60E-01	0.843

H02	g-AminoButyricAcid	3.92155	3.20663	1.05E-01	1.60E-01	0.818
H04	D,L-a-Hydroxy-ButyricAcid	3.54861	2.79082	1.05E-01	1.60E-01	0.786
H05	b-Hydroxy-ButyricAcid	4.09856	3.38874	1.05E-01	1.60E-01	0.827
H08	2,3-Butanediol	3.16439	2.53145	6.30E-02	1.60E-01	0.800
H09	3-Hydroxy2-Butanone	3.40786	2.62419	6.30E-02	1.60E-01	0.770
H10	PropionicAcid	2.1573	1.68566	6.30E-02	1.60E-01	0.781
H12	HexanoicAcid	3.22896	2.33741	2.88E-02	1.60E-01	0.724
B02	D-Glucose-1-Phosphate	6.93162	5.32532	1.23E-01	1.74E-01	0.768
C04	Mannan	3.31283	2.58017	1.23E-01	1.74E-01	0.779
G02	L-LacticAcid(DL)	4.42419	3.35572	1.23E-01	1.74E-01	0.758
G03	MethylD-Lactate	3.5912	2.80973	1.23E-01	1.74E-01	0.782
H06	g-Hydroxy-ButyricAcid	3.24812	2.50465	1.23E-01	1.74E-01	0.771
C08	N-Acetyl-D-Mannosamine	2.91362	2.45829	1.30E-01	1.81E-01	0.844
B09	b-Methyl-D-Glucoside	4.17413	3.33936	1.43E-01	1.86E-01	0.800
D12	a-D-Lactose	3.14869	2.59668	1.43E-01	1.86E-01	0.825
E01	MelibionicAcid	3.76591	2.94364	1.43E-01	1.86E-01	0.782
E08	Sedoheptulosan	4.47795	3.56301	1.43E-01	1.86E-01	0.796
H11	AceticAcid	2.64894	2.06688	1.43E-01	1.86E-01	0.780
A05	Dextrin	2.48184	1.65973	1.65E-01	1.91E-01	0.669
B05	D-(+)-Glucose	4.73415	3.95513	1.65E-01	1.91E-01	0.835
B07	3-MethylGlucose	3.07868	2.51143	1.65E-01	1.91E-01	0.816
D03	L-Rhamnose	4.60654	3.70717	1.65E-01	1.91E-01	0.805
E03	D-Galactose	3.77191	2.96499	1.65E-01	1.91E-01	0.786
F09	2-Aminoethanol	1.88127	1.5526	1.65E-01	1.91E-01	0.825
G08	SuccinicAcid	4.53644	3.67498	1.65E-01	1.91E-01	0.810
H03	a-Keto-ButyricAcid	3.00024	2.83295	1.65E-01	1.91E-01	0.944
H07	ButyricAcid	1.77859	1.54957	1.65E-01	1.91E-01	0.871
B10	Salicin	4.41177	3.7011	1.90E-01	2.15E-01	0.839
E12	Inosine	5.63882	4.87627	1.90E-01	2.15E-01	0.865
C03	ChondroitinSulfateC	2.31256	1.72861	2.18E-01	2.37E-01	0.747
D06	D-Fructose-6-Phosphate	3.59776	2.95269	2.18E-01	2.37E-01	0.821
F08	1,2-Propanediol	4.25053	3.47883	2.18E-01	2.37E-01	0.818
E10	Uridine	2.93783	2.30034	2.47E-01	2.67E-01	0.783
C12	Turanose	4.01875	3.4135	3.15E-01	3.36E-01	0.849
C05	D-Mannose	4.778	4.14162	3.45E-01	3.63E-01	0.867
B04	D-(+)-Glucose	8.122	7.1184	3.53E-01	3.68E-01	0.876
C02	D-GlucuronicAcid	2.444	2.11766	3.93E-01	4.01E-01	0.866
G12	m-TartaricAcid	3.25455	2.56954	3.93E-01	4.01E-01	0.790
E11	Adenosine	4.27884	3.53657	4.81E-01	4.86E-01	0.827
F10	D,L-a-GlycerolPhosphate	4.65106	4.59679	9.71E-01	9.71E-01	0.988

PMM1_Post-C-19 Control to LC-19 more than 4 symptoms

Well	Well compound	Mean Post-C-19 Control	Mean LC-19 >4 Symptoms	Unadjusted p-value	Adjusted p-value	Fold difference in NADH production
A01	NegativeControl	2.88492	1.07936	2.50E-04	1.20E-02	0.374
H10	PropionicAcid	2.1573	1.15552	2.50E-04	1.20E-02	0.536
G05	PyruvicAcid	2.92693	1.74442	1.00E-03	1.60E-02	0.596
G07	SuccinamicAcid	5.4922	3.03578	1.00E-03	1.60E-02	0.553
H07	ButyricAcid	1.77859	1.15941	1.00E-03	1.60E-02	0.652
H12	HexanoicAcid	3.22896	1.81057	1.00E-03	1.60E-02	0.561
A03	NegativeControl	2.64097	1.48716	7.49E-03	2.64E-02	0.563
A04	a-Cyclodextrin	3.22404	1.82226	1.10E-02	2.64E-02	0.565
B01	D-Glucose-6-Phosphate	3.84196	2.47561	7.49E-03	2.64E-02	0.644
B03	L-Glucose	3.49122	2.08755	1.10E-02	2.64E-02	0.598
B12	N-Acetyl-D-Glucosamine	3.5498	2.10016	1.10E-02	2.64E-02	0.592
C01	D-GlucosaminicAcid	5.53291	3.15243	1.10E-02	2.64E-02	0.570
C11	Palatinose	3.02439	2.22019	7.49E-03	2.64E-02	0.734
D01	D-Tagatose	2.09824	1.60321	4.75E-03	2.64E-02	0.764
D04	L-Fucose	2.27723	1.35776	1.10E-02	2.64E-02	0.596
D07	D-Fructose	2.72179	1.83029	1.10E-02	2.64E-02	0.672
D11	Lactulose	3.53636	2.02835	7.49E-03	2.64E-02	0.574
E04	a-Methyl-D-Galactoside	4.69429	2.66673	1.10E-02	2.64E-02	0.568
E05	b-Methyl-D-Galactoside	3.87863	2.16942	1.10E-02	2.64E-02	0.559
E06	n-acetyl-neuraminicacid	4.41423	2.37292	1.10E-02	2.64E-02	0.538
E07	Pectin	3.96491	2.17507	1.10E-02	2.64E-02	0.549
F03	D-Arabinose	3.64755	2.25992	1.10E-02	2.64E-02	0.620
F04	b-Methyl-D-Xyloside	3.0346	1.6901	7.49E-03	2.64E-02	0.557
F06	m-Inositol	4.4791	2.51609	1.10E-02	2.64E-02	0.562
F11	Glycerol	2.56248	1.43552	1.10E-02	2.64E-02	0.560
F12	CitricAcid	3.60933	2.00251	1.10E-02	2.64E-02	0.555
G04	MethylPyruvate	5.23809	2.92752	7.49E-03	2.64E-02	0.559
G06	a-Keto-GlutaricAcid	2.47503	1.55595	4.75E-03	2.64E-02	0.629
G09	MonoMethylSuccinate	4.43058	2.50883	4.75E-03	2.64E-02	0.566
G10	L-MalicAcid	4.99331	2.70115	7.49E-03	2.64E-02	0.541
G11	D-MalicAcid	3.49274	1.81583	7.49E-03	2.64E-02	0.520
H01	AcetoaceticAcid	2.81342	1.82843	3.00E-03	2.64E-02	0.650
H02	g-AminoButyricAcid	3.92155	2.28017	7.49E-03	2.64E-02	0.581
H03	a-Keto-ButyricAcid	3.00024	2.26245	4.75E-03	2.64E-02	0.754
H04	D,L-a-Hydroxy-ButyricAcid	3.54861	1.99261	4.75E-03	2.64E-02	0.562
H05	b-Hydroxy-ButyricAcid	4.09856	2.35721	4.75E-03	2.64E-02	0.575
H06	g-Hydroxy-ButyricAcid	3.24812	1.80595	1.10E-02	2.64E-02	0.556
H08	2,3-Butanediol	3.16439	1.79002	4.75E-03	2.64E-02	0.566
H09	3-Hydroxy2-Butanone	3.40786	1.9515	4.75E-03	2.64E-02	0.573
H11	AceticAcid	2.64894	1.4954	1.10E-02	2.64E-02	0.565
A02	NegativeControl	3.96209	2.26702	1.60E-02	2.95E-02	0.572
A11	D-Cellobiose	3.44325	1.65454	1.60E-02	2.95E-02	0.481
B11	D-Sorbitol	4.02738	2.32748	1.60E-02	2.95E-02	0.578
D02	L-Sorbose	2.23731	1.56772	1.60E-02	2.95E-02	0.701
D05	D-Fucose	2.78772	1.63317	1.60E-02	2.95E-02	0.586
D09	D-Raffinose	4.3523	2.50025	1.60E-02	2.95E-02	0.574
D10	Lactitol	3.13076	1.78426	1.60E-02	2.95E-02	0.570
E01	MelibionicAcid	3.76591	2.1181	1.60E-02	2.95E-02	0.562
F01	Adonitol	3.18128	1.49911	1.60E-02	2.95E-02	0.471
F07	i-Erythritol	2.16029	1.20345	1.60E-02	2.95E-02	0.557
F09	2-Aminoethanol	1.88127	1.29982	1.60E-02	2.95E-02	0.691
G03	MethylD-Lactate	3.5912	2.10405	1.60E-02	2.95E-02	0.586
A07	Maltitol	3.30438	1.74046	2.25E-02	3.60E-02	0.527
B08	a-Methyl-D-Glucoside	4.40779	2.60327	2.25E-02	3.60E-02	0.591
C06	a-Methyl-D-Mannoside	2.19216	1.27787	2.25E-02	3.60E-02	0.583

C07	D-Mannitol	4.20894	2.38977	2.25E-02	3.60E-02	0.568
C09	D-Melezitose	2.35218	1.3376	2.25E-02	3.60E-02	0.569
D08	Stachyose	3.27371	1.88096	2.25E-02	3.60E-02	0.575
E02	D-Melibiose	4.70141	2.78738	2.25E-02	3.60E-02	0.593
F05	Xylitol	3.59424	2.01447	2.25E-02	3.60E-02	0.560
A12	Gentiobiose	2.53234	1.47264	3.12E-02	4.34E-02	0.582
B02	D-Glucose-1-Phosphate	6.93162	4.37329	3.12E-02	4.34E-02	0.631
C04	Mannan	3.31283	1.82016	3.12E-02	4.34E-02	0.549
C10	Sucrose	3.06044	1.7705	3.12E-02	4.34E-02	0.579
D03	L-Rhamnose	4.60654	2.72046	3.12E-02	4.34E-02	0.591
D12	a-D-Lactose	3.14869	1.86169	3.12E-02	4.34E-02	0.591
E08	Sedoheptulosan	4.47795	2.61571	3.12E-02	4.34E-02	0.584
G01	TricarballylicAcid	3.34869	2.04212	3.12E-02	4.34E-02	0.610
G02	L-LacticAcid(DL)	4.42419	2.68664	3.12E-02	4.34E-02	0.607
C08	N-Acetyl-D-Mannosamine	2.91362	1.75975	3.92E-02	5.37E-02	0.604
D06	D-Fructose-6-Phosphate	3.59776	2.29237	4.20E-02	5.44E-02	0.637
E03	D-Galactose	3.77191	2.19292	4.20E-02	5.44E-02	0.581
E09	Thymidine	2.31526	1.439	4.20E-02	5.44E-02	0.622
G08	SuccinicAcid	4.53644	2.7597	4.20E-02	5.44E-02	0.608
B06	D-(+)-Glucose	7.52753	5.47193	5.59E-02	7.07E-02	0.727
C12	Turanose	4.01875	2.7691	5.59E-02	7.07E-02	0.689
B07	3-MethylGlucose	3.07868	2.02831	7.27E-02	8.61E-02	0.659
B09	b-Methyl-D-Glucoside	4.17413	2.59848	7.27E-02	8.61E-02	0.623
C02	D-GlucuronicAcid	2.444	1.70616	7.27E-02	8.61E-02	0.698
C03	ChondroitinSulfateC	2.31256	1.2646	7.27E-02	8.61E-02	0.547
F02	L-Arabinose	3.1432	2.09101	7.27E-02	8.61E-02	0.665
A10	D-Trehalose	3.79791	2.27782	9.34E-02	1.05E-01	0.600
B10	Salicin	4.41177	2.89573	9.34E-02	1.05E-01	0.656
E10	Uridine	2.93783	1.7684	9.34E-02	1.05E-01	0.602
F08	1,2-Propanediol	4.25053	2.63018	9.34E-02	1.05E-01	0.619
A06	Glycogen	3.8674	2.83133	1.18E-01	1.32E-01	0.732
C05	D-Mannose	4.778	3.66289	1.47E-01	1.60E-01	0.767
G12	m-TartaricAcid	3.25455	1.99442	1.47E-01	1.60E-01	0.613
A08	Maltotriose	3.16627	2.285	1.75E-01	1.89E-01	0.722
B05	D-(+)-Glucose	4.73415	3.70872	1.81E-01	1.90E-01	0.783
E12	Inosine	5.63882	4.51102	1.81E-01	1.90E-01	0.800
A05	Dextrin	2.48184	1.66203	2.20E-01	2.27E-01	0.670
B04	D-(+)-Glucose	8.122	6.57172	2.20E-01	2.27E-01	0.809
A09	Maltose	4.34863	3.26281	2.63E-01	2.69E-01	0.750
E11	Adenosine	4.27884	3.28606	4.28E-01	4.32E-01	0.768
F10	D,L-a-GlycerolPhosphate	4.65106	3.92922	5.62E-01	5.62E-01	0.845

PMM1_Post-C-19 Control to LC-19 Official Diagnosis

Well	Well compound	Mean Post-C-19 Control	Mean LC-19 official Diagnosis	Unadjusted p-value	Adjusted p-value	Fold difference in NADH production
A01	NegativeControl	2.885	1.104	6.99E-03	4.48E-02	0.383
A02	NegativeControl	3.962	1.911	6.99E-03	4.48E-02	0.482
A03	NegativeControl	2.641	1.225	6.99E-03	4.48E-02	0.464
A04	a-Cyclodextrin	3.224	1.522	6.99E-03	4.48E-02	0.472
A07	Maltitol	3.304	1.345	6.99E-03	4.48E-02	0.407
A10	D-Trehalose	3.798	1.862	2.80E-02	4.48E-02	0.490
A11	D-Cellobiose	3.443	1.618	1.40E-02	4.48E-02	0.470
A12	Gentiobiose	2.532	1.252	2.80E-02	4.48E-02	0.494
B02	D-Glucose-1-Phosphate	6.932	3.403	2.80E-02	4.48E-02	0.491
B03	L-Glucose	3.491	1.816	1.40E-02	4.48E-02	0.520
B07	3-MethylGlucose	3.079	1.707	1.40E-02	4.48E-02	0.555
B08	a-Methyl-D-Glucoside	4.408	2.228	2.80E-02	4.48E-02	0.505
B09	b-Methyl-D-Glucoside	4.174	2.112	2.80E-02	4.48E-02	0.506
B10	Salicin	4.412	2.314	6.99E-03	4.48E-02	0.525
B11	D-Sorbitol	4.027	2.006	1.40E-02	4.48E-02	0.498
B12	N-Acetyl-D-Glucosamine	3.550	1.771	6.99E-03	4.48E-02	0.499
C01	D-GlucosaminicAcid	5.533	2.871	2.80E-02	4.48E-02	0.519
C06	a-Methyl-D-Mannoside	2.192	1.123	2.80E-02	4.48E-02	0.512
C07	D-Mannitol	4.209	2.163	2.80E-02	4.48E-02	0.514
C08	N-Acetyl-D-Mannosamine	2.914	1.549	2.80E-02	4.48E-02	0.532
C09	D-Melezitose	2.352	1.197	2.80E-02	4.48E-02	0.509
C10	Sucrose	3.060	1.609	2.80E-02	4.48E-02	0.526
C11	Palatinose	3.024	2.153	2.80E-02	4.48E-02	0.712
C12	Turanose	4.019	2.422	2.80E-02	4.48E-02	0.603
D01	D-Tagatose	2.098	1.569	2.80E-02	4.48E-02	0.748
D04	L-Fucose	2.277	1.254	1.40E-02	4.48E-02	0.551
D05	D-Fucose	2.788	1.456	2.80E-02	4.48E-02	0.522
D09	D-Raffinose	4.352	2.321	2.80E-02	4.48E-02	0.533
D10	Lactitol	3.131	1.603	2.80E-02	4.48E-02	0.512
D11	Lactulose	3.536	1.904	2.80E-02	4.48E-02	0.538
D12	a-D-Lactose	3.149	1.669	2.80E-02	4.48E-02	0.530
E01	MelibionicAcid	3.766	1.973	2.80E-02	4.48E-02	0.524
E03	D-Galactose	3.772	1.897	2.80E-02	4.48E-02	0.503
E04	a-Methyl-D-Galactoside	4.694	2.355	1.40E-02	4.48E-02	0.502
E05	b-Methyl-D-Galactoside	3.879	1.981	2.80E-02	4.48E-02	0.511
E06	n-acetyl-neuraminicacid	4.414	2.207	2.80E-02	4.48E-02	0.500
E07	Pectin	3.965	1.988	2.80E-02	4.48E-02	0.501
F03	D-Arabinose	3.648	2.166	2.80E-02	4.48E-02	0.594
F04	b-Methyl-D-Xyloside	3.035	1.537	2.80E-02	4.48E-02	0.506
F05	Xylitol	3.594	1.839	2.80E-02	4.48E-02	0.512
F06	m-Inositol	4.479	2.328	2.80E-02	4.48E-02	0.520
F07	i-Erythritol	2.160	1.121	2.80E-02	4.48E-02	0.519
F11	Glycerol	2.562	1.291	1.40E-02	4.48E-02	0.504
G03	MethylD-Lactate	3.591	1.858	2.80E-02	4.48E-02	0.517
G04	MethylPyruvate	5.238	2.680	1.40E-02	4.48E-02	0.512
G05	PyruvicAcid	2.927	1.671	6.99E-03	4.48E-02	0.571
G06	a-Keto-GlutaricAcid	2.475	1.462	1.40E-02	4.48E-02	0.591
G07	SuccinamicAcid	5.492	2.893	6.99E-03	4.48E-02	0.527
G09	MonoMethylSuccinate	4.431	2.320	6.99E-03	4.48E-02	0.524
G10	L-MalicAcid	4.993	2.594	2.80E-02	4.48E-02	0.520
G11	D-MalicAcid	3.493	1.713	2.80E-02	4.48E-02	0.490
H01	AcetoaceticAcid	2.813	1.823	2.80E-02	4.48E-02	0.648
H02	g-AminoButyricAcid	3.922	2.209	2.80E-02	4.48E-02	0.563
H04	D,L-a-Hydroxy-ButyricAcid	3.549	1.890	1.40E-02	4.48E-02	0.533
H05	b-Hydroxy-ButyricAcid	4.099	2.229	1.40E-02	4.48E-02	0.544
H07	ButyricAcid	1.779	1.261	2.80E-02	4.48E-02	0.709
H08	2,3-Butanediol	3.164	1.687	1.40E-02	4.48E-02	0.533
H09	3-Hydroxy2-Butanone	3.408	1.850	1.40E-02	4.48E-02	0.543
H10	PropionicAcid	2.157	1.141	6.99E-03	4.48E-02	0.529
H12	HexanoicAcid	3.229	1.737	6.99E-03	4.48E-02	0.538

B01	D-Glucose-6-Phosphate	3.842	2.482	4.90E-02	6.53E-02	0.646
C03	ChondroitinSulfateC	2.313	1.094	4.90E-02	6.53E-02	0.473
D03	L-Rhamnose	4.607	2.426	4.90E-02	6.53E-02	0.527
D08	Stachyose	3.274	1.761	4.90E-02	6.53E-02	0.538
E08	Sedoheptulosan	4.478	2.419	4.90E-02	6.53E-02	0.540
E09	Thymidine	2.315	1.269	4.90E-02	6.53E-02	0.548
F01	Adonitol	3.181	1.402	4.90E-02	6.53E-02	0.441
F12	CitricAcid	3.609	1.954	4.90E-02	6.53E-02	0.541
G02	L-LacticAcid(DL)	4.424	2.535	4.90E-02	6.53E-02	0.573
H03	a-Keto-ButyricAcid	3.000	2.359	4.90E-02	6.53E-02	0.786
H06	g-Hydroxy-ButyricAcid	3.248	1.775	4.90E-02	6.53E-02	0.546
H11	AceticAcid	2.649	1.456	4.90E-02	6.53E-02	0.550
A06	Glycogen	3.867	2.322	7.69E-02	9.35E-02	0.600
C04	Mannan	3.313	1.639	7.69E-02	9.35E-02	0.495
D02	L-Sorbose	2.237	1.549	7.69E-02	9.35E-02	0.692
D06	D-Fructose-6-Phosphate	3.598	2.228	7.69E-02	9.35E-02	0.619
E02	D-Melibiose	4.701	2.730	7.69E-02	9.35E-02	0.581
F09	2-Aminoethanol	1.881	1.340	7.69E-02	9.35E-02	0.712
G08	SuccinicAcid	4.536	2.590	7.69E-02	9.35E-02	0.571
B05	D-(+)-Glucose	4.734	3.261	1.12E-01	1.28E-01	0.689
D07	D-Fructose	2.722	1.802	1.12E-01	1.28E-01	0.662
E10	Uridine	2.938	1.610	1.12E-01	1.28E-01	0.548
E12	Inosine	5.639	4.223	1.12E-01	1.28E-01	0.749
G01	TricarballylicAcid	3.349	1.996	1.12E-01	1.28E-01	0.596
A08	Maltotriose	3.166	1.925	1.50E-01	1.70E-01	0.608
B06	D-(+)-Glucose	7.528	5.232	1.61E-01	1.75E-01	0.695
F08	1,2-Propanediol	4.251	2.522	1.61E-01	1.75E-01	0.593
G12	m-TartaricAcid	3.255	1.830	1.61E-01	1.75E-01	0.562
A09	Maltose	4.349	2.757	2.17E-01	2.29E-01	0.634
B04	D-(+)-Glucose	8.122	5.970	2.17E-01	2.29E-01	0.735
C05	D-Mannose	4.778	3.405	2.17E-01	2.29E-01	0.713
E11	Adenosine	4.279	3.141	2.87E-01	2.96E-01	0.734
F02	L-Arabinose	3.143	2.181	2.87E-01	2.96E-01	0.694
C02	D-GlucuronicAcid	2.444	1.809	3.71E-01	3.79E-01	0.740
A05	Dextrin	2.482	1.711	4.69E-01	4.73E-01	0.690
F10	D,L-a-GlycerolPhosphate	4.651	3.961	8.11E-01	8.11E-01	0.852

PMM1_Pre-C-19 Control to LC-19

Well	Well compound	Mean Pre-C-19 Control	Mean LC-19	Adjusted p-value	Fold difference in NADH production
B09	b-Methyl-D-Glucoside	1.12641	3.33936	1.00E-05	2.965
B10	Salicin	1.57695	3.7011	1.00E-05	2.347
C01	D-GlucosaminicAcid	1.58488	4.41218	1.00E-05	2.784
D03	L-Rhamnose	1.4896	3.70717	1.00E-05	2.489
D09	D-Raffinose	1.26062	3.51434	1.00E-05	2.788
F06	m-Inositol	1.52132	3.47102	1.00E-05	2.282
F10	D,L-a-GlycerolPhosphate	1.58118	4.59679	1.00E-05	2.907
G04	MethylPyruvate	1.58485	4.07872	1.00E-05	2.574
G07	SuccinamicAcid	1.59495	4.0255	1.00E-05	2.524
G12	m-TartaricAcid	1.11971	2.56954	1.00E-05	2.295
H03	a-Keto-ButyricAcid	1.03195	2.83295	1.00E-05	2.745
B05	D-(+)-Glucose	10.36269	3.95513	2.00E-05	0.382
C05	D-Mannose	10.28163	4.14162	2.00E-05	0.403
E04	a-Methyl-D-Galactoside	1.58663	3.85582	2.00E-05	2.430
E06	n-acetyl-neuraminicacid	1.57827	3.36981	2.00E-05	2.135
F03	D-Arabinose	1.5118	3.17261	2.00E-05	2.099
G10	L-MalicAcid	1.58019	3.54324	2.00E-05	2.242
C07	D-Mannitol	1.58322	3.25998	4.00E-05	2.059
E12	Inosine	2.4077	4.87627	5.00E-05	2.025
G03	MethylD-Lactate	1.40616	2.80973	5.00E-05	1.998
C12	Turanose	1.48064	3.4135	7.00E-05	2.305
B04	D-(+)-Glucose	1.72926	7.1184	1.00E-04	4.116
B01	D-Glucose-6-Phosphate	1.60757	2.96379	1.20E-04	1.844
D12	a-D-Lactose	1.21043	2.59668	1.20E-04	2.145
E07	Pectin	1.56693	3.09101	1.20E-04	1.973
H12	HexanoicAcid	1.13598	2.33741	1.20E-04	2.058
A05	Dextrin	7.61495	1.65973	1.50E-04	0.218
B12	N-Acetyl-D-Glucosamine	1.11007	2.66852	1.50E-04	2.404
F12	CitricAcid	1.39962	2.74696	1.50E-04	1.963
E09	Thymidine	0.96323	1.81888	1.60E-04	1.888
B03	L-Glucose	1.51636	2.74921	4.70E-04	1.813
H09	3-Hydroxy2-Butanone	1.46315	2.62419	4.70E-04	1.794
E01	MelibionicAcid	1.58374	2.94364	5.00E-04	1.859
H04	D,L-a-Hydroxy-ButyricAcid	1.59071	2.79082	1.13E-03	1.754
A10	D-Trehalose	1.56219	2.4438	1.37E-03	1.564
B08	a-Methyl-D-Glucoside	1.85657	3.49641	1.62E-03	1.883
G06	a-Keto-GlutaricAcid	2.64561	1.95228	1.62E-03	0.738
E02	D-Melibiose	2.13685	3.64624	2.10E-03	1.706
G01	TricarballylicAcid	1.58466	2.41684	2.36E-03	1.525
A06	Glycogen	4.73697	2.68037	2.65E-03	0.566
E08	Sedoheptulosan	2.11955	3.56301	2.77E-03	1.681
F08	1,2-Propanediol	2.10624	3.47883	3.32E-03	1.652
B11	D-Sorbitol	1.64605	3.09851	3.40E-03	1.882
E11	Adenosine	2.21986	3.53657	3.40E-03	1.593
B07	3-MethylGlucose	1.58448	2.51143	3.81E-03	1.585
H01	AcetoaceticAcid	1.58353	2.37099	3.98E-03	1.497
G08	SuccinicAcid	2.39827	3.67498	9.03E-03	1.532
C04	Mannan	1.59352	2.58017	9.41E-03	1.619
D02	L-Sorbose	2.33868	1.98336	1.89E-02	0.848
D05	D-Fucose	2.38144	2.23762	2.08E-02	0.940
H06	g-Hydroxy-ButyricAcid	1.62256	2.50465	2.15E-02	1.544
D10	Lactitol	1.57803	2.47767	2.23E-02	1.570
D07	D-Fructose	1.66904	2.19758	2.32E-02	1.317
E10	Uridine	1.58002	2.30034	2.41E-02	1.456
A08	Maltotriose	4.85219	2.20141	2.50E-02	0.454
G09	MonoMethylSuccinate	2.17271	3.18521	2.59E-02	1.466

C10	Sucrose	1.57505	2.38016	2.99E-02	1.511
B02	D-Glucose-1-Phosphate	3.95925	5.32532	4.03E-02	1.345
A12	Gentiobiose	1.19621	1.64788	6.52E-02	1.378
C06	a-Methyl-D-Mannoside	1.71951	1.74863	7.98E-02	1.017
F04	b-Methyl-D-Xyloside	1.58318	2.45596	7.98E-02	1.551
C02	D-GlucuronicAcid	2.46903	2.11766	1.03E-01	0.858
F05	Xylitol	2.82566	2.87647	1.06E-01	1.018
A04	a-Cyclodextrin	1.59033	1.95511	1.29E-01	1.229
F11	Glycerol	1.97046	2.01592	1.44E-01	1.023
E03	D-Galactose	3.00931	2.96499	1.54E-01	0.985
D11	Lactulose	1.87232	2.85652	1.65E-01	1.526
H11	AceticAcid	2.22383	2.06688	1.69E-01	0.929
D01	D-Tagatose	1.59661	1.83373	1.80E-01	1.149
F09	2-Aminoethanol	1.29311	1.5526	1.82E-01	1.201
H08	2,3-Butanediol	2.39019	2.53145	1.82E-01	1.059
G11	D-MalicAcid	1.74859	2.5208	1.93E-01	1.442
F07	i-Erythritol	1.57845	1.7571	1.98E-01	1.113
C09	D-Melezitose	1.22451	1.80516	2.15E-01	1.474
H02	g-AminoButyricAcid	2.30666	3.20663	2.15E-01	1.390
A07	Maltitol	1.58443	1.8428	2.20E-01	1.163
C11	Palatinose	2.20497	2.64368	2.25E-01	1.199
B06	D-(+)-Glucose	6.50388	5.94091	2.83E-01	0.913
F01	Adonitol	1.58473	2.39749	3.09E-01	1.513
G02	L-LacticAcid(DL)	3.18956	3.35572	4.15E-01	1.052
H10	PropionicAcid	1.57642	1.68566	4.35E-01	1.069
A11	D-Cellobiose	1.72578	1.98125	4.69E-01	1.148
E05	b-Methyl-D-Galactoside	2.34254	3.0225	4.69E-01	1.290
F02	L-Arabinose	2.30891	2.57401	4.69E-01	1.115
G05	PyruvicAcid	1.88115	2.40562	4.76E-01	1.279
A02	NegativeControl	2.20522	2.59192	5.65E-01	1.175
C08	N-Acetyl-D-Mannosamine	2.03632	2.45829	6.09E-01	1.207
H05	b-Hydroxy-ButyricAcid	2.5496	3.38874	6.09E-01	1.329
A03	NegativeControl	1.59126	1.67861	6.31E-01	1.055
C03	ChondroitinSulfateC	1.49428	1.72861	6.99E-01	1.157
H07	ButyricAcid	1.55827	1.54957	7.07E-01	0.994
A01	NegativeControl	1.58551	1.77126	7.14E-01	1.117
D04	L-Fucose	1.5848	1.84668	7.21E-01	1.165
D06	D-Fructose-6-Phosphate	2.73262	2.95269	7.75E-01	1.081
A09	Maltose	3.18192	3.10467	8.13E-01	0.976
D08	Stachyose	2.09888	2.66399	8.66E-01	1.269

PMM1_Pre-C-19 Control to LC-19 more than 4 symptoms

Well	Well compound	Mean Pre-C-19 Control	Mean LC-19 >4 Symptoms	Unadjusted p-value	Adjusted p-value	Fold difference in NADH production
B04	D-(+)-Glucose	1.72926	6.57172	1.40E-04	1.18E-03	3.800
B09	b-Methyl-D-Glucoside	1.12641	2.59848	1.00E-04	1.18E-03	2.307
B10	Salicin	1.57695	2.89573	1.60E-04	1.18E-03	1.836
C01	D-GlucosaminicAcid	1.58488	3.15243	1.20E-04	1.18E-03	1.989
C05	D-Mannose	10.28163	3.66289	1.60E-04	1.18E-03	0.356
D03	L-Rhamnose	1.4896	2.72046	1.30E-04	1.18E-03	1.826
D09	D-Raffinose	1.26062	2.50025	1.00E-04	1.18E-03	1.983
F06	m-Inositol	1.52132	2.51609	1.60E-04	1.18E-03	1.654
F10	D,L-a-GlycerolPhosphate	1.58118	3.92922	7.00E-05	1.18E-03	2.485
G04	MethylPyruvate	1.58485	2.92752	1.60E-04	1.18E-03	1.847
G06	a-Keto-GlutaricAcid	2.64561	1.55595	1.30E-04	1.18E-03	0.588
G07	SuccinamicAcid	1.59495	3.03578	1.20E-04	1.18E-03	1.903
H03	a-Keto-ButyricAcid	1.03195	2.26245	7.00E-05	1.18E-03	2.192
B05	D-(+)-Glucose	10.36269	3.70872	2.00E-04	1.27E-03	0.358
G12	m-TartaricAcid	1.11971	1.99442	2.00E-04	1.27E-03	1.781
E04	a-Methyl-D-Galactoside	1.58663	2.66673	3.30E-04	1.87E-03	1.681
E06	n-acetyl-neuraminicacid	1.57827	2.37292	3.30E-04	1.87E-03	1.503
B12	N-Acetyl-D-Glucosamine	1.11007	2.10016	4.10E-04	2.05E-03	1.892
F03	D-Arabinose	1.5118	2.25992	4.10E-04	2.05E-03	1.495
G10	L-MalicAcid	1.58019	2.70115	4.50E-04	2.15E-03	1.709
E12	Inosine	2.4077	4.51102	4.90E-04	2.26E-03	1.874
C07	D-Mannitol	1.58322	2.38977	6.60E-04	2.90E-03	1.509
A05	Dextrin	7.61495	1.66203	1.07E-03	4.46E-03	0.218
G03	MethylD-Lactate	1.40616	2.10405	1.17E-03	4.70E-03	1.496
C12	Turanose	1.48064	2.7691	1.41E-03	5.21E-03	1.870
D05	D-Fucose	2.38144	1.63317	1.41E-03	5.21E-03	0.686
A01	NegativeControl	1.58551	1.07936	1.55E-03	5.50E-03	0.681
D02	L-Sorbose	2.33868	1.56772	1.85E-03	6.36E-03	0.670
H12	HexanoicAcid	1.13598	1.81057	2.42E-03	8.01E-03	1.594
E07	Pectin	1.56693	2.17507	2.64E-03	8.18E-03	1.388
E09	Thymidine	0.96323	1.439	2.64E-03	8.18E-03	1.494
B01	D-Glucose-6-Phosphate	1.60757	2.47561	2.88E-03	8.64E-03	1.540
D12	a-D-Lactose	1.21043	1.86169	3.14E-03	9.13E-03	1.538
F12	CitricAcid	1.39962	2.00251	4.05E-03	1.14E-02	1.431
F05	Xylitol	2.82566	2.01447	6.11E-03	1.68E-02	0.713
A10	D-Trehalose	1.56219	2.27782	7.17E-03	1.91E-02	1.458
B03	L-Glucose	1.51636	2.08755	7.76E-03	2.01E-02	1.377
C06	a-Methyl-D-Mannoside	1.71951	1.27787	8.39E-03	2.12E-02	0.743
H08	2,3-Butanediol	2.39019	1.79002	9.80E-03	2.41E-02	0.749
H10	PropionicAcid	1.57642	1.15552	1.06E-02	2.54E-02	0.733
E03	D-Galactose	3.00931	2.19292	1.14E-02	2.67E-02	0.729
A06	Glycogen	4.73697	2.83133	1.43E-02	3.19E-02	0.598
E01	MelibionicAcid	1.58374	2.1181	1.43E-02	3.19E-02	1.337
C02	D-GlucuronicAcid	2.46903	1.70616	1.54E-02	3.21E-02	0.691
E11	Adenosine	2.21986	3.28606	1.54E-02	3.21E-02	1.480
H09	3-Hydroxy2-Butanone	1.46315	1.9515	1.54E-02	3.21E-02	1.334
F07	i-Erythritol	1.57845	1.20345	1.65E-02	3.37E-02	0.762
B08	a-Methyl-D-Glucoside	1.85657	2.60327	1.91E-02	3.81E-02	1.402
H07	ButyricAcid	1.55827	1.15941	2.05E-02	3.93E-02	0.744
H11	AceticAcid	2.22383	1.4954	2.05E-02	3.93E-02	0.672
B07	3-MethylGlucose	1.58448	2.02831	2.52E-02	4.74E-02	1.280
F11	Glycerol	1.97046	1.43552	2.89E-02	5.23E-02	0.729
H04	D,L-a-Hydroxy-ButyricAcid	1.59071	1.99261	2.89E-02	5.23E-02	1.253
B11	D-Sorbitol	1.64605	2.32748	4.27E-02	7.46E-02	1.414
G01	TricarballylicAcid	1.58466	2.04212	4.27E-02	7.46E-02	1.289
A08	Maltotriose	4.85219	2.285	4.84E-02	8.16E-02	0.471
G02	L-LacticAcid(DL)	3.18956	2.68664	4.84E-02	8.16E-02	0.842
E02	D-Melibiose	2.13685	2.78738	5.82E-02	9.64E-02	1.304
E08	Sedoheptulosan	2.11955	2.61571	6.18E-02	1.01E-01	1.234
F08	1,2-Propanediol	2.10624	2.63018	6.56E-02	1.05E-01	1.249
B06	D-(+)-Glucose	6.50388	5.47193	1.03E-01	1.60E-01	0.841

H01	AcetoaceticAcid	1.58353	1.82843	1.03E-01	1.60E-01	1.155
A12	Gentiobiose	1.19621	1.47264	1.42E-01	2.16E-01	1.231
C04	Mannan	1.59352	1.82016	1.49E-01	2.20E-01	1.142
D04	L-Fucose	1.5848	1.35776	1.49E-01	2.20E-01	0.857
A04	a-Cyclodextrin	1.59033	1.82226	1.72E-01	2.47E-01	1.146
C03	ChondroitinSulfateC	1.49428	1.2646	1.72E-01	2.47E-01	0.846
G08	SuccinicAcid	2.39827	2.7597	1.90E-01	2.68E-01	1.151
D08	Stachyose	2.09888	1.88096	2.28E-01	3.17E-01	0.896
C08	N-Acetyl-D-Mannosamine	2.03632	1.75975	2.38E-01	3.27E-01	0.864
B02	D-Glucose-1-Phosphate	3.95925	4.37329	2.60E-01	3.38E-01	1.105
D06	D-Fructose-6-Phosphate	2.73262	2.29237	2.60E-01	3.38E-01	0.839
F02	L-Arabinose	2.30891	2.09101	2.60E-01	3.38E-01	0.906
H05	b-Hydroxy-ButyricAcid	2.5496	2.35721	2.60E-01	3.38E-01	0.925
D07	D-Fructose	1.66904	1.83029	2.95E-01	3.73E-01	1.097
G09	MonoMethylSuccinate	2.17271	2.50883	2.95E-01	3.73E-01	1.155
A07	Maltitol	1.58443	1.74046	3.34E-01	4.11E-01	1.098
D10	Lactitol	1.57803	1.78426	3.34E-01	4.11E-01	1.131
C10	Sucrose	1.57505	1.7705	3.89E-01	4.67E-01	1.124
E10	Uridine	1.58002	1.7684	3.89E-01	4.67E-01	1.119
A03	NegativeControl	1.59126	1.48716	4.19E-01	4.91E-01	0.935
H06	g-Hydroxy-ButyricAcid	1.62256	1.80595	4.19E-01	4.91E-01	1.113
G05	PyruvicAcid	1.88115	1.74442	4.83E-01	5.58E-01	0.927
E05	b-Methyl-D-Galactoside	2.34254	2.16942	4.99E-01	5.71E-01	0.926
F01	Adonitol	1.58473	1.49911	5.16E-01	5.83E-01	0.946
C09	D-Melezitose	1.22451	1.3376	7.21E-01	7.95E-01	1.092
F04	b-Methyl-D-Xyloside	1.58318	1.6901	7.21E-01	7.95E-01	1.068
C11	Palatinose	2.20497	2.22019	7.61E-01	8.30E-01	1.007
A09	Maltose	3.18192	3.26281	8.01E-01	8.55E-01	1.025
D01	D-Tagatose	1.59661	1.60321	8.01E-01	8.55E-01	1.004
H02	g-AminoButyricAcid	2.30666	2.28017	8.22E-01	8.67E-01	0.989
A02	NegativeControl	2.20522	2.26702	8.63E-01	9.01E-01	1.028
G11	D-MalicAcid	1.74859	1.81583	8.84E-01	9.13E-01	1.038
A11	D-Cellobiose	1.72578	1.65454	9.26E-01	9.46E-01	0.959
D11	Lactulose	1.87232	2.02835	1.00E+00	1.00E+00	1.083
F09	2-Aminoethanol	1.29311	1.29982	1.00E+00	1.00E+00	1.005

PMM1_Pre-C-19 Control to LC-19 Official Diagnosis

Well	Well compound	Mean Pre-C-19 Control	Mean LC-19 official Diagnosis	Unadjusted p-value	Adjusted p-value	Fold difference in NADH production
B04	D-(+)-Glucose	1.729	5.970	5.92E-03	4.78E-02	3.453
B05	D-(+)-Glucose	10.363	3.261	5.26E-03	4.78E-02	0.315
B09	b-Methyl-D-Glucoside	1.126	2.112	5.92E-03	4.78E-02	1.875
C01	D-GlucosaminicAcid	1.585	2.871	5.92E-03	4.78E-02	1.811
C05	D-Mannose	10.282	3.405	5.92E-03	4.78E-02	0.331
D03	L-Rhamnose	1.490	2.426	7.47E-03	4.78E-02	1.629
D05	D-Fucose	2.381	1.456	7.47E-03	4.78E-02	0.612
D09	D-Raffinose	1.261	2.321	5.26E-03	4.78E-02	1.841
F06	m-Inositol	1.521	2.328	7.47E-03	4.78E-02	1.530
F10	D,L-a-GlycerolPhosphate	1.581	3.961	4.14E-03	4.78E-02	2.505
G04	MethylPyruvate	1.585	2.680	5.92E-03	4.78E-02	1.691
G06	a-Keto-GlutaricAcid	2.646	1.462	4.14E-03	4.78E-02	0.553
G07	SuccinamicAcid	1.595	2.893	6.66E-03	4.78E-02	1.814
G12	m-TartaricAcid	1.120	1.830	7.47E-03	4.78E-02	1.634
H03	a-Keto-ButyricAcid	1.032	2.359	4.14E-03	4.78E-02	2.286
G10	L-MalicAcid	1.580	2.594	8.37E-03	5.03E-02	1.642
B10	Salicin	1.577	2.314	9.37E-03	5.29E-02	1.467
F03	D-Arabinose	1.512	2.166	1.05E-02	5.59E-02	1.433
B01	D-Glucose-6-Phosphate	1.608	2.482	1.30E-02	5.96E-02	1.544
E04	a-Methyl-D-Galactoside	1.587	2.355	1.30E-02	5.96E-02	1.484
E06	n-acetyl-neuraminicacid	1.578	2.207	1.30E-02	5.96E-02	1.398
E03	D-Galactose	3.009	1.897	1.45E-02	6.34E-02	0.630
C06	a-Methyl-D-Mannoside	1.720	1.123	1.61E-02	6.74E-02	0.653
A01	NegativeControl	1.586	1.104	2.20E-02	6.82E-02	0.696
A05	Dextrin	7.615	1.711	1.79E-02	6.82E-02	0.225
B12	N-Acetyl-D-Glucosamine	1.110	1.771	1.99E-02	6.82E-02	1.595
C07	D-Mannitol	1.583	2.163	2.20E-02	6.82E-02	1.366
D02	L-Sorbose	2.339	1.549	2.20E-02	6.82E-02	0.662
E12	Inosine	2.408	4.223	1.99E-02	6.82E-02	1.754
F05	Xylitol	2.826	1.839	1.99E-02	6.82E-02	0.651
H08	2,3-Butanediol	2.390	1.687	2.20E-02	6.82E-02	0.706
H12	HexanoicAcid	1.136	1.737	3.27E-02	9.80E-02	1.529
F07	i-Erythritol	1.578	1.121	3.59E-02	1.05E-01	0.710
F11	Glycerol	1.970	1.291	3.95E-02	1.08E-01	0.655
F12	CitricAcid	1.400	1.954	3.95E-02	1.08E-01	1.396
A06	Glycogen	4.737	2.322	4.33E-02	1.15E-01	0.490
C12	Turanose	1.481	2.422	4.75E-02	1.20E-01	1.636
G03	MethylD-Lactate	1.406	1.858	4.75E-02	1.20E-01	1.321
D12	a-D-Lactose	1.210	1.669	5.68E-02	1.33E-01	1.379
E09	Thymidine	0.963	1.269	5.68E-02	1.33E-01	1.317
H10	PropionicAcid	1.576	1.141	5.68E-02	1.33E-01	0.724
A03	NegativeControl	1.591	1.225	6.75E-02	1.54E-01	0.770
E07	Pectin	1.567	1.988	7.35E-02	1.64E-01	1.269
C03	ChondroitinSulfateC	1.494	1.094	7.99E-02	1.74E-01	0.732
H11	AceticAcid	2.224	1.456	8.68E-02	1.85E-01	0.655
A08	Maltotriose	4.852	1.925	9.41E-02	1.96E-01	0.397
G02	L-LacticAcid(DL)	3.190	2.535	1.02E-01	2.04E-01	0.795
H09	3-Hydroxy2-Butanone	1.463	1.850	1.02E-01	2.04E-01	1.265
E01	MelibionicAcid	1.584	1.973	1.10E-01	2.12E-01	1.246
E11	Adenosine	2.220	3.141	1.10E-01	2.12E-01	1.415
D04	L-Fucose	1.585	1.254	1.28E-01	2.42E-01	0.792
C02	D-GlucuronicAcid	2.469	1.809	1.38E-01	2.51E-01	0.733
H04	D,L-a-Hydroxy-ButyricAcid	1.591	1.890	1.38E-01	2.51E-01	1.188
E02	D-Melibiose	2.137	2.730	1.60E-01	2.79E-01	1.277
H05	b-Hydroxy-ButyricAcid	2.550	2.229	1.60E-01	2.79E-01	0.874
H07	ButyricAcid	1.558	1.261	1.72E-01	2.95E-01	0.809
B03	L-Glucose	1.516	1.816	1.84E-01	3.05E-01	1.198
C08	N-Acetyl-D-Mannosamine	2.036	1.549	1.84E-01	3.05E-01	0.760
H01	AcetoaceticAcid	1.584	1.823	1.97E-01	3.21E-01	1.151
A10	D-Trehalose	1.562	1.862	2.25E-01	3.43E-01	1.192

B06	D-(+)-Glucose	6.504	5.232	2.25E-01	3.43E-01	0.804
E05	b-Methyl-D-Galactoside	2.343	1.981	2.25E-01	3.43E-01	0.846
G01	TricarballylicAcid	1.585	1.996	2.25E-01	3.43E-01	1.260
D08	Stachyose	2.099	1.761	2.40E-01	3.61E-01	0.839
B08	a-Methyl-D-Glucoside	1.857	2.228	2.73E-01	4.03E-01	1.200
E08	Sedoheptulosan	2.120	2.419	2.90E-01	4.22E-01	1.141
A02	NegativeControl	2.205	1.911	3.08E-01	4.41E-01	0.866
A07	Maltitol	1.584	1.345	3.26E-01	4.54E-01	0.849
F08	1,2-Propanediol	2.106	2.522	3.26E-01	4.54E-01	1.197
H06	g-Hydroxy-ButyricAcid	1.623	1.775	3.66E-01	5.02E-01	1.094
D06	D-Fructose-6-Phosphate	2.733	2.228	3.86E-01	5.15E-01	0.815
F01	Adonitol	1.585	1.402	3.86E-01	5.15E-01	0.885
G05	PyruvicAcid	1.881	1.671	4.08E-01	5.36E-01	0.888
B11	D-Sorbitol	1.646	2.006	4.53E-01	5.88E-01	1.219
G08	SuccinicAcid	2.398	2.590	4.76E-01	6.10E-01	1.080
B07	3-MethylGlucose	1.584	1.707	5.51E-01	6.87E-01	1.078
F02	L-Arabinose	2.309	2.181	5.51E-01	6.87E-01	0.945
B02	D-Glucose-1-Phosphate	3.959	3.403	6.58E-01	8.10E-01	0.859
A12	Gentiobiose	1.196	1.252	7.15E-01	8.58E-01	1.046
C04	Mannan	1.594	1.639	7.15E-01	8.58E-01	1.028
F09	2-Aminoethanol	1.293	1.340	7.44E-01	8.70E-01	1.036
H02	g-AminoButyricAcid	2.307	2.209	7.44E-01	8.70E-01	0.957
D11	Lactulose	1.872	1.904	7.73E-01	8.94E-01	1.017
A04	a-Cyclodextrin	1.590	1.522	8.02E-01	8.98E-01	0.957
C09	D-Melezitose	1.225	1.197	8.02E-01	8.98E-01	0.978
C10	Sucrose	1.575	1.609	8.32E-01	8.98E-01	1.022
F04	b-Methyl-D-Xyloside	1.583	1.537	8.32E-01	8.98E-01	0.971
G09	MonoMethylSuccinate	2.173	2.320	8.32E-01	8.98E-01	1.068
G11	D-MalicAcid	1.749	1.713	8.32E-01	8.98E-01	0.980
A09	Maltose	3.182	2.757	8.62E-01	9.10E-01	0.867
C11	Palatinose	2.205	2.153	8.62E-01	9.10E-01	0.976
D10	Lactitol	1.578	1.603	8.93E-01	9.22E-01	1.016
E10	Uridine	1.580	1.610	8.93E-01	9.22E-01	1.019
D07	D-Fructose	1.669	1.802	9.23E-01	9.43E-01	1.080
A11	D-Cellobiose	1.726	1.618	9.85E-01	9.85E-01	0.937
D01	D-Tagatose	1.597	1.569	9.85E-01	9.85E-01	0.983

PMM6_Post-C-19 Control to Pre-C-19 Control

Well	Well Compound	Mean Pre-C-19 Control	Mean Post-C-19 Control	Unadjusted p-value	Adjusted p-value	Fold difference in NADH production
A03	NegativeControl	6.18336	4.16496	1.91E-03	8.64E-02	0.674
A04	NegativeControl	7.12451	5.01883	3.90E-03	8.64E-02	0.704
C01	Epinephrine	5.82498	3.9935	6.01E-03	8.64E-02	0.686
C02	Epinephrine	6.35224	3.91789	2.33E-03	8.64E-02	0.617
C06	Epinephrine	5.36964	4.1003	8.10E-03	8.64E-02	0.764
D01	L-Leucine	5.70204	4.3306	8.10E-03	8.64E-02	0.759
E12	Thyroxine	2.24129	3.92308	3.66E-03	8.64E-02	1.750
F04	Dexamethasone	3.99213	2.64169	6.78E-03	8.64E-02	0.662
G01	Progesterone	5.3769	3.92736	6.01E-03	8.64E-02	0.730
A01	NegativeControl	5.64947	4.12094	1.14E-02	9.10E-02	0.729
A02	NegativeControl	5.90659	4.49113	1.98E-02	9.10E-02	0.760
A05	NegativeControl	5.63821	4.03512	1.02E-02	9.10E-02	0.716
B05	3-Isobutyl-1-Methylxanthine	0.98072	1.36596	1.68E-02	9.10E-02	1.393
C03	Epinephrine	6.47668	5.13269	2.08E-02	9.10E-02	0.792
E03	Triiodothyronine	6.80374	5.34267	1.88E-02	9.10E-02	0.785
E04	Triiodothyronine	7.66357	5.92764	1.43E-02	9.10E-02	0.773
E05	Triiodothyronine	5.45763	4.27844	1.78E-02	9.10E-02	0.784
F05	Dexamethasone	4.2693	2.88073	1.43E-02	9.10E-02	0.675
F06	Dexamethasone	4.77111	3.19332	1.35E-02	9.10E-02	0.669
F07	Hydrocortisone	4.85305	3.74977	1.98E-02	9.10E-02	0.773
G04	Progesterone	7.13574	5.38662	2.08E-02	9.10E-02	0.755
G06	Progesterone	6.97966	5.04013	1.35E-02	9.10E-02	0.722
F01	Dexamethasone	6.79684	5.23704	2.20E-02	9.17E-02	0.771
F02	Dexamethasone	6.86259	5.15575	2.44E-02	9.75E-02	0.751
F03	Dexamethasone	6.82206	4.84515	2.57E-02	9.85E-02	0.710
C05	Epinephrine	4.6234	3.69684	2.84E-02	1.05E-01	0.800
E01	Triiodothyronine	5.84046	4.7231	2.99E-02	1.06E-01	0.809
E06	Triiodothyronine	5.28642	4.13454	3.14E-02	1.08E-01	0.782
A06	NegativeControl	5.98373	4.38072	3.30E-02	1.09E-01	0.732
B11	Caffeine	2.59198	3.90101	3.47E-02	1.11E-01	1.505
E07	Thyroxine	5.19423	4.1886	4.41E-02	1.32E-01	0.806
H04	4,5-a-Dihydrotestosterone	6.18462	4.71724	4.41E-02	1.32E-01	0.763
A07	Dibutyryl-cAMP	5.48346	4.12388	5.82E-02	1.69E-01	0.752
C12	Norepinephrine	3.44207	4.36884	6.09E-02	1.72E-01	1.269
C04	Epinephrine	7.65599	6.32723	6.37E-02	1.75E-01	0.826
D02	L-Leucine	7.68449	6.33856	6.95E-02	1.80E-01	0.825
D04	L-Leucine	6.78693	5.5137	6.95E-02	1.80E-01	0.812
C11	Norepinephrine	4.90575	6.06708	9.37E-02	2.09E-01	1.237
D05	L-Leucine	3.97703	3.22761	8.62E-02	2.09E-01	0.812
G05	Progesterone	4.16749	3.40608	8.99E-02	2.09E-01	0.817
H02	4,5-a-Dihydrotestosterone	7.39416	6.18046	9.37E-02	2.09E-01	0.836
H03	4,5-a-Dihydrotestosterone	7.14843	5.904	8.99E-02	2.09E-01	0.826
H05	4,5-a-Dihydrotestosterone	7.00022	5.82267	8.62E-02	2.09E-01	0.832
D03	L-Leucine	7.74875	6.48551	9.77E-02	2.13E-01	0.837
H12	Aldosterone	4.00592	4.88651	1.02E-01	2.17E-01	1.220
G03	Progesterone	4.61152	3.74568	1.06E-01	2.21E-01	0.812
H01	4,5-a-Dihydrotestosterone	5.45695	4.67857	1.29E-01	2.64E-01	0.857
B12	Caffeine	1.43671	1.64481	1.34E-01	2.68E-01	1.145
D12	Creatine	4.02721	4.77841	1.39E-01	2.73E-01	1.187
D07	Creatine	4.76009	4.00137	1.56E-01	3.00E-01	0.841
H10	Aldosterone	4.84825	5.65548	1.62E-01	3.05E-01	1.166
F12	Hydrocortisone	3.00501	3.50461	1.74E-01	3.22E-01	1.166
E11	Thyroxine	4.14601	5.02931	1.87E-01	3.33E-01	1.213
G02	Progesterone	7.00725	5.97947	1.87E-01	3.33E-01	0.853
C08	Norepinephrine	4.48602	3.95657	2.01E-01	3.50E-01	0.882
G07	beta-Estradiol	6.20371	5.36514	2.15E-01	3.62E-01	0.865
H09	Aldosterone	4.09671	4.57469	2.15E-01	3.62E-01	1.117
C10	Norepinephrine	4.15435	4.84421	2.30E-01	3.68E-01	1.166
D11	Creatine	4.85744	5.69751	2.30E-01	3.68E-01	1.173

F08	Hydrocortisone	4.94827	4.26597	2.23E-01	3.68E-01	0.862
A08	Dibutyryl-cAMP	5.67144	4.83725	2.46E-01	3.87E-01	0.853
H11	Aldosterone	4.52774	5.1552	2.54E-01	3.93E-01	1.139
A12	Dibutyryl-cAMP	3.3225	4.45671	2.80E-01	4.26E-01	1.341
H06	4,5-a-Dihydrotestosterone	6.53666	5.8361	2.89E-01	4.33E-01	0.893
B10	Caffeine	2.82448	3.36178	2.98E-01	4.40E-01	1.190
C07	Norepinephrine	5.19657	4.67235	3.36E-01	4.89E-01	0.899
E02	Triiodothyronine	7.44826	6.7024	3.46E-01	4.96E-01	0.900
G12	beta-Estradiol	3.82968	4.11805	3.56E-01	4.96E-01	1.075
H07	Aldosterone	5.42794	4.93701	3.56E-01	4.96E-01	0.910
E10	Thyroxine	4.42593	4.98746	3.67E-01	5.03E-01	1.127
D06	L-Leucine	3.50884	3.04504	3.77E-01	5.10E-01	0.868
B01	3-Isobutyl-1-Methylxanthine	6.38522	6.01678	4.10E-01	5.47E-01	0.942
G10	beta-Estradiol	4.60307	4.93021	4.22E-01	5.55E-01	1.071
B07	Caffeine	6.88969	6.58417	4.33E-01	5.62E-01	0.956
F10	Hydrocortisone	3.45098	3.67168	4.45E-01	5.70E-01	1.064
F11	Hydrocortisone	3.8069	3.95965	4.57E-01	5.77E-01	1.040
D08	Creatine	5.62532	5.12121	4.69E-01	5.85E-01	0.910
A11	Dibutyryl-cAMP	4.83553	5.81396	5.06E-01	6.23E-01	1.202
G08	beta-Estradiol	4.54163	4.24152	5.32E-01	6.47E-01	0.934
B04	3-Isobutyl-1-Methylxanthine	3.04958	3.49066	5.58E-01	6.70E-01	1.145
B02	3-Isobutyl-1-Methylxanthine	7.55383	7.24594	5.72E-01	6.78E-01	0.959
G11	beta-Estradiol	4.76837	4.83724	5.85E-01	6.85E-01	1.014
B06	3-Isobutyl-1-Methylxanthine	1.86579	0.7959	6.13E-01	7.09E-01	0.427
B09	Caffeine	5.01416	5.37189	6.27E-01	7.17E-01	1.071
E08	Thyroxine	6.12783	5.75166	6.41E-01	7.24E-01	0.939
A10	Dibutyryl-cAMP	5.41817	6.28859	6.55E-01	7.32E-01	1.161
D09	Creatine	5.10628	5.3073	6.84E-01	7.55E-01	1.039
G09	beta-Estradiol	4.80118	4.84501	7.59E-01	8.27E-01	1.009
A09	Dibutyryl-cAMP	4.07441	4.27469	7.89E-01	8.51E-01	1.049
H08	Aldosterone	5.12691	4.92073	8.20E-01	8.74E-01	0.960
E09	Thyroxine	3.32296	3.43728	8.35E-01	8.81E-01	1.034
C09	Norepinephrine	4.33994	4.26824	8.82E-01	9.10E-01	0.983
F09	Hydrocortisone	4.88466	4.70471	8.82E-01	9.10E-01	0.963
D10	Creatine	4.15744	4.41234	8.97E-01	9.17E-01	1.061
B03	3-Isobutyl-1-Methylxanthine	5.74357	5.92518	9.29E-01	9.39E-01	1.032
B08	Caffeine	4.65401	4.54543	9.60E-01	9.60E-01	0.977

PMM6_Post-C-19 Control to LC-19

Well	Well Compound	Mean Post-C-19 Control	Mean LC-19	Unadjusted p-value	Adjusted p-value	Fold difference in NADH production
A01	NegativeControl	4.12094	2.28804	1.47E-02	1.28E-01	0.555
A02	NegativeControl	4.49113	2.59839	1.85E-02	1.28E-01	0.579
A03	NegativeControl	4.16496	2.58749	3.55E-02	1.28E-01	0.621
A04	NegativeControl	5.01883	3.04228	4.33E-02	1.28E-01	0.606
A05	NegativeControl	4.03512	2.3443	2.88E-02	1.28E-01	0.581
A06	NegativeControl	4.38072	2.60796	6.30E-02	1.28E-01	0.595
A09	Dibutyryl-cAMP	4.27469	2.62891	7.53E-02	1.28E-01	0.615
A12	Dibutyryl-cAMP	4.45671	2.49207	6.30E-02	1.28E-01	0.559
B01	3-Isobutyl-1-Methylxanthine	6.01678	3.71384	2.32E-02	1.28E-01	0.617
B02	3-Isobutyl-1-Methylxanthine	7.24594	4.59064	8.92E-02	1.28E-01	0.634
B03	3-Isobutyl-1-Methylxanthine	5.92518	3.61994	7.53E-02	1.28E-01	0.611
B07	Caffeine	6.58417	4.55379	8.92E-02	1.28E-01	0.692
B08	Caffeine	4.54543	3.18946	8.92E-02	1.28E-01	0.702
C01	Epinephrine	3.9935	2.92221	8.92E-02	1.28E-01	0.732
C03	Epinephrine	5.13269	3.44225	5.24E-02	1.28E-01	0.671
C04	Epinephrine	6.32723	4.201	6.30E-02	1.28E-01	0.664
C05	Epinephrine	3.69684	2.42943	4.33E-02	1.28E-01	0.657
C06	Epinephrine	4.1003	2.80111	4.33E-02	1.28E-01	0.683
C07	Norepinephrine	4.67235	3.13908	4.33E-02	1.28E-01	0.672
C09	Norepinephrine	4.26824	2.93256	7.53E-02	1.28E-01	0.687
C10	Norepinephrine	4.84421	3.38077	8.92E-02	1.28E-01	0.698
C12	Norepinephrine	4.36884	3.03392	2.32E-02	1.28E-01	0.694
D01	L-Leucine	4.3306	2.93167	3.55E-02	1.28E-01	0.677
D02	L-Leucine	6.33856	4.2661	6.95E-02	1.28E-01	0.673
D03	L-Leucine	6.48551	3.82472	8.93E-03	1.28E-01	0.590
D04	L-Leucine	5.5137	3.71709	6.30E-02	1.28E-01	0.674
D05	L-Leucine	3.22761	2.2039	5.24E-02	1.28E-01	0.683
D06	L-Leucine	3.04504	2.17303	6.30E-02	1.28E-01	0.714
D07	Creatine	4.00137	2.41408	2.88E-02	1.28E-01	0.603
D08	Creatine	5.12121	3.15002	1.85E-02	1.28E-01	0.615
D09	Creatine	5.3073	3.26505	2.32E-02	1.28E-01	0.615
D10	Creatine	4.41234	2.72336	3.55E-02	1.28E-01	0.617
D12	Creatine	4.77841	3.36381	6.30E-02	1.28E-01	0.704
E01	Triiodothyronine	4.7231	3.36325	6.30E-02	1.28E-01	0.712
E02	Triiodothyronine	6.7024	4.21384	2.88E-02	1.28E-01	0.629
E03	Triiodothyronine	5.34267	3.27778	2.32E-02	1.28E-01	0.614
E04	Triiodothyronine	5.92764	3.84127	3.12E-02	1.28E-01	0.648
E05	Triiodothyronine	4.27844	2.92099	8.92E-02	1.28E-01	0.683
E10	Thyroxine	4.98746	3.33969	3.55E-02	1.28E-01	0.670
E11	Thyroxine	5.02931	3.23868	2.32E-02	1.28E-01	0.644
E12	Thyroxine	3.92308	2.15133	6.84E-03	1.28E-01	0.548
F01	Dexamethasone	5.23704	3.38583	2.88E-02	1.28E-01	0.647
F02	Dexamethasone	5.15575	3.3018	4.33E-02	1.28E-01	0.640
F03	Dexamethasone	4.84515	3.14094	5.24E-02	1.28E-01	0.648
F04	Dexamethasone	2.64169	1.69733	3.55E-02	1.28E-01	0.643
F05	Dexamethasone	2.88073	1.90324	5.24E-02	1.28E-01	0.661
F06	Dexamethasone	3.19332	2.13385	2.88E-02	1.28E-01	0.668
F07	Hydrocortisone	3.74977	2.60318	7.53E-02	1.28E-01	0.694
F09	Hydrocortisone	4.70471	3.40247	8.92E-02	1.28E-01	0.723
F10	Hydrocortisone	3.67168	2.49318	4.33E-02	1.28E-01	0.679
F11	Hydrocortisone	3.95965	2.80216	6.30E-02	1.28E-01	0.708
F12	Hydrocortisone	3.50461	2.5435	6.30E-02	1.28E-01	0.726
G01	Progesterone	3.92736	2.98411	7.53E-02	1.28E-01	0.760
G02	Progesterone	5.97947	4.02493	4.33E-02	1.28E-01	0.673
G04	Progesterone	5.38662	3.7055	7.53E-02	1.28E-01	0.688
G05	Progesterone	3.40608	2.21153	6.30E-02	1.28E-01	0.649
G07	beta-Estradiol	5.36514	3.92745	8.92E-02	1.28E-01	0.732
G11	beta-Estradiol	4.83724	3.67303	8.92E-02	1.28E-01	0.759
H01	4,5-a-Dihydrotestosterone	4.67857	3.35444	7.53E-02	1.28E-01	0.717
H02	4,5-a-Dihydrotestosterone	6.18046	4.42703	7.53E-02	1.28E-01	0.716
H03	4,5-a-Dihydrotestosterone	5.904	4.01016	3.55E-02	1.28E-01	0.679

H06	4,5-a-Dihydrotestosterone	5.8361	4.13823	7.53E-02	1.28E-01	0.709
H08	Aldosterone	4.92073	3.4467	5.24E-02	1.28E-01	0.700
H09	Aldosterone	4.57469	3.34512	8.92E-02	1.28E-01	0.731
H10	Aldosterone	5.65548	4.03347	8.89E-02	1.28E-01	0.713
H11	Aldosterone	5.1552	3.70911	6.30E-02	1.28E-01	0.719
H12	Aldosterone	4.88651	3.41818	5.24E-02	1.28E-01	0.700
A07	Dibutyryl-cAMP	4.12388	2.49284	1.05E-01	1.33E-01	0.604
A08	Dibutyryl-cAMP	4.83725	3.02963	1.05E-01	1.33E-01	0.626
A10	Dibutyryl-cAMP	6.28859	3.93058	1.05E-01	1.33E-01	0.625
B04	3-Isobutyl-1-Methylxanthine	3.49066	2.3232	1.05E-01	1.33E-01	0.666
C11	Norepinephrine	6.06708	4.21323	1.05E-01	1.33E-01	0.694
D11	Creatine	5.69751	4.08347	1.05E-01	1.33E-01	0.717
E06	Triiodothyronine	4.13454	2.78509	1.05E-01	1.33E-01	0.674
F08	Hydrocortisone	4.26597	3.10113	1.05E-01	1.33E-01	0.727
G08	beta-Estradiol	4.24152	3.10284	1.05E-01	1.33E-01	0.732
A11	Dibutyryl-cAMP	5.81396	3.56506	1.23E-01	1.46E-01	0.613
B09	Caffeine	5.37189	3.93415	1.23E-01	1.46E-01	0.732
G03	Progesterone	3.74568	2.72202	1.23E-01	1.46E-01	0.727
G12	beta-Estradiol	4.11805	3.03299	1.23E-01	1.46E-01	0.737
H05	4,5-a-Dihydrotestosterone	5.82267	4.17966	1.23E-01	1.46E-01	0.718
H07	Aldosterone	4.93701	3.60218	1.30E-01	1.53E-01	0.730
C08	Norepinephrine	3.95657	2.88534	1.43E-01	1.62E-01	0.729
E08	Thyroxine	5.75166	4.15291	1.43E-01	1.62E-01	0.722
E09	Thyroxine	3.43728	2.5141	1.43E-01	1.62E-01	0.731
E07	Thyroxine	4.1886	3.13169	1.65E-01	1.85E-01	0.748
B10	Caffeine	3.36178	2.53803	1.90E-01	2.05E-01	0.755
B11	Caffeine	3.90101	2.79027	1.90E-01	2.05E-01	0.715
G10	beta-Estradiol	4.93021	3.69418	1.90E-01	2.05E-01	0.749
B06	3-Isobutyl-1-Methylxanthine	0.7959	0.90238	2.12E-01	2.26E-01	1.134
H04	4,5-a-Dihydrotestosterone	4.71724	3.5624	2.18E-01	2.30E-01	0.755
G06	Progesterone	5.04013	3.64911	2.47E-01	2.58E-01	0.724
B12	Caffeine	1.64481	1.35872	3.93E-01	4.01E-01	0.826
G09	beta-Estradiol	4.84501	3.83978	3.93E-01	4.01E-01	0.793
C02	Epinephrine	3.91789	3.3843	5.29E-01	5.34E-01	0.864
B05	3-Isobutyl-1-Methylxanthine	1.36596	1.28062	9.71E-01	9.71E-01	0.938

PMM6_Post-C-19 Control to LC-19 more than 4 symptoms

Well	Well compound	Mean Post-C-19 Control	Mean LC-19 >4 symptoms	Unadjusted p-value	Adjusted p-value	Fold difference in NADH production
A01	NegativeControl	4.121	2.051	1.10E-02	2.78E-02	0.498
A02	NegativeControl	4.491	2.150	1.10E-02	2.78E-02	0.479
B01	3-Isobutyl-1-Methylxanthine	6.017	2.763	7.49E-03	2.78E-02	0.459
C03	Epinephrine	5.133	2.460	4.75E-03	2.78E-02	0.479
C04	Epinephrine	6.327	3.067	1.10E-02	2.78E-02	0.485
C05	Epinephrine	3.697	1.758	4.75E-03	2.78E-02	0.476
C06	Epinephrine	4.100	1.990	3.00E-03	2.78E-02	0.485
C07	Norepinephrine	4.672	2.205	3.00E-03	2.78E-02	0.472
C09	Norepinephrine	4.268	2.023	7.49E-03	2.78E-02	0.474
C10	Norepinephrine	4.844	2.417	1.10E-02	2.78E-02	0.499
C12	Norepinephrine	4.369	2.294	1.10E-02	2.78E-02	0.525
D01	L-Leucine	4.331	2.138	7.49E-03	2.78E-02	0.494
D03	L-Leucine	6.486	2.929	4.75E-03	2.78E-02	0.452
D04	L-Leucine	5.514	2.768	1.10E-02	2.78E-02	0.502
D05	L-Leucine	3.228	1.555	7.49E-03	2.78E-02	0.482
D06	L-Leucine	3.045	1.503	4.75E-03	2.78E-02	0.494
D08	Creatine	5.121	2.645	1.10E-02	2.78E-02	0.516
E01	Triiodothyronine	4.723	2.476	1.10E-02	2.78E-02	0.524
E02	Triiodothyronine	6.702	2.865	3.00E-03	2.78E-02	0.428
E03	Triiodothyronine	5.343	2.200	3.00E-03	2.78E-02	0.412
E04	Triiodothyronine	5.928	2.762	3.00E-03	2.78E-02	0.466
E11	Thyroxine	5.029	2.599	1.10E-02	2.78E-02	0.517
E12	Thyroxine	3.923	1.789	1.10E-02	2.78E-02	0.456
F02	Dexamethasone	5.156	2.418	7.49E-03	2.78E-02	0.469
F10	Hydrocortisone	3.672	1.926	1.10E-02	2.78E-02	0.524
G01	Progesterone	3.927	2.061	3.00E-03	2.78E-02	0.525
G02	Progesterone	5.979	2.698	3.00E-03	2.78E-02	0.451
G04	Progesterone	5.387	2.595	1.10E-02	2.78E-02	0.482
G05	Progesterone	3.406	1.543	7.49E-03	2.78E-02	0.453
G08	beta-Estradiol	4.242	2.195	1.10E-02	2.78E-02	0.517
G11	beta-Estradiol	4.837	2.684	1.10E-02	2.78E-02	0.555
H01	4,5-a-Dihydrotestosterone	4.679	2.386	7.49E-03	2.78E-02	0.510
H02	4,5-a-Dihydrotestosterone	6.180	3.050	7.49E-03	2.78E-02	0.494
H03	4,5-a-Dihydrotestosterone	5.904	2.916	4.75E-03	2.78E-02	0.494
H08	Aldosterone	4.921	2.496	7.49E-03	2.78E-02	0.507
H09	Aldosterone	4.575	2.402	1.10E-02	2.78E-02	0.525
H11	Aldosterone	5.155	2.859	1.10E-02	2.78E-02	0.555
H12	Aldosterone	4.887	2.516	7.49E-03	2.78E-02	0.515
B08	Caffeine	4.545	2.264	1.60E-02	3.01E-02	0.498
B11	Caffeine	3.901	1.893	1.60E-02	3.01E-02	0.485
D09	Creatine	5.307	2.692	1.60E-02	3.01E-02	0.507
D11	Creatine	5.698	2.971	1.60E-02	3.01E-02	0.521
D12	Creatine	4.778	2.584	1.60E-02	3.01E-02	0.541
E05	Triiodothyronine	4.278	2.167	1.60E-02	3.01E-02	0.506
E10	Thyroxine	4.987	2.483	1.60E-02	3.01E-02	0.498
F01	Dexamethasone	5.237	2.604	1.60E-02	3.01E-02	0.497
F07	Hydrocortisone	3.750	1.938	1.60E-02	3.01E-02	0.517
F11	Hydrocortisone	3.960	2.219	1.60E-02	3.01E-02	0.560
G03	Progesterone	3.746	1.856	1.60E-02	3.01E-02	0.495
G07	beta-Estradiol	5.365	2.865	1.60E-02	3.01E-02	0.534
H06	4,5-a-Dihydrotestosterone	5.836	3.082	1.60E-02	3.01E-02	0.528
B04	3-Isobutyl-1-Methylxanthine	3.491	1.826	2.25E-02	3.24E-02	0.523
B07	Caffeine	6.584	3.340	2.25E-02	3.24E-02	0.507
C01	Epinephrine	3.994	2.060	2.25E-02	3.24E-02	0.516
C11	Norepinephrine	6.067	3.175	2.25E-02	3.24E-02	0.523
D02	L-Leucine	6.339	3.023	2.25E-02	3.24E-02	0.477

D07	Creatine	4.001	2.014	2.25E-02	3.24E-02	0.503
E06	Triiodothyronine	4.135	2.084	2.25E-02	3.24E-02	0.504
F03	Dexamethasone	4.845	2.414	2.25E-02	3.24E-02	0.498
F04	Dexamethasone	2.642	1.413	2.25E-02	3.24E-02	0.535
F06	Dexamethasone	3.193	1.778	2.25E-02	3.24E-02	0.557
F08	Hydrocortisone	4.266	2.337	2.25E-02	3.24E-02	0.548
F09	Hydrocortisone	4.705	2.602	2.25E-02	3.24E-02	0.553
G10	beta-Estradiol	4.930	2.683	2.25E-02	3.24E-02	0.544
G12	beta-Estradiol	4.118	2.285	2.25E-02	3.24E-02	0.555
H05	4,5-a-Dihydrotestosterone	5.823	3.070	2.25E-02	3.24E-02	0.527
H07	Aldosterone	4.937	2.664	2.26E-02	3.24E-02	0.540
B02	3-Isobutyl-1-Methylxanthine	7.246	3.531	3.12E-02	3.84E-02	0.487
B03	3-Isobutyl-1-Methylxanthine	5.925	2.899	3.12E-02	3.84E-02	0.489
B09	Caffeine	5.372	2.893	3.12E-02	3.84E-02	0.538
B10	Caffeine	3.362	1.831	3.12E-02	3.84E-02	0.545
C08	Norepinephrine	3.957	2.075	3.12E-02	3.84E-02	0.525
D10	Creatine	4.412	2.314	3.12E-02	3.84E-02	0.525
E07	Thyroxine	4.189	2.277	3.12E-02	3.84E-02	0.544
E08	Thyroxine	5.752	3.022	3.12E-02	3.84E-02	0.525
E09	Thyroxine	3.437	1.823	3.12E-02	3.84E-02	0.531
F05	Dexamethasone	2.881	1.577	3.12E-02	3.84E-02	0.547
F12	Hydrocortisone	3.505	2.106	3.12E-02	3.84E-02	0.601
H10	Aldosterone	5.655	3.061	3.43E-02	4.17E-02	0.541
A03	NegativeControl	4.165	2.196	4.20E-02	4.97E-02	0.527
A05	NegativeControl	4.035	2.159	4.20E-02	4.97E-02	0.535
G06	Progesterone	5.040	2.765	5.59E-02	6.47E-02	0.549
H04	4,5-a-Dihydrotestosterone	4.717	2.562	5.59E-02	6.47E-02	0.543
A04	NegativeControl	5.019	2.754	7.27E-02	8.21E-02	0.549
A09	Dibutyryl-cAMP	4.275	2.391	7.27E-02	8.21E-02	0.559
A06	NegativeControl	4.381	2.337	9.34E-02	9.96E-02	0.533
A08	Dibutyryl-cAMP	4.837	2.714	9.34E-02	9.96E-02	0.561
A11	Dibutyryl-cAMP	5.814	3.284	9.34E-02	9.96E-02	0.565
A12	Dibutyryl-cAMP	4.457	2.434	9.34E-02	9.96E-02	0.546
G09	beta-Estradiol	4.845	2.818	9.34E-02	9.96E-02	0.582
A07	Dibutyryl-cAMP	4.124	2.353	1.18E-01	1.23E-01	0.571
A10	Dibutyryl-cAMP	6.289	3.621	1.18E-01	1.23E-01	0.576
B12	Caffeine	1.645	1.233	1.81E-01	1.86E-01	0.750
C02	Epinephrine	3.918	2.483	2.63E-01	2.69E-01	0.634
B06	3-Isobutyl-1-Methylxanthine	0.796	0.896	3.56E-01	3.60E-01	1.126
B05	3-Isobutyl-1-Methylxanthine	1.366	1.149	6.35E-01	6.35E-01	0.841

PMM6_Post-C-19 Control to LC-19 Official Diagnosis

Well	Well compound	Control	official	value	value	in NADH
A01	NegativeControl	4.121	1.218	6.99E-03	1.43E-02	0.295
A02	NegativeControl	4.491	1.234	6.99E-03	1.43E-02	0.275
A11	Dibutyryl-cAMP	5.814	1.269	6.99E-03	1.43E-02	0.218
B01	3-Isobutyl-1-Methylxanthine	6.017	1.663	6.99E-03	1.43E-02	0.276
B02	3-Isobutyl-1-Methylxanthine	7.246	1.892	6.99E-03	1.43E-02	0.261
B03	3-Isobutyl-1-Methylxanthine	5.925	1.528	6.99E-03	1.43E-02	0.258
B04	3-Isobutyl-1-Methylxanthine	3.491	1.129	6.99E-03	1.43E-02	0.323
B07	Caffeine	6.584	1.774	6.99E-03	1.43E-02	0.269
B08	Caffeine	4.545	1.309	6.99E-03	1.43E-02	0.288
B09	Caffeine	5.372	1.656	6.99E-03	1.43E-02	0.308
B10	Caffeine	3.362	1.069	6.99E-03	1.43E-02	0.318
C03	Epinephrine	5.133	1.467	6.99E-03	1.43E-02	0.286
C04	Epinephrine	6.327	1.720	6.99E-03	1.43E-02	0.272
C05	Epinephrine	3.697	1.039	6.99E-03	1.43E-02	0.281
C06	Epinephrine	4.100	1.174	6.99E-03	1.43E-02	0.286
C07	Norepinephrine	4.672	1.279	6.99E-03	1.43E-02	0.274
C08	Norepinephrine	3.957	1.154	6.99E-03	1.43E-02	0.292
C09	Norepinephrine	4.268	1.176	6.99E-03	1.43E-02	0.276
C10	Norepinephrine	4.844	1.398	6.99E-03	1.43E-02	0.288
C11	Norepinephrine	6.067	1.826	6.99E-03	1.43E-02	0.301
C12	Norepinephrine	4.369	1.442	6.99E-03	1.43E-02	0.330
D01	L-Leucine	4.331	1.384	6.99E-03	1.43E-02	0.320
D02	L-Leucine	6.339	1.743	6.99E-03	1.43E-02	0.275
D03	L-Leucine	6.486	1.794	6.99E-03	1.43E-02	0.277
D04	L-Leucine	5.514	1.692	6.99E-03	1.43E-02	0.307
D05	L-Leucine	3.228	0.985	6.99E-03	1.43E-02	0.305
D06	L-Leucine	3.045	1.140	6.99E-03	1.43E-02	0.374
D07	Creatine	4.001	1.139	6.99E-03	1.43E-02	0.285
D08	Creatine	5.121	1.606	6.99E-03	1.43E-02	0.314
D09	Creatine	5.307	1.571	6.99E-03	1.43E-02	0.296
D10	Creatine	4.412	1.342	6.99E-03	1.43E-02	0.304
D11	Creatine	5.698	1.740	6.99E-03	1.43E-02	0.305
D12	Creatine	4.778	1.577	6.99E-03	1.43E-02	0.330
E01	Triiodothyronine	4.723	1.469	6.99E-03	1.43E-02	0.311
E02	Triiodothyronine	6.702	1.742	6.99E-03	1.43E-02	0.260
E03	Triiodothyronine	5.343	1.467	6.99E-03	1.43E-02	0.275
E04	Triiodothyronine	5.928	1.686	6.99E-03	1.43E-02	0.284
E05	Triiodothyronine	4.278	1.226	6.99E-03	1.43E-02	0.286
E06	Triiodothyronine	4.135	1.180	6.99E-03	1.43E-02	0.285
E07	Thyroxine	4.189	1.284	6.99E-03	1.43E-02	0.306
E08	Thyroxine	5.752	1.670	6.99E-03	1.43E-02	0.290
E09	Thyroxine	3.437	1.039	6.99E-03	1.43E-02	0.302
E10	Thyroxine	4.987	1.480	6.99E-03	1.43E-02	0.297
E11	Thyroxine	5.029	1.470	6.99E-03	1.43E-02	0.292
F02	Dexamethasone	5.156	1.588	6.99E-03	1.43E-02	0.308
F07	Hydrocortisone	3.750	1.087	6.99E-03	1.43E-02	0.290
H11	Aldosterone	5.155	1.819	6.99E-03	1.43E-02	0.353
A03	NegativeControl	4.165	1.184	1.40E-02	2.10E-02	0.284
A04	NegativeControl	5.019	1.429	1.40E-02	2.10E-02	0.285
A05	NegativeControl	4.035	1.103	1.40E-02	2.10E-02	0.273
A08	Dibutyryl-cAMP	4.837	1.344	1.40E-02	2.10E-02	0.278
A09	Dibutyryl-cAMP	4.275	1.075	1.40E-02	2.10E-02	0.252
A12	Dibutyryl-cAMP	4.457	0.978	1.40E-02	2.10E-02	0.219
B11	Caffeine	3.901	1.358	1.40E-02	2.10E-02	0.348
F08	Hydrocortisone	4.266	1.382	1.40E-02	2.10E-02	0.324
F09	Hydrocortisone	4.705	1.540	1.40E-02	2.10E-02	0.327
F10	Hydrocortisone	3.672	1.224	1.40E-02	2.10E-02	0.333
F11	Hydrocortisone	3.960	1.398	1.40E-02	2.10E-02	0.353
G01	Progesterone	3.927	1.401	1.40E-02	2.10E-02	0.357
G02	Progesterone	5.979	1.702	1.40E-02	2.10E-02	0.285
G04	Progesterone	5.387	1.599	1.40E-02	2.10E-02	0.297
G07	beta-Estradiol	5.365	1.671	1.40E-02	2.10E-02	0.311

G08	beta-Estradiol	4.242	1.339	1.40E-02	2.10E-02	0.316
G10	beta-Estradiol	4.930	1.560	1.40E-02	2.10E-02	0.316
A07	Dibutyryl-cAMP	4.124	1.153	2.80E-02	3.24E-02	0.280
A10	Dibutyryl-cAMP	6.289	1.627	2.80E-02	3.24E-02	0.259
E12	Thyroxine	3.923	1.356	2.80E-02	3.24E-02	0.346
F03	Dexamethasone	4.845	1.588	2.80E-02	3.24E-02	0.328
F04	Dexamethasone	2.642	0.936	2.80E-02	3.24E-02	0.354
F05	Dexamethasone	2.881	1.079	2.80E-02	3.24E-02	0.374
F06	Dexamethasone	3.193	1.205	2.80E-02	3.24E-02	0.377
F12	Hydrocortisone	3.505	1.342	2.80E-02	3.24E-02	0.383
G03	Progesterone	3.746	1.154	2.80E-02	3.24E-02	0.308
G05	Progesterone	3.406	0.958	2.80E-02	3.24E-02	0.281
G06	Progesterone	5.040	1.637	2.80E-02	3.24E-02	0.325
H01	4,5-a-Dihydrotestosterone	4.679	1.676	2.80E-02	3.24E-02	0.358
H02	4,5-a-Dihydrotestosterone	6.180	2.053	2.80E-02	3.24E-02	0.332
H03	4,5-a-Dihydrotestosterone	5.904	1.972	2.80E-02	3.24E-02	0.334
H04	4,5-a-Dihydrotestosterone	4.717	1.633	2.80E-02	3.24E-02	0.346
H06	4,5-a-Dihydrotestosterone	5.836	1.999	2.80E-02	3.24E-02	0.343
H08	Aldosterone	4.921	1.616	2.80E-02	3.24E-02	0.328
H09	Aldosterone	4.575	1.504	2.80E-02	3.24E-02	0.329
H12	Aldosterone	4.887	1.767	2.80E-02	3.24E-02	0.362
H07	Aldosterone	4.937	1.638	4.22E-02	4.83E-02	0.332
A06	NegativeControl	4.381	1.165	4.90E-02	5.16E-02	0.266
C01	Epinephrine	3.994	1.435	4.90E-02	5.16E-02	0.359
F01	Dexamethasone	5.237	1.606	4.90E-02	5.16E-02	0.307
G09	beta-Estradiol	4.845	1.548	4.90E-02	5.16E-02	0.319
G11	beta-Estradiol	4.837	1.623	4.90E-02	5.16E-02	0.335
G12	beta-Estradiol	4.118	1.359	4.90E-02	5.16E-02	0.330
H05	4,5-a-Dihydrotestosterone	5.823	1.953	4.90E-02	5.16E-02	0.335
H10	Aldosterone	5.655	1.890	5.16E-02	5.38E-02	0.334
C02	Epinephrine	3.918	1.413	1.61E-01	1.66E-01	0.361
B12	Caffeine	1.645	1.223	2.87E-01	2.93E-01	0.744
B06	3-Isobutyl-1-Methylxanthine	0.796	0.890	5.54E-01	5.59E-01	1.119
B05	3-Isobutyl-1-Methylxanthine	1.366	1.088	6.92E-01	6.92E-01	0.797

PMM6_Pre-C-19 Control to LC-19

Well	Well Compound	Mean Pre-C-19 Control	Mean LC-19	Unadjusted p-value	Adjusted p-value	Fold difference in NADH production
A01	NegativeControl	5.64947	2.28804	1.00E-05	4.00E-04	0.405
A02	NegativeControl	5.90659	2.59839	2.00E-05	4.00E-04	0.440
A03	NegativeControl	6.18336	2.58749	1.00E-05	4.00E-04	0.418
A04	NegativeControl	7.12451	3.04228	2.00E-05	4.00E-04	0.427
A05	NegativeControl	5.63821	2.3443	2.00E-05	4.00E-04	0.416
F04	Dexamethasone	3.99213	1.69733	2.00E-05	4.00E-04	0.425
A06	NegativeControl	5.98373	2.60796	4.00E-05	4.60E-04	0.436
A07	Dibutyryl-cAMP	5.48346	2.49284	4.00E-05	4.60E-04	0.455
C01	Epinephrine	5.82498	2.92221	6.00E-05	5.10E-04	0.502
C06	Epinephrine	5.36964	2.80111	9.00E-05	5.10E-04	0.522
D01	L-Leucine	5.70204	2.93167	8.00E-05	5.10E-04	0.514
E03	Triiodothyronine	6.80374	3.27778	5.00E-05	5.10E-04	0.482
E04	Triiodothyronine	7.66357	3.84127	6.00E-05	5.10E-04	0.501
F01	Dexamethasone	6.79684	3.38583	8.00E-05	5.10E-04	0.498
F05	Dexamethasone	4.2693	1.90324	9.00E-05	5.10E-04	0.446
F06	Dexamethasone	4.77111	2.13385	9.00E-05	5.10E-04	0.447
G06	Progesterone	6.97966	3.64911	6.00E-05	5.10E-04	0.523
C02	Epinephrine	6.35224	3.3843	1.30E-04	5.60E-04	0.533
C05	Epinephrine	4.6234	2.42943	1.20E-04	5.60E-04	0.525
D03	L-Leucine	7.74875	3.82472	1.30E-04	5.60E-04	0.494
E06	Triiodothyronine	5.28642	2.78509	1.10E-04	5.60E-04	0.527
F02	Dexamethasone	6.86259	3.3018	1.30E-04	5.60E-04	0.481
F03	Dexamethasone	6.82206	3.14094	1.10E-04	5.60E-04	0.460
G04	Progesterone	7.13574	3.7055	1.60E-04	6.30E-04	0.519
D07	Creatine	4.76009	2.41408	2.00E-04	6.90E-04	0.507
E05	Triiodothyronine	5.45763	2.92099	2.00E-04	6.90E-04	0.535
F07	Hydrocortisone	4.85305	2.60318	2.00E-04	6.90E-04	0.536
H03	4,5-a-Dihydrotestosterone	7.14843	4.01016	2.00E-04	6.90E-04	0.561
C03	Epinephrine	6.47668	3.44225	2.50E-04	8.10E-04	0.531
C04	Epinephrine	7.65599	4.201	2.50E-04	8.10E-04	0.549
A08	Dibutyryl-cAMP	5.67144	3.02963	2.90E-04	8.80E-04	0.534
G05	Progesterone	4.16749	2.21153	2.90E-04	8.80E-04	0.531
G01	Progesterone	5.3769	2.98411	3.40E-04	1.00E-03	0.555
H04	4,5-a-Dihydrotestosterone	6.18462	3.5624	3.70E-04	1.05E-03	0.576
D04	L-Leucine	6.78693	3.71709	4.00E-04	1.06E-03	0.548
E01	Triiodothyronine	5.84046	3.36325	4.00E-04	1.06E-03	0.576
D05	L-Leucine	3.97703	2.2039	5.00E-04	1.30E-03	0.554
H05	4,5-a-Dihydrotestosterone	7.00022	4.17966	5.40E-04	1.36E-03	0.597
D02	L-Leucine	7.68449	4.2661	8.30E-04	2.05E-03	0.555
E02	Triiodothyronine	7.44826	4.21384	9.60E-04	2.30E-03	0.566
G02	Progesterone	7.00725	4.02493	1.03E-03	2.41E-03	0.574
D08	Creatine	5.62532	3.15002	1.18E-03	2.70E-03	0.560
H01	4,5-a-Dihydrotestosterone	5.45695	3.35444	1.36E-03	2.96E-03	0.615
H02	4,5-a-Dihydrotestosterone	7.39416	4.42703	1.36E-03	2.96E-03	0.599
G03	Progesterone	4.61152	2.72202	1.67E-03	3.56E-03	0.590
E07	Thyroxine	5.19423	3.13169	2.04E-03	4.26E-03	0.603
C07	Norepinephrine	5.19657	3.13908	2.18E-03	4.36E-03	0.604
H06	4,5-a-Dihydrotestosterone	6.53666	4.13823	2.18E-03	4.36E-03	0.633
B01	3-Isobutyl-1-Methylxanthine	6.38522	3.71384	2.49E-03	4.87E-03	0.582
F08	Hydrocortisone	4.94827	3.10113	4.15E-03	7.98E-03	0.627
G07	beta-Estradiol	6.20371	3.92745	4.71E-03	8.86E-03	0.633
D06	L-Leucine	3.50884	2.17303	5.32E-03	9.83E-03	0.619
H08	Aldosterone	5.12691	3.4467	7.19E-03	1.30E-02	0.672
H07	Aldosterone	5.42794	3.60218	7.63E-03	1.36E-02	0.664
B02	3-Isobutyl-1-Methylxanthine	7.55383	4.59064	8.10E-03	1.41E-02	0.608
A09	Dibutyryl-cAMP	4.07441	2.62891	9.10E-03	1.56E-02	0.645
C08	Norepinephrine	4.48602	2.88534	9.64E-03	1.62E-02	0.643

B07	Caffeine	6.88969	4.55379	1.02E-02	1.69E-02	0.661
E08	Thyroxine	6.12783	4.15291	1.14E-02	1.86E-02	0.678
D09	Creatine	5.10628	3.26505	1.35E-02	2.17E-02	0.639
G08	beta-Estradiol	4.54163	3.10284	1.51E-02	2.38E-02	0.683
B08	Caffeine	4.65401	3.18946	1.78E-02	2.75E-02	0.685
C09	Norepinephrine	4.33994	2.93256	1.88E-02	2.81E-02	0.676
D10	Creatine	4.15744	2.72336	1.88E-02	2.81E-02	0.655
B05	3-Isobutyl-1-Methylxanthine	0.98072	1.28062	2.31E-02	3.42E-02	1.306
B03	3-Isobutyl-1-Methylxanthine	5.74357	3.61994	2.44E-02	3.54E-02	0.630
F09	Hydrocortisone	4.88466	3.40247	2.70E-02	3.87E-02	0.697
A10	Dibutyryl-cAMP	5.41817	3.93058	4.84E-02	6.84E-02	0.725
F10	Hydrocortisone	3.45098	2.49318	5.31E-02	7.39E-02	0.722
A11	Dibutyryl-cAMP	4.83553	3.56506	5.56E-02	7.63E-02	0.737
E10	Thyroxine	4.42593	3.33969	6.37E-02	8.61E-02	0.755
G11	beta-Estradiol	4.76837	3.67303	6.65E-02	8.87E-02	0.770
E09	Thyroxine	3.32296	2.5141	7.92E-02	1.03E-01	0.757
F11	Hydrocortisone	3.8069	2.80216	7.92E-02	1.03E-01	0.736
G12	beta-Estradiol	3.82968	3.03299	1.06E-01	1.36E-01	0.792
G09	beta-Estradiol	4.80118	3.83978	1.19E-01	1.51E-01	0.800
A12	Dibutyryl-cAMP	3.3225	2.49207	1.24E-01	1.53E-01	0.750
B09	Caffeine	5.01416	3.93415	1.24E-01	1.53E-01	0.785
G10	beta-Estradiol	4.60307	3.69418	1.50E-01	1.83E-01	0.803
H10	Aldosterone	4.84825	4.03347	1.74E-01	2.09E-01	0.832
B04	3-Isobutyl-1-Methylxanthine	3.04958	2.3232	1.94E-01	2.27E-01	0.762
H09	Aldosterone	4.09671	3.34512	1.94E-01	2.27E-01	0.817
C10	Norepinephrine	4.15435	3.38077	2.01E-01	2.29E-01	0.814
D12	Creatine	4.02721	3.36381	2.01E-01	2.29E-01	0.835
E11	Thyroxine	4.14601	3.23868	2.08E-01	2.35E-01	0.781
B12	Caffeine	1.43671	1.35872	2.23E-01	2.46E-01	0.946
H11	Aldosterone	4.52774	3.70911	2.23E-01	2.46E-01	0.819
D11	Creatine	4.85744	4.08347	2.54E-01	2.77E-01	0.841
H12	Aldosterone	4.00592	3.41818	2.98E-01	3.21E-01	0.853
E12	Thyroxine	2.24129	2.15133	3.26E-01	3.48E-01	0.960
C11	Norepinephrine	4.90575	4.21323	3.36E-01	3.55E-01	0.859
C12	Norepinephrine	3.44207	3.03392	3.99E-01	4.17E-01	0.881
B06	3-Isobutyl-1-Methylxanthine	1.86579	0.90238	4.57E-01	4.72E-01	0.484
B10	Caffeine	2.82448	2.53803	5.06E-01	5.12E-01	0.899
F12	Hydrocortisone	3.00501	2.5435	5.06E-01	5.12E-01	0.846
B11	Caffeine	2.59198	2.79027	5.85E-01	5.85E-01	1.077

PMM6_Pre-C-19 Control to LC-19 more than 4 symptoms

Well	Well compound	Mean Pre-C-19 Control	Mean LC-19 >4 Symptoms	Unadjusted p-value	Adjusted p-value	Fold difference in NADH production
A01	NegativeControl	5.64947	2.05128	4.50E-04	1.30E-03	0.363
A02	NegativeControl	5.90659	2.1497	4.10E-04	1.30E-03	0.364
A03	NegativeControl	6.18336	2.19636	3.70E-04	1.30E-03	0.355
C01	Epinephrine	5.82498	2.05953	1.60E-04	1.30E-03	0.354
C02	Epinephrine	6.35224	2.48267	3.70E-04	1.30E-03	0.391
C03	Epinephrine	6.47668	2.45976	3.70E-04	1.30E-03	0.380
C04	Epinephrine	7.65599	3.0671	4.50E-04	1.30E-03	0.401
C05	Epinephrine	4.6234	1.75805	2.70E-04	1.30E-03	0.380
C06	Epinephrine	5.36964	1.9901	1.80E-04	1.30E-03	0.371
C07	Norepinephrine	5.19657	2.2046	4.10E-04	1.30E-03	0.424
D01	L-Leucine	5.70204	2.13825	2.40E-04	1.30E-03	0.375
D03	L-Leucine	7.74875	2.92905	3.70E-04	1.30E-03	0.378
D05	L-Leucine	3.97703	1.55512	3.70E-04	1.30E-03	0.391
E01	Triiodothyronine	5.84046	2.4757	3.30E-04	1.30E-03	0.424
E02	Triiodothyronine	7.44826	2.86534	3.30E-04	1.30E-03	0.385
E03	Triiodothyronine	6.80374	2.19999	1.20E-04	1.30E-03	0.323
E04	Triiodothyronine	7.66357	2.76233	2.40E-04	1.30E-03	0.360
E05	Triiodothyronine	5.45763	2.16701	4.50E-04	1.30E-03	0.397
E06	Triiodothyronine	5.28642	2.08372	3.30E-04	1.30E-03	0.394
F01	Dexamethasone	6.79684	2.60377	3.70E-04	1.30E-03	0.383
F02	Dexamethasone	6.86259	2.41788	3.30E-04	1.30E-03	0.352
F04	Dexamethasone	3.99213	1.41264	3.30E-04	1.30E-03	0.354
F07	Hydrocortisone	4.85305	1.93753	3.30E-04	1.30E-03	0.399
G01	Progesterone	5.3769	2.06076	2e-04	1.30E-03	0.383
G02	Progesterone	7.00725	2.6977	2.20E-04	1.30E-03	0.385
G03	Progesterone	4.61152	1.85566	3.70E-04	1.30E-03	0.402
G04	Progesterone	7.13574	2.59466	2.20E-04	1.30E-03	0.364
G05	Progesterone	4.16749	1.54338	2.40E-04	1.30E-03	0.370
G06	Progesterone	6.97966	2.76497	2.40E-04	1.30E-03	0.396
H02	4,5-a-Dihydrotestosterone	7.39416	3.05036	3e-04	1.30E-03	0.413
H03	4,5-a-Dihydrotestosterone	7.14843	2.91615	3.30E-04	1.30E-03	0.408
H04	4,5-a-Dihydrotestosterone	6.18462	2.56189	3.30E-04	1.30E-03	0.414
H05	4,5-a-Dihydrotestosterone	7.00022	3.06997	4.50E-04	1.30E-03	0.439
D04	L-Leucine	6.78693	2.76771	4.90E-04	1.36E-03	0.408
D06	L-Leucine	3.50884	1.50274	4.90E-04	1.36E-03	0.428
D02	L-Leucine	7.68449	3.02251	5.50E-04	1.42E-03	0.393
F03	Dexamethasone	6.82206	2.4143	5.50E-04	1.42E-03	0.354
D07	Creatine	4.76009	2.01358	6e-04	1.48E-03	0.423
H01	4,5-a-Dihydrotestosterone	5.45695	2.38641	6e-04	1.48E-03	0.437
H06	4,5-a-Dihydrotestosterone	6.53666	3.08246	6.60E-04	1.55E-03	0.472
H08	Aldosterone	5.12691	2.49597	6.60E-04	1.55E-03	0.487
F05	Dexamethasone	4.2693	1.57671	7.30E-04	1.67E-03	0.369
A04	NegativeControl	7.12451	2.75436	8.10E-04	1.76E-03	0.387
F06	Dexamethasone	4.77111	1.77766	8.10E-04	1.76E-03	0.373
A05	NegativeControl	5.63821	2.15918	8.90E-04	1.85E-03	0.383
E07	Thyroxine	5.19423	2.27663	8.90E-04	1.85E-03	0.438
G07	beta-Estradiol	6.20371	2.86468	9.70E-04	1.99E-03	0.462
G08	beta-Estradiol	4.54163	2.19466	1.07E-03	2.14E-03	0.483
F08	Hydrocortisone	4.94827	2.33672	1.17E-03	2.30E-03	0.472
A06	NegativeControl	5.98373	2.3366	1.29E-03	2.38E-03	0.390
C08	Norepinephrine	4.48602	2.07534	1.29E-03	2.38E-03	0.463
H07	Aldosterone	5.42794	2.66438	1.29E-03	2.38E-03	0.491
A07	Dibutyryl-cAMP	5.48346	2.35311	1.85E-03	3.30E-03	0.429
D08	Creatine	5.62532	2.64451	1.85E-03	3.30E-03	0.470
C09	Norepinephrine	4.33994	2.02297	2.03E-03	3.54E-03	0.466
B08	Caffeine	4.65401	2.26376	2.22E-03	3.80E-03	0.486
B07	Caffeine	6.88969	3.34002	2.42E-03	4.01E-03	0.485
E08	Thyroxine	6.12783	3.02161	2.42E-03	4.01E-03	0.493
B01	3-Isobutyl-1-Methylxanthine	6.38522	2.76313	3.42E-03	5.56E-03	0.433
G11	beta-Estradiol	4.76837	2.68411	4.40E-03	7.04E-03	0.563

A08	Dibutyryl-cAMP	5.67144	2.71383	4.78E-03	7.53E-03	0.479
F09	Hydrocortisone	4.88466	2.6017	6.11E-03	9.46E-03	0.533
E09	Thyroxine	3.32296	1.82349	7.17E-03	1.09E-02	0.549
B02	3-Isobutyl-1-Methylxanthine	7.55383	3.53081	8.39E-03	1.26E-02	0.467
F10	Hydrocortisone	3.45098	1.92563	9.07E-03	1.30E-02	0.558
G09	beta-Estradiol	4.80118	2.81838	9.07E-03	1.30E-02	0.587
H09	Aldosterone	4.09671	2.4018	9.07E-03	1.30E-02	0.586
D09	Creatine	5.10628	2.69245	9.80E-03	1.38E-02	0.527
G12	beta-Estradiol	3.82968	2.28472	1.06E-02	1.47E-02	0.597
G10	beta-Estradiol	4.60307	2.68254	1.14E-02	1.57E-02	0.583
D10	Creatine	4.15744	2.31442	1.23E-02	1.64E-02	0.557
E10	Thyroxine	4.42593	2.48261	1.23E-02	1.64E-02	0.561
B09	Caffeine	5.01416	2.89253	1.54E-02	2.02E-02	0.577
F11	Hydrocortisone	3.8069	2.21859	1.91E-02	2.47E-02	0.583
B03	3-Isobutyl-1-Methylxanthine	5.74357	2.89914	2.35E-02	2.93E-02	0.505
C10	Norepinephrine	4.15435	2.41698	2.35E-02	2.93E-02	0.582
D11	Creatine	4.85744	2.97073	2.35E-02	2.93E-02	0.612
H10	Aldosterone	4.84825	3.06117	2.52E-02	3.10E-02	0.631
D12	Creatine	4.02721	2.58442	3.30E-02	4.01E-02	0.642
A09	Dibutyryl-cAMP	4.07441	2.39078	3.52E-02	4.22E-02	0.587
H11	Aldosterone	4.52774	2.85933	3.76E-02	4.45E-02	0.632
H12	Aldosterone	4.00592	2.51617	4.01E-02	4.69E-02	0.628
C11	Norepinephrine	4.90575	3.17454	4.27E-02	4.94E-02	0.647
B10	Caffeine	2.82448	1.83116	5.82E-02	6.65E-02	0.648
E11	Thyroxine	4.14601	2.59938	6.96E-02	7.86E-02	0.627
C12	Norepinephrine	3.44207	2.29377	7.38E-02	8.23E-02	0.666
A10	Dibutyryl-cAMP	5.41817	3.62125	7.81E-02	8.52E-02	0.668
A11	Dibutyryl-cAMP	4.83553	3.28433	7.81E-02	8.52E-02	0.679
B04	3-Isobutyl-1-Methylxanthine	3.04958	1.82638	9.25E-02	9.98E-02	0.599
B05	3-Isobutyl-1-Methylxanthine	0.98072	1.14871	1.03E-01	1.10E-01	1.171
F12	Hydrocortisone	3.00501	2.10641	1.72E-01	1.82E-01	0.701
A12	Dibutyryl-cAMP	3.3225	2.43362	2.08E-01	2.17E-01	0.732
B11	Caffeine	2.59198	1.89338	3.89E-01	4.02E-01	0.730
B12	Caffeine	1.43671	1.23325	4.19E-01	4.28E-01	0.858
B06	3-Isobutyl-1-Methylxanthine	1.86579	0.89637	5.69E-01	5.75E-01	0.480
E12	Thyroxine	2.24129	1.78934	8.43E-01	8.43E-01	0.798

PMM6_Pre-C-19 Control to LC-19 Official Diagnosis

Well	Well compound	Mean Pre-C-19 Control	Mean LC-19 official Diagnosis	Unadjusted p-value	Adjusted p-value	Fold difference in NADH production
A01	NegativeControl	5.649	1.218	4.67E-03	9.02E-03	0.216
A02	NegativeControl	5.907	1.234	4.14E-03	9.02E-03	0.209
A03	NegativeControl	6.183	1.184	4.14E-03	9.02E-03	0.191
A04	NegativeControl	7.125	1.429	4.67E-03	9.02E-03	0.201
A05	NegativeControl	5.638	1.103	5.92E-03	9.02E-03	0.196
A06	NegativeControl	5.984	1.165	5.92E-03	9.02E-03	0.195
A07	Dibutyryl-cAMP	5.483	1.153	4.67E-03	9.02E-03	0.210
A08	Dibutyryl-cAMP	5.671	1.344	4.14E-03	9.02E-03	0.237
A09	Dibutyryl-cAMP	4.074	1.075	5.26E-03	9.02E-03	0.264
B07	Caffeine	6.890	1.774	5.92E-03	9.02E-03	0.257
B08	Caffeine	4.654	1.309	5.92E-03	9.02E-03	0.281
C01	Epinephrine	5.825	1.435	4.14E-03	9.02E-03	0.246
C02	Epinephrine	6.352	1.413	4.14E-03	9.02E-03	0.223
C03	Epinephrine	6.477	1.467	5.92E-03	9.02E-03	0.226
C04	Epinephrine	7.656	1.720	5.26E-03	9.02E-03	0.225
C05	Epinephrine	4.623	1.039	4.67E-03	9.02E-03	0.225
C06	Epinephrine	5.370	1.174	4.14E-03	9.02E-03	0.219
C07	Norepinephrine	5.197	1.279	4.14E-03	9.02E-03	0.246
C08	Norepinephrine	4.486	1.154	5.26E-03	9.02E-03	0.257
C09	Norepinephrine	4.340	1.176	5.26E-03	9.02E-03	0.271
D01	L-Leucine	5.702	1.384	4.14E-03	9.02E-03	0.243
D02	L-Leucine	7.684	1.743	4.14E-03	9.02E-03	0.227
D03	L-Leucine	7.749	1.794	5.92E-03	9.02E-03	0.232
D04	L-Leucine	6.787	1.692	5.92E-03	9.02E-03	0.249
D05	L-Leucine	3.977	0.985	5.92E-03	9.02E-03	0.248
D06	L-Leucine	3.509	1.140	5.92E-03	9.02E-03	0.325
D07	Creatine	4.760	1.139	4.14E-03	9.02E-03	0.239
D10	Creatine	4.157	1.342	5.92E-03	9.02E-03	0.323
D11	Creatine	4.857	1.740	5.92E-03	9.02E-03	0.358
E01	Triiodothyronine	5.840	1.469	4.14E-03	9.02E-03	0.252
E02	Triiodothyronine	7.448	1.742	4.67E-03	9.02E-03	0.234
E03	Triiodothyronine	6.804	1.467	4.14E-03	9.02E-03	0.216
E04	Triiodothyronine	7.664	1.686	5.26E-03	9.02E-03	0.220
E05	Triiodothyronine	5.458	1.226	5.26E-03	9.02E-03	0.225
E06	Triiodothyronine	5.286	1.180	4.14E-03	9.02E-03	0.223
E07	Thyroxine	5.194	1.284	5.26E-03	9.02E-03	0.247
E08	Thyroxine	6.128	1.670	5.92E-03	9.02E-03	0.273
E09	Thyroxine	3.323	1.039	5.92E-03	9.02E-03	0.313
F01	Dexamethasone	6.797	1.606	4.14E-03	9.02E-03	0.236
F02	Dexamethasone	6.863	1.588	4.14E-03	9.02E-03	0.231
F03	Dexamethasone	6.822	1.588	5.92E-03	9.02E-03	0.233
F04	Dexamethasone	3.992	0.936	4.14E-03	9.02E-03	0.234
F07	Hydrocortisone	4.853	1.087	4.14E-03	9.02E-03	0.224
F08	Hydrocortisone	4.948	1.382	5.92E-03	9.02E-03	0.279
F09	Hydrocortisone	4.885	1.540	5.92E-03	9.02E-03	0.315
G01	Progesterone	5.377	1.401	4.14E-03	9.02E-03	0.261
G02	Progesterone	7.007	1.702	4.14E-03	9.02E-03	0.243
G03	Progesterone	4.612	1.154	4.14E-03	9.02E-03	0.250
G04	Progesterone	7.136	1.599	4.67E-03	9.02E-03	0.224
G05	Progesterone	4.167	0.958	5.26E-03	9.02E-03	0.230
G06	Progesterone	6.980	1.637	4.14E-03	9.02E-03	0.235
G07	beta-Estradiol	6.204	1.671	5.92E-03	9.02E-03	0.269
G08	beta-Estradiol	4.542	1.339	4.67E-03	9.02E-03	0.295
G09	beta-Estradiol	4.801	1.548	4.67E-03	9.02E-03	0.322
G11	beta-Estradiol	4.768	1.623	4.67E-03	9.02E-03	0.340
G12	beta-Estradiol	3.830	1.359	5.92E-03	9.02E-03	0.355

H02	4,5-a-Dihydrotestosterone	7.394	2.053	4.14E-03	9.02E-03	0.278
H03	4,5-a-Dihydrotestosterone	7.148	1.972	5.26E-03	9.02E-03	0.276
H04	4,5-a-Dihydrotestosterone	6.185	1.633	4.67E-03	9.02E-03	0.264
H05	4,5-a-Dihydrotestosterone	7.000	1.953	4.67E-03	9.02E-03	0.279
H06	4,5-a-Dihydrotestosterone	6.537	1.999	4.67E-03	9.02E-03	0.306
H07	Aldosterone	5.428	1.638	5.92E-03	9.02E-03	0.302
H08	Aldosterone	5.127	1.616	4.67E-03	9.02E-03	0.315
A11	Dibutyryl-cAMP	4.836	1.269	6.66E-03	9.13E-03	0.262
B09	Caffeine	5.014	1.656	6.66E-03	9.13E-03	0.330
D08	Creatine	5.625	1.606	6.66E-03	9.13E-03	0.286
E10	Thyroxine	4.426	1.480	6.66E-03	9.13E-03	0.334
F10	Hydrocortisone	3.451	1.224	6.66E-03	9.13E-03	0.355
F11	Hydrocortisone	3.807	1.398	6.66E-03	9.13E-03	0.367
H09	Aldosterone	4.097	1.504	6.66E-03	9.13E-03	0.367
A10	Dibutyryl-cAMP	5.418	1.627	7.47E-03	9.83E-03	0.300
F05	Dexamethasone	4.269	1.079	7.47E-03	9.83E-03	0.253
H01	4,5-a-Dihydrotestosterone	5.457	1.676	7.47E-03	9.83E-03	0.307
C10	Norepinephrine	4.154	1.398	8.37E-03	1.04E-02	0.336
D09	Creatine	5.106	1.571	8.37E-03	1.04E-02	0.308
D12	Creatine	4.027	1.577	8.37E-03	1.04E-02	0.392
F06	Dexamethasone	4.771	1.205	8.37E-03	1.04E-02	0.253
C11	Norepinephrine	4.906	1.826	9.37E-03	1.14E-02	0.372
G10	beta-Estradiol	4.603	1.560	9.37E-03	1.14E-02	0.339
B10	Caffeine	2.824	1.069	1.05E-02	1.24E-02	0.379
H10	Aldosterone	4.848	1.890	1.05E-02	1.24E-02	0.390
B01	3-Isobutyl-1-Methylxanthine	6.385	1.663	1.17E-02	1.37E-02	0.260
B02	3-Isobutyl-1-Methylxanthine	7.554	1.892	1.45E-02	1.68E-02	0.250
H11	Aldosterone	4.528	1.819	1.61E-02	1.85E-02	0.402
E11	Thyroxine	4.146	1.470	1.79E-02	2.03E-02	0.355
B03	3-Isobutyl-1-Methylxanthine	5.744	1.528	1.99E-02	2.22E-02	0.266
C12	Norepinephrine	3.442	1.442	2.20E-02	2.43E-02	0.419
A12	Dibutyryl-cAMP	3.323	0.978	2.69E-02	2.93E-02	0.294
H12	Aldosterone	4.006	1.767	3.59E-02	3.88E-02	0.441
F12	Hydrocortisone	3.005	1.342	4.33E-02	4.62E-02	0.447
B04	3-Isobutyl-1-Methylxanthine	3.050	1.129	5.68E-02	5.99E-02	0.370
B11	Caffeine	2.592	1.358	1.60E-01	1.67E-01	0.524
B05	3-Isobutyl-1-Methylxanthine	0.981	1.088	3.26E-01	3.37E-01	1.109
B12	Caffeine	1.437	1.223	6.03E-01	6.16E-01	0.851
E12	Thyroxine	2.241	1.356	6.58E-01	6.65E-01	0.605
B06	3-Isobutyl-1-Methylxanthine	1.866	0.890	7.15E-01	7.15E-01	0.477

PMM7_Post-C-19 Control to Pre-C-19 Control

Well	Well Compound	Mean Pre-C-19 Control	Mean Post-C-19 Control	Unadjusted p-value	Adjusted p-value	Fold difference in NADH production
A03	NegativeControl	4.90144	3.7256	6.37E-02	3.76E-01	0.760
A05	NegativeControl	6.76239	5.268	6.65E-02	3.76E-01	0.779
B01	Resistin	5.92037	4.6471	6.95E-02	3.76E-01	0.785
B02	Resistin	4.81089	3.7645	6.37E-02	3.76E-01	0.782
B04	Resistin	7.41037	5.8055	7.92E-02	3.76E-01	0.783
B12	Glucagon	4.56515	5.666	7.59E-02	3.76E-01	1.241
C02	Ghrelin	4.79666	3.7995	7.59E-02	3.76E-01	0.792
C05	Ghrelin	6.61624	5.0851	5.31E-02	3.76E-01	0.769
C06	Ghrelin	7.38307	5.7522	8.62E-02	3.76E-01	0.779
D01	Gastrin	6.05677	4.6613	3.82E-02	3.76E-01	0.770
D04	Gastrin	6.4633	4.987	6.65E-02	3.76E-01	0.772
D05	Gastrin	4.85405	3.6427	2.08E-02	3.76E-01	0.750
D06	Gastrin	6.60523	4.4696	1.35E-02	3.76E-01	0.677
D07	Exendin-3	6.40514	4.9731	4.20E-02	3.76E-01	0.776
E03	hGH(Somatotropin)	7.3609	5.6776	3.64E-02	3.76E-01	0.771
E04	hGH(Somatotropin)	6.66386	4.9383	1.88E-02	3.76E-01	0.741
E05	hGH(Somatotropin)	4.47901	3.2586	1.60E-02	3.76E-01	0.728
E06	hGH(Somatotropin)	4.98068	3.45	6.01E-03	3.76E-01	0.693
F04	FGF-1(aFGF)	6.23385	4.8349	7.59E-02	3.76E-01	0.776
F05	FGF-1(aFGF)	7.90533	6.0092	4.01E-02	3.76E-01	0.760
F07	PDGF-AB	4.71016	3.7627	8.26E-02	3.76E-01	0.799
G04	IL-1beta	7.52447	5.696	5.56E-02	3.76E-01	0.757
A02	NegativeControl	5.1383	4.08	9.77E-02	3.78E-01	0.794
E07	IGF-I	5.44121	4.4015	1.02E-01	3.78E-01	0.809
F02	FGF-1(aFGF)	7.84396	6.0854	1.10E-01	3.78E-01	0.776
F06	FGF-1(aFGF)	7.81416	6.3432	9.37E-02	3.78E-01	0.812
G01	IL-1beta	5.11415	4.2464	1.10E-01	3.78E-01	0.830
G06	IL-1beta	6.09258	4.4186	1.10E-01	3.78E-01	0.725
A04	NegativeControl	5.06454	4.0624	1.19E-01	3.95E-01	0.802
C04	Ghrelin	6.38197	4.9481	1.24E-01	3.98E-01	0.775
A06	NegativeControl	6.09062	4.8207	1.29E-01	4.00E-01	0.791
B11	Glucagon	4.21464	5.0327	1.39E-01	4.06E-01	1.194
D02	Gastrin	5.14458	4.237	1.39E-01	4.06E-01	0.824
H04	IL-6	5.62322	4.5867	1.45E-01	4.09E-01	0.816
H01	IL-6	5.50475	4.5078	1.50E-01	4.13E-01	0.819
D03	Gastrin	6.4365	5.3106	1.56E-01	4.16E-01	0.825
C01	Ghrelin	5.92672	4.9208	1.62E-01	4.20E-01	0.830
E01	hGH(Somatotropin)	4.74702	3.9296	1.74E-01	4.29E-01	0.828
G05	IL-1beta	7.95125	6.4514	1.74E-01	4.29E-01	0.811
C03	Ghrelin	5.85501	4.8216	1.81E-01	4.33E-01	0.823
B07	Glucagon	5.95829	4.9399	1.94E-01	4.54E-01	0.829
A01	NegativeControl	5.14678	4.2694	2.01E-01	4.59E-01	0.830
B05	Resistin	5.05531	4.1416	2.23E-01	4.64E-01	0.819
F01	FGF-1(aFGF)	5.94069	4.9533	2.23E-01	4.64E-01	0.834
F03	FGF-1(aFGF)	8.33372	6.9613	2.15E-01	4.64E-01	0.835
H08	IL-8	4.5062	3.8193	2.15E-01	4.64E-01	0.848
H05	IL-6	5.52751	4.719	2.30E-01	4.70E-01	0.854
B03	Resistin	7.6193	6.4164	2.46E-01	4.72E-01	0.842
C12	Leptin	4.26935	4.8173	2.46E-01	4.72E-01	1.128
G07	IL-2	7.24591	6.0211	2.38E-01	4.72E-01	0.831
C11	Leptin	5.07094	5.6784	2.62E-01	4.73E-01	1.120
F08	PDGF-AB	7.30249	6.1042	2.54E-01	4.73E-01	0.836
G03	IL-1beta	7.85544	6.5357	2.62E-01	4.73E-01	0.832
H06	IL-6	5.94409	5.0823	2.71E-01	4.73E-01	0.855
H07	IL-8	5.96425	5.0315	2.71E-01	4.73E-01	0.844
A12	Insulin	3.48844	3.982	2.89E-01	4.95E-01	1.141
H03	IL-6	5.56743	4.7995	3.07E-01	5.17E-01	0.862
C10	Leptin	5.89552	6.4283	3.16E-01	5.22E-01	1.090
E02	hGH(Somatotropin)	7.92334	6.7936	3.26E-01	5.22E-01	0.857
E12	IGF-I	4.18656	4.5546	3.26E-01	5.22E-01	1.088

A11	Insulin	3.88037	4.2722	3.67E-01	5.77E-01	1.101
A09	Insulin	3.23996	3.4909	3.77E-01	5.84E-01	1.077
A10	Insulin	3.11838	3.4198	3.88E-01	5.92E-01	1.097
C07	Leptin	7.18505	6.1749	4.10E-01	6.16E-01	0.859
B09	Glucagon	5.31598	5.624	4.33E-01	6.21E-01	1.058
F11	PDGF-AB	5.78275	4.6876	4.33E-01	6.21E-01	0.811
G02	IL-1beta	7.40573	6.5399	4.33E-01	6.21E-01	0.883
H02	IL-6	4.64788	4.1789	4.57E-01	6.45E-01	0.899
E10	IGF-I	5.8049	6.2084	4.81E-01	6.70E-01	1.070
D08	Exendin-3	5.02829	4.4351	5.06E-01	6.85E-01	0.882
F10	PDGF-AB	5.49713	5.6479	5.06E-01	6.85E-01	1.027
A07	Insulin	5.02413	4.3827	5.32E-01	6.90E-01	0.872
B06	Resistin	6.91427	6.083	5.32E-01	6.90E-01	0.880
E08	IGF-I	6.80794	6.1717	5.32E-01	6.90E-01	0.907
D10	Exendin-3	4.7185	4.9185	5.45E-01	6.96E-01	1.042
D12	Exendin-3	5.24673	5.5471	5.58E-01	6.96E-01	1.057
E09	IGF-I	5.38767	5.5259	5.58E-01	6.96E-01	1.026
D11	Exendin-3	4.87217	5.0615	6.13E-01	7.54E-01	1.039
C09	Leptin	3.90426	3.9382	6.99E-01	8.28E-01	1.009
G08	IL-2	4.91022	4.4846	6.99E-01	8.28E-01	0.913
H10	IL-8	3.90959	3.9554	6.99E-01	8.28E-01	1.012
G11	IL-2	4.60498	4.6522	7.14E-01	8.36E-01	1.010
B10	Glucagon	3.84487	3.9658	7.43E-01	8.50E-01	1.031
G12	IL-2	4.69797	4.875	7.43E-01	8.50E-01	1.038
E11	IGF-I	4.59369	4.6466	7.89E-01	8.91E-01	1.012
F12	PDGF-AB	4.7518	4.7821	8.35E-01	9.32E-01	1.006
C08	Leptin	5.17193	4.81	8.66E-01	9.45E-01	0.930
H11	IL-8	3.90641	3.4522	8.66E-01	9.45E-01	0.884
B08	Glucagon	4.98954	4.719	8.97E-01	9.57E-01	0.946
F09	PDGF-AB	7.26154	6.7615	8.97E-01	9.57E-01	0.931
A08	Insulin	4.47747	4.2495	9.60E-01	9.60E-01	0.949
D09	Exendin-3	5.79135	5.591	9.29E-01	9.60E-01	0.965
G09	IL-2	5.48638	5.1993	9.45E-01	9.60E-01	0.948
G10	IL-2	6.42624	6.3609	9.60E-01	9.60E-01	0.990
H09	IL-8	4.24917	4.1052	9.29E-01	9.60E-01	0.966
H12	IL-8	4.84935	4.5203	9.60E-01	9.60E-01	0.932

PMM7_Post-C-19 Control to LC-19

Well	Well Compound	Mean Post-C-19 Control	Mean LC-19	Unadjusted p-value	Adjusted p-value	Fold difference in NADH production
A01	NegativeControl	4.2694	2.6508	5.24E-02	2.51E-01	0.621
A02	NegativeControl	4.08	1.8851	8.93E-03	2.51E-01	0.462
A03	NegativeControl	3.7256	2.0904	4.33E-02	2.51E-01	0.561
A04	NegativeControl	4.0624	2.2507	3.55E-02	2.51E-01	0.554
A05	NegativeControl	5.268	2.8506	4.33E-02	2.51E-01	0.541
A06	NegativeControl	4.8207	2.6345	3.76E-02	2.51E-01	0.546
A07	Insulin	4.3827	2.277	4.33E-02	2.51E-01	0.520
A08	Insulin	4.2495	2.0774	2.32E-02	2.51E-01	0.489
A09	Insulin	3.4909	1.9801	3.55E-02	2.51E-01	0.567
A10	Insulin	3.4198	1.9375	3.55E-02	2.51E-01	0.567
A11	Insulin	4.2722	2.4356	3.55E-02	2.51E-01	0.570
A12	Insulin	3.982	2.1927	2.88E-02	2.51E-01	0.551
B03	Resistin	6.4164	4.5706	1.23E-01	2.51E-01	0.712
B12	Glucagon	5.666	3.8131	5.24E-02	2.51E-01	0.673
C02	Ghrelin	3.7995	2.8957	1.23E-01	2.51E-01	0.762
C03	Ghrelin	4.8216	3.5752	1.05E-01	2.51E-01	0.741
C06	Ghrelin	5.7522	4.3176	6.30E-02	2.51E-01	0.751
D03	Gastrin	5.3106	3.8229	8.92E-02	2.51E-01	0.720
D04	Gastrin	4.987	3.6067	1.05E-01	2.51E-01	0.723
D05	Gastrin	3.6427	2.7514	1.23E-01	2.51E-01	0.755
D08	Exendin-3	4.4351	3.2361	8.92E-02	2.51E-01	0.730
E08	IGF-I	6.1717	4.5094	1.23E-01	2.51E-01	0.731
E09	IGF-I	5.5259	3.9334	5.24E-02	2.51E-01	0.712
F01	FGF-1(aFGF)	4.9533	3.6713	1.05E-01	2.51E-01	0.741
F03	FGF-1(aFGF)	6.9613	4.8866	6.30E-02	2.51E-01	0.702
F04	FGF-1(aFGF)	4.8349	3.4684	1.23E-01	2.51E-01	0.717
F05	FGF-1(aFGF)	6.0092	4.3884	7.53E-02	2.51E-01	0.730
F06	FGF-1(aFGF)	6.3432	4.5615	6.30E-02	2.51E-01	0.719
F07	PDGF-AB	3.7627	2.7301	7.53E-02	2.51E-01	0.726
F09	PDGF-AB	6.7615	5.0673	8.89E-02	2.51E-01	0.749
F10	PDGF-AB	5.6479	4.1117	6.30E-02	2.51E-01	0.728
G01	IL-1beta	4.2464	2.9366	5.24E-02	2.51E-01	0.692
G02	IL-1beta	6.5399	4.8025	1.05E-01	2.51E-01	0.734
G03	IL-1beta	6.5357	4.7298	8.92E-02	2.51E-01	0.724
G04	IL-1beta	5.696	4.162	1.05E-01	2.51E-01	0.731
G05	IL-1beta	6.4514	4.2803	6.30E-02	2.51E-01	0.663
G08	IL-2	4.4846	2.8618	7.53E-02	2.51E-01	0.638
G09	IL-2	5.1993	3.6876	1.23E-01	2.51E-01	0.709
G10	IL-2	6.3609	4.7749	1.05E-01	2.51E-01	0.751
H02	IL-6	4.1789	2.9976	7.53E-02	2.51E-01	0.717
H03	IL-6	4.7995	3.4275	8.92E-02	2.51E-01	0.714
H04	IL-6	4.5867	3.193	6.30E-02	2.51E-01	0.696
H05	IL-6	4.719	3.423	7.53E-02	2.51E-01	0.725
H06	IL-6	5.0823	3.7287	6.30E-02	2.51E-01	0.734
H08	IL-8	3.8193	2.8349	1.23E-01	2.51E-01	0.742
H09	IL-8	4.1052	3.1481	1.23E-01	2.51E-01	0.767
H10	IL-8	3.9554	3.0546	1.23E-01	2.51E-01	0.772
B06	Resistin	6.083	4.4217	1.43E-01	2.75E-01	0.727
C04	Ghrelin	4.9481	3.7286	1.43E-01	2.75E-01	0.754
C09	Leptin	3.9382	3.0166	1.43E-01	2.75E-01	0.766
B04	Resistin	5.8055	4.4854	1.65E-01	2.79E-01	0.773
B05	Resistin	4.1416	3.0967	1.65E-01	2.79E-01	0.748
C05	Ghrelin	5.0851	3.9034	1.65E-01	2.79E-01	0.768
C07	Leptin	6.1749	4.8847	1.65E-01	2.79E-01	0.791
D02	Gastrin	4.237	3.1864	1.65E-01	2.79E-01	0.752
D07	Exendin-3	4.9731	3.7952	1.65E-01	2.79E-01	0.763
F08	PDGF-AB	6.1042	4.5485	1.65E-01	2.79E-01	0.745
B01	Resistin	4.6471	3.5641	1.90E-01	3.05E-01	0.767
E10	IGF-I	6.2084	4.7677	1.90E-01	3.05E-01	0.768
H01	IL-6	4.5078	3.3215	1.90E-01	3.05E-01	0.737
B10	Glucagon	3.9658	3.0562	2.18E-01	3.12E-01	0.771

C08	Leptin	4.81	3.8095	2.18E-01	3.12E-01	0.792
D06	Gastrin	4.4696	3.7727	2.18E-01	3.12E-01	0.844
D11	Exendin-3	5.0615	3.8766	2.18E-01	3.12E-01	0.766
E12	IGF-I	4.5546	3.6171	2.18E-01	3.12E-01	0.794
G07	IL-2	6.0211	4.445	2.18E-01	3.12E-01	0.738
H07	IL-8	5.0315	3.9627	2.18E-01	3.12E-01	0.788
B02	Resistin	3.7645	2.9444	2.47E-01	3.35E-01	0.782
B07	Glucagon	4.9399	3.8895	2.47E-01	3.35E-01	0.787
B09	Glucagon	5.624	4.5562	2.47E-01	3.35E-01	0.810
B11	Glucagon	5.0327	4.0209	2.47E-01	3.35E-01	0.799
C01	Ghrelin	4.9208	3.9537	2.80E-01	3.68E-01	0.803
D01	Gastrin	4.6613	3.5933	2.80E-01	3.68E-01	0.771
C12	Leptin	4.8173	3.9251	3.15E-01	3.88E-01	0.815
D09	Exendin-3	5.591	4.4334	3.15E-01	3.88E-01	0.793
F02	FGF-1(aFGF)	6.0854	4.8667	3.15E-01	3.88E-01	0.800
G06	IL-1beta	4.4186	3.6958	3.15E-01	3.88E-01	0.836
G11	IL-2	4.6522	3.6252	3.15E-01	3.88E-01	0.779
B08	Glucagon	4.719	3.8937	3.53E-01	4.08E-01	0.825
E01	hGH(Somatotropin)	3.9296	3.177	3.53E-01	4.08E-01	0.808
E02	hGH(Somatotropin)	6.7936	5.5834	3.53E-01	4.08E-01	0.822
E07	IGF-I	4.4015	3.4558	3.53E-01	4.08E-01	0.785
E11	IGF-I	4.6466	3.7809	3.53E-01	4.08E-01	0.814
C10	Leptin	6.4283	5.4562	3.93E-01	4.34E-01	0.849
D10	Exendin-3	4.9185	3.9624	3.93E-01	4.34E-01	0.806
F12	PDGF-AB	4.7821	3.9564	3.93E-01	4.34E-01	0.827
H11	IL-8	3.4522	3.116	3.93E-01	4.34E-01	0.903
C11	Leptin	5.6784	4.6719	4.36E-01	4.76E-01	0.823
D12	Exendin-3	5.5471	4.7602	5.29E-01	5.70E-01	0.858
E03	hGH(Somatotropin)	5.6776	5.0457	5.79E-01	6.04E-01	0.889
F11	PDGF-AB	4.6876	4.3455	5.79E-01	6.04E-01	0.927
G12	IL-2	4.875	4.3014	5.79E-01	6.04E-01	0.882
H12	IL-8	4.5203	4.3404	7.39E-01	7.63E-01	0.960
E04	hGH(Somatotropin)	4.9383	4.7771	7.96E-01	8.13E-01	0.967
E05	hGH(Somatotropin)	3.2586	3.2691	9.12E-01	9.21E-01	1.003
E06	hGH(Somatotropin)	3.45	3.4201	9.71E-01	9.71E-01	0.991

PMM7_Post-C-19 Control to LC-19 more than 4 symptoms

Well	CMS#	Mean Post-C-19 Control	Mean LC-19 >4 symptoms	Unadjusted p-value	Adjusted p-value	Fold difference in NADH production
A01	NegativeControl	4.269	2.399	9.34E-02	2.51E-01	0.562
A02	NegativeControl	4.080	1.738	1.60E-02	2.51E-01	0.426
A03	NegativeControl	3.726	2.067	7.27E-02	2.51E-01	0.555
A04	NegativeControl	4.062	2.195	5.59E-02	2.51E-01	0.540
A05	NegativeControl	5.268	2.842	9.34E-02	2.51E-01	0.539
A06	NegativeControl	4.821	2.712	9.34E-02	2.51E-01	0.563
A07	Insulin	4.383	2.280	9.34E-02	2.51E-01	0.520
A08	Insulin	4.250	2.290	9.34E-02	2.51E-01	0.539
A09	Insulin	3.491	2.006	9.34E-02	2.51E-01	0.574
A10	Insulin	3.420	2.052	1.18E-01	2.51E-01	0.600
A11	Insulin	4.272	2.544	1.18E-01	2.51E-01	0.596
A12	Insulin	3.982	2.210	7.27E-02	2.51E-01	0.555
B01	Resistin	4.647	2.982	1.18E-01	2.51E-01	0.642
B02	Resistin	3.765	2.499	9.34E-02	2.57E-01	0.664
B03	Resistin	6.416	3.856	1.18E-01	2.51E-01	0.601
B04	Resistin	5.806	3.804	5.59E-02	2.57E-01	0.655
B05	Resistin	4.142	2.641	9.34E-02	2.57E-01	0.638
B06	Resistin	6.083	3.851	9.34E-02	2.51E-01	0.633
B07	Glucagon	4.940	3.374	7.27E-02	2.97E-01	0.683
B08	Glucagon	4.719	3.448	1.18E-01	3.67E-01	0.731
B09	Glucagon	5.624	4.026	7.27E-02	2.97E-01	0.716
B10	Glucagon	3.966	2.788	9.34E-02	2.97E-01	0.703
B11	Glucagon	5.033	3.648	9.34E-02	2.97E-01	0.725
B12	Glucagon	5.666	3.209	1.18E-01	2.51E-01	0.566
C01	Ghrelin	4.921	3.460	7.27E-02	2.97E-01	0.703
C02	Ghrelin	3.800	2.492	1.18E-01	2.51E-01	0.656
C03	Ghrelin	4.822	3.038	7.27E-02	2.51E-01	0.630
C04	Ghrelin	4.948	3.133	9.34E-02	2.57E-01	0.633
C05	Ghrelin	5.085	3.336	7.27E-02	2.57E-01	0.656
C06	Ghrelin	5.752	3.863	7.27E-02	2.51E-01	0.672
C07	Leptin	6.175	4.310	9.34E-02	2.57E-01	0.698
C08	Leptin	4.810	3.427	1.18E-01	3.33E-01	0.712
C09	Leptin	3.938	2.738	1.16E-01	2.80E-01	0.695
C10	Leptin	6.428	4.884	7.27E-02	4.06E-01	0.760
C11	Leptin	5.678	4.223	7.27E-02	5.08E-01	0.744
C12	Leptin	4.817	3.548	1.18E-01	3.33E-01	0.737
D01	Gastrin	4.661	3.136	9.34E-02	2.80E-01	0.673
D02	Gastrin	4.237	2.719	9.34E-02	2.51E-01	0.642
D03	Gastrin	5.311	3.291	9.34E-02	2.51E-01	0.620
D04	Gastrin	4.987	3.092	7.27E-02	2.51E-01	0.620
D05	Gastrin	3.643	2.381	9.34E-02	2.51E-01	0.654
D06	Gastrin	4.470	3.344	7.27E-02	2.97E-01	0.748
D07	Exendin-3	4.973	3.332	7.27E-02	2.80E-01	0.670
D08	Exendin-3	4.435	2.840	7.27E-02	2.51E-01	0.640
D09	Exendin-3	5.591	3.953	1.18E-01	3.67E-01	0.707
D10	Exendin-3	4.919	3.560	1.47E-01	4.06E-01	0.724
D11	Exendin-3	5.062	3.508	1.47E-01	2.97E-01	0.693
D12	Exendin-3	5.547	4.305	1.47E-01	4.61E-01	0.776
E01	hGH(Somatotropin)	3.930	2.819	1.47E-01	3.33E-01	0.717
E02	hGH(Somatotropin)	6.794	4.729	1.47E-01	2.97E-01	0.696
E03	hGH(Somatotropin)	5.678	4.360	1.47E-01	3.67E-01	0.768
E04	hGH(Somatotropin)	4.938	4.196	1.47E-01	5.08E-01	0.850
E05	hGH(Somatotropin)	3.259	2.858	1.47E-01	5.08E-01	0.877
E06	hGH(Somatotropin)	3.450	2.943	1.47E-01	5.08E-01	0.853
E07	IGF-I	4.402	3.104	1.47E-01	4.06E-01	0.705
E08	IGF-I	6.172	4.112	1.81E-01	2.80E-01	0.666

E09	IGF-I	5.526	3.559	1.81E-01	2.51E-01	0.644
E10	IGF-I	6.208	4.434	1.81E-01	2.97E-01	0.714
E11	IGF-I	4.647	3.458	1.81E-01	4.61E-01	0.744
E12	IGF-I	4.555	3.352	1.81E-01	3.67E-01	0.736
F01	FGF-1(aFGF)	4.953	3.279	1.81E-01	2.51E-01	0.662
F02	FGF-1(aFGF)	6.085	4.177	1.81E-01	3.33E-01	0.686
F03	FGF-1(aFGF)	6.961	4.163	2.20E-01	2.51E-01	0.598
F04	FGF-1(aFGF)	4.835	2.990	2.20E-01	2.51E-01	0.618
F05	FGF-1(aFGF)	6.009	3.698	2.20E-01	2.51E-01	0.615
F06	FGF-1(aFGF)	6.343	4.050	2.20E-01	2.51E-01	0.639
F07	PDGF-AB	3.763	2.470	2.20E-01	2.51E-01	0.656
F08	PDGF-AB	6.104	3.896	2.20E-01	2.51E-01	0.638
F09	PDGF-AB	6.762	4.651	2.20E-01	2.51E-01	0.688
F10	PDGF-AB	5.648	3.709	2.20E-01	2.51E-01	0.657
F11	PDGF-AB	4.688	4.041	2.20E-01	5.68E-01	0.862
F12	PDGF-AB	4.782	3.570	2.63E-01	4.06E-01	0.746
G01	IL-1beta	4.246	2.628	2.63E-01	2.51E-01	0.619
G02	IL-1beta	6.540	4.260	2.63E-01	2.51E-01	0.651
G03	IL-1beta	6.536	4.198	2.63E-01	2.51E-01	0.642
G04	IL-1beta	5.696	3.785	2.63E-01	2.57E-01	0.665
G05	IL-1beta	6.451	4.170	3.13E-01	2.51E-01	0.646
G06	IL-1beta	4.419	3.345	3.13E-01	3.67E-01	0.757
G07	IL-2	6.021	4.121	3.13E-01	3.33E-01	0.684
G08	IL-2	4.485	2.559	3.13E-01	2.51E-01	0.571
G09	IL-2	5.199	3.519	3.13E-01	2.80E-01	0.677
G10	IL-2	6.361	4.444	3.13E-01	2.80E-01	0.699
G11	IL-2	4.652	3.335	3.68E-01	3.67E-01	0.717
G12	IL-2	4.875	4.012	3.68E-01	5.68E-01	0.823
H01	IL-6	4.508	3.050	3.68E-01	2.57E-01	0.677
H02	IL-6	4.179	2.653	3.68E-01	2.51E-01	0.635
H03	IL-6	4.800	3.026	3.68E-01	2.51E-01	0.630
H04	IL-6	4.587	2.785	4.28E-01	2.51E-01	0.607
H05	IL-6	4.719	3.006	4.28E-01	2.51E-01	0.637
H06	IL-6	5.082	3.304	4.92E-01	2.51E-01	0.650
H07	IL-8	5.032	3.604	4.92E-01	2.80E-01	0.716
H08	IL-8	3.819	2.562	4.92E-01	2.51E-01	0.671
H09	IL-8	4.105	2.882	4.92E-01	2.57E-01	0.702
H10	IL-8	3.955	2.780	5.62E-01	2.57E-01	0.703
H11	IL-8	3.452	2.875	5.62E-01	4.06E-01	0.833
H12	IL-8	4.520	3.997	0.63536	6.35E-01	0.884

PMM7_Post-C-19 Control to LC-19 Official Diagnosis

Well	Well compound	Mean Post-C-19 Control	Mean LC-19 official Diagnosis	Unadjusted p-value	Adjusted p-value	Fold difference in NADH production
A01	NegativeContro	4.269	1.778	1.12E-01	3.09E-01	0.416
A02	NegativeContro	4.080	1.523	4.90E-02	3.09E-01	0.373
A03	NegativeContro	3.726	1.457	1.12E-01	3.09E-01	0.391
A04	NegativeContro	4.062	1.439	4.90E-02	3.09E-01	0.354
A05	NegativeContro	5.268	1.711	4.90E-02	3.09E-01	0.325
A06	NegativeContro	4.821	1.594	4.90E-02	3.09E-01	0.331
A07	Insulin	4.383	1.230	4.90E-02	3.09E-01	0.281
A08	Insulin	4.250	1.356	4.90E-02	3.09E-01	0.319
A09	Insulin	3.491	1.216	4.90E-02	3.09E-01	0.348
A10	Insulin	3.420	1.321	7.69E-02	3.09E-01	0.386
A11	Insulin	4.272	1.716	7.69E-02	3.09E-01	0.402
A12	Insulin	3.982	1.078	2.80E-02	3.09E-01	0.271
B01	Resistin	4.647	2.793	1.61E-01	3.09E-01	0.601
B03	Resistin	6.416	3.453	1.61E-01	3.09E-01	0.538
B10	Glucagon	3.966	2.208	1.61E-01	3.09E-01	0.557
B12	Glucagon	5.666	3.296	1.61E-01	3.09E-01	0.582
C02	Ghrelin	3.800	2.219	1.61E-01	3.09E-01	0.584
C03	Ghrelin	4.822	2.796	1.61E-01	3.09E-01	0.580
C06	Ghrelin	5.752	3.432	1.61E-01	3.09E-01	0.597
C07	Leptin	6.175	3.717	1.61E-01	3.09E-01	0.602
D03	Gastrin	5.311	2.897	1.12E-01	3.09E-01	0.546
D04	Gastrin	4.987	2.679	1.61E-01	3.09E-01	0.537
D05	Gastrin	3.643	2.097	1.61E-01	3.09E-01	0.576
D08	Exendin-3	4.435	2.328	1.12E-01	3.09E-01	0.525
E08	IGF-I	6.172	3.296	1.12E-01	3.09E-01	0.534
E09	IGF-I	5.526	3.155	1.12E-01	3.09E-01	0.571
F03	FGF-1(aFGF)	6.961	3.517	1.12E-01	3.09E-01	0.505
F04	FGF-1(aFGF)	4.835	2.500	1.61E-01	3.09E-01	0.517
F05	FGF-1(aFGF)	6.009	3.052	1.12E-01	3.09E-01	0.508
F06	FGF-1(aFGF)	6.343	3.501	1.12E-01	3.09E-01	0.552
F07	PDGF-AB	3.763	2.054	1.61E-01	3.09E-01	0.546
F09	PDGF-AB	6.762	4.024	1.61E-01	3.09E-01	0.595
F10	PDGF-AB	5.648	3.159	1.12E-01	3.09E-01	0.559
G01	IL-1beta	4.246	2.321	1.12E-01	3.09E-01	0.547
G02	IL-1beta	6.540	3.759	1.61E-01	3.09E-01	0.575
G03	IL-1beta	6.536	3.555	1.12E-01	3.09E-01	0.544
G04	IL-1beta	5.696	3.023	1.12E-01	3.09E-01	0.531
G05	IL-1beta	6.451	3.449	1.12E-01	3.09E-01	0.535
G07	IL-2	6.021	3.285	1.61E-01	3.09E-01	0.546
G08	IL-2	4.485	2.211	1.12E-01	3.09E-01	0.493
G09	IL-2	5.199	2.818	1.61E-01	3.09E-01	0.542
G10	IL-2	6.361	3.794	1.12E-01	3.09E-01	0.596
H01	IL-6	4.508	2.554	1.61E-01	3.09E-01	0.567
H02	IL-6	4.179	2.244	1.12E-01	3.09E-01	0.537
H03	IL-6	4.800	2.590	1.12E-01	3.09E-01	0.540
H04	IL-6	4.587	2.537	1.12E-01	3.09E-01	0.553
H05	IL-6	4.719	2.587	1.12E-01	3.09E-01	0.548
H06	IL-6	5.082	2.949	1.12E-01	3.09E-01	0.580
H08	IL-8	3.819	2.068	1.12E-01	3.09E-01	0.541
H10	IL-8	3.955	2.438	1.61E-01	3.09E-01	0.616
B06	Resistin	6.083	3.367	2.17E-01	3.47E-01	0.554
C04	Ghrelin	4.948	2.938	2.17E-01	3.47E-01	0.594
C05	Ghrelin	5.085	3.017	2.17E-01	3.47E-01	0.593
C09	Leptin	3.938	2.330	2.17E-01	3.47E-01	0.592
D02	Gastrin	4.237	2.561	2.17E-01	3.47E-01	0.605
E12	IGF-I	4.555	2.818	2.17E-01	3.47E-01	0.619

F01	FGF-1(aFGF)	4.953	3.014	2.17E-01	3.47E-01	0.608
F08	PDGF-AB	6.104	3.872	2.17E-01	3.47E-01	0.634
H07	IL-8	5.032	3.082	2.17E-01	3.47E-01	0.613
H09	IL-8	4.105	2.577	2.17E-01	3.47E-01	0.628
B02	Resistin	3.765	2.280	2.87E-01	3.88E-01	0.606
B04	Resistin	5.806	3.379	2.87E-01	3.88E-01	0.582
B05	Resistin	4.142	2.314	2.87E-01	3.88E-01	0.559
B07	Glucagon	4.940	2.978	2.87E-01	3.88E-01	0.603
C08	Leptin	4.810	2.944	2.87E-01	3.88E-01	0.612
D06	Gastrin	4.470	2.978	2.87E-01	3.88E-01	0.666
D07	Exendin-3	4.973	2.997	2.87E-01	3.88E-01	0.603
D11	Exendin-3	5.062	3.005	2.87E-01	3.88E-01	0.594
E10	IGF-I	6.208	3.894	2.87E-01	3.88E-01	0.627
F02	FGF-1(aFGF)	6.085	3.785	2.87E-01	3.88E-01	0.622
G06	IL-1beta	4.419	2.767	2.87E-01	3.88E-01	0.626
B08	Glucagon	4.719	3.031	3.71E-01	4.56E-01	0.642
C01	Ghrelin	4.921	3.201	3.71E-01	4.56E-01	0.650
D01	Gastrin	4.661	2.975	3.71E-01	4.56E-01	0.638
E01	hGH(Somatotro	3.930	2.594	3.71E-01	4.56E-01	0.660
E11	IGF-I	4.647	2.938	3.71E-01	4.56E-01	0.632
G11	IL-2	4.652	2.912	3.71E-01	4.56E-01	0.626
H11	IL-8	3.452	2.510	3.71E-01	4.56E-01	0.727
B09	Glucagon	5.624	3.726	4.69E-01	5.23E-01	0.663
B11	Glucagon	5.033	3.301	4.69E-01	5.23E-01	0.656
C11	Leptin	5.678	3.727	4.69E-01	5.23E-01	0.656
C12	Leptin	4.817	3.216	4.69E-01	5.23E-01	0.668
D09	Exendin-3	5.591	3.590	4.69E-01	5.23E-01	0.642
D12	Exendin-3	5.547	3.874	4.69E-01	5.23E-01	0.698
E02	hGH(Somatotro	6.794	4.664	4.69E-01	5.23E-01	0.686
E07	IGF-I	4.402	2.752	4.69E-01	5.23E-01	0.625
C10	Leptin	6.428	4.543	5.73E-01	5.92E-01	0.707
D10	Exendin-3	4.919	3.282	5.73E-01	5.92E-01	0.667
E03	hGH(Somatotro	5.678	4.286	5.73E-01	5.92E-01	0.755
F11	PDGF-AB	4.688	3.522	5.73E-01	5.92E-01	0.751
F12	PDGF-AB	4.782	3.312	5.73E-01	5.92E-01	0.693
G12	IL-2	4.875	3.721	5.73E-01	5.92E-01	0.763
H12	IL-8	4.520	3.543	5.73E-01	5.92E-01	0.784
E04	hGH(Somatotro	4.938	4.076	8.11E-01	8.11E-01	0.825
E05	hGH(Somatotro	3.259	2.862	8.11E-01	8.11E-01	0.878
E06	hGH(Somatotro	3.450	3.028	8.11E-01	8.11E-01	0.878

PMM7_Pre-C-19 Control to LC-19

Well	Well Compound	Mean Pre-C-19 Control	Mean LC-19	Unadjusted p-value	Adjusted p-value	Fold difference in NADH production
A02	NegativeControl	5.1383	1.8851	0.00E+00	3.20E-04	0.367
A03	NegativeControl	4.90144	2.0904	1e-05	3.70E-04	0.426
A05	NegativeControl	6.76239	2.8506	2e-05	5.60E-04	0.422
A04	NegativeControl	5.06454	2.2507	3e-05	6.50E-04	0.444
A01	NegativeControl	5.14678	2.6508	8e-05	1.13E-03	0.515
A06	NegativeControl	6.09062	2.6345	6e-05	1.13E-03	0.433
G01	IL-1beta	5.11415	2.9366	8e-05	1.13E-03	0.574
A07	Insulin	5.02413	2.277	1.20E-04	1.33E-03	0.453
A08	Insulin	4.47747	2.0774	2.20E-04	1.33E-03	0.464
B04	Resistin	7.41037	4.4854	2e-04	1.33E-03	0.605
C02	Ghrelin	4.79666	2.8957	2.50E-04	1.33E-03	0.604
C04	Ghrelin	6.38197	3.7286	2.50E-04	1.33E-03	0.584
C05	Ghrelin	6.61624	3.9034	3.20E-04	1.33E-03	0.590
D04	Gastrin	6.4633	3.6067	2.90E-04	1.33E-03	0.558
D05	Gastrin	4.85405	2.7514	1.60E-04	1.33E-03	0.567
D06	Gastrin	6.60523	3.7727	2.90E-04	1.33E-03	0.571
F01	FGF-1(aFGF)	5.94069	3.6713	3.20E-04	1.33E-03	0.618
F04	FGF-1(aFGF)	6.23385	3.4684	1.80E-04	1.33E-03	0.556
F05	FGF-1(aFGF)	7.90533	4.3884	3.20E-04	1.33E-03	0.555
F07	PDGF-AB	4.71016	2.7301	1.50E-04	1.33E-03	0.580
G04	IL-1beta	7.52447	4.162	3.20E-04	1.33E-03	0.553
G05	IL-1beta	7.95125	4.2803	1.70E-04	1.33E-03	0.538
H04	IL-6	5.62322	3.193	2.50E-04	1.33E-03	0.568
B03	Resistin	7.6193	4.5706	3.70E-04	1.42E-03	0.600
D01	Gastrin	6.05677	3.5933	3.70E-04	1.42E-03	0.593
B01	Resistin	5.92037	3.5641	4.60E-04	1.48E-03	0.602
B05	Resistin	5.05531	3.0967	4.60E-04	1.48E-03	0.613
C06	Ghrelin	7.38307	4.3176	4.60E-04	1.48E-03	0.585
D07	Exendin-3	6.40514	3.7952	4.30E-04	1.48E-03	0.593
H01	IL-6	5.50475	3.3215	4.30E-04	1.48E-03	0.603
G08	IL-2	4.91022	2.8618	5e-04	1.55E-03	0.583
F03	FGF-1(aFGF)	8.33372	4.8866	5.80E-04	1.74E-03	0.586
H08	IL-8	4.5062	2.8349	6.70E-04	1.95E-03	0.629
B06	Resistin	6.91427	4.4217	7.20E-04	1.97E-03	0.640
C03	Ghrelin	5.85501	3.5752	7.20E-04	1.97E-03	0.611
D03	Gastrin	6.4365	3.8229	7.70E-04	2.06E-03	0.594
H03	IL-6	5.56743	3.4275	8.90E-04	2.32E-03	0.616
F02	FGF-1(aFGF)	7.84396	4.8667	9.60E-04	2.36E-03	0.620
F06	FGF-1(aFGF)	7.81416	4.5615	9.60E-04	2.36E-03	0.584
G03	IL-1beta	7.85544	4.7298	1.03E-03	2.41E-03	0.602
G07	IL-2	7.24591	4.445	1.03E-03	2.41E-03	0.613
E07	IGF-I	5.44121	3.4558	1.27E-03	2.83E-03	0.635
G06	IL-1beta	6.09258	3.6958	1.27E-03	2.83E-03	0.607
B02	Resistin	4.81089	2.9444	1.36E-03	2.96E-03	0.612
D02	Gastrin	5.14458	3.1864	2.04E-03	4.17E-03	0.619
F08	PDGF-AB	7.30249	4.5485	2.04E-03	4.17E-03	0.623
H06	IL-6	5.94409	3.7287	2.04E-03	4.17E-03	0.627
H02	IL-6	4.64788	2.9976	2.18E-03	4.36E-03	0.645
A10	Insulin	3.11838	1.9375	2.49E-03	4.59E-03	0.621
D08	Exendin-3	5.02829	3.2361	2.49E-03	4.59E-03	0.644
G09	IL-2	5.48638	3.6876	2.49E-03	4.59E-03	0.672
H05	IL-6	5.52751	3.423	2.49E-03	4.59E-03	0.619
C01	Ghrelin	5.92672	3.9537	2.66E-03	4.81E-03	0.667
B07	Glucagon	5.95829	3.8895	3.02E-03	5.37E-03	0.653
E08	IGF-I	6.80794	4.5094	3.44E-03	6.00E-03	0.662
A09	Insulin	3.23996	1.9801	4.71E-03	7.92E-03	0.611

H07	IL-8	5.96425	3.9627	4.71E-03	7.92E-03	0.664
A11	Insulin	3.88037	2.4356	5.01E-03	8.28E-03	0.628
E01	hGH(Somatotropin)	4.74702	3.177	6.38E-03	1.02E-02	0.669
G02	IL-1beta	7.40573	4.8025	6.38E-03	1.02E-02	0.648
E03	hGH(Somatotropin)	7.3609	5.0457	7.63E-03	1.20E-02	0.685
C07	Leptin	7.18505	4.8847	9.64E-03	1.49E-02	0.680
F09	PDGF-AB	7.26154	5.0673	1.35E-02	2.06E-02	0.698
A12	Insulin	3.48844	2.1927	1.51E-02	2.27E-02	0.629
E06	hGH(Somatotropin)	4.98068	3.4201	1.60E-02	2.36E-02	0.687
H09	IL-8	4.24917	3.1481	1.78E-02	2.59E-02	0.741
E04	hGH(Somatotropin)	6.66386	4.7771	1.88E-02	2.69E-02	0.717
E02	hGH(Somatotropin)	7.92334	5.5834	1.98E-02	2.79E-02	0.705
E05	hGH(Somatotropin)	4.47901	3.2691	2.44E-02	3.39E-02	0.730
G10	IL-2	6.42624	4.7749	2.57E-02	3.52E-02	0.743
E09	IGF-I	5.38767	3.9334	2.84E-02	3.84E-02	0.730
F10	PDGF-AB	5.49713	4.1117	4.41E-02	5.88E-02	0.748
F11	PDGF-AB	5.78275	4.3455	4.84E-02	6.37E-02	0.751
C08	Leptin	5.17193	3.8095	5.07E-02	6.58E-02	0.737
G11	IL-2	4.60498	3.6252	5.56E-02	7.02E-02	0.787
H10	IL-8	3.90959	3.0546	5.56E-02	7.02E-02	0.781
C09	Leptin	3.90426	3.0166	6.37E-02	7.94E-02	0.773
D09	Exendin-3	5.79135	4.4334	7.59E-02	9.34E-02	0.766
H11	IL-8	3.90641	3.116	8.26E-02	1.00E-01	0.798
E10	IGF-I	5.8049	4.7677	9.77E-02	1.17E-01	0.821
B08	Glucagon	4.98954	3.8937	1.06E-01	1.26E-01	0.780
D11	Exendin-3	4.87217	3.8766	1.10E-01	1.29E-01	0.796
B10	Glucagon	3.84487	3.0562	1.29E-01	1.49E-01	0.795
F12	PDGF-AB	4.7518	3.9564	1.56E-01	1.78E-01	0.833
B12	Glucagon	4.56515	3.8131	1.74E-01	1.94E-01	0.835
E11	IGF-I	4.59369	3.7809	1.74E-01	1.94E-01	0.823
D10	Exendin-3	4.7185	3.9624	1.94E-01	2.14E-01	0.840
B09	Glucagon	5.31598	4.5562	2.46E-01	2.68E-01	0.857
E12	IGF-I	4.18656	3.6171	2.62E-01	2.83E-01	0.864
H12	IL-8	4.84935	4.3404	3.99E-01	4.26E-01	0.895
G12	IL-2	4.69797	4.3014	4.45E-01	4.70E-01	0.916
D12	Exendin-3	5.24673	4.7602	4.94E-01	5.15E-01	0.907
C12	Leptin	4.26935	3.9251	5.06E-01	5.23E-01	0.919
C10	Leptin	5.89552	5.4562	5.45E-01	5.57E-01	0.925
C11	Leptin	5.07094	4.6719	6.41E-01	6.48E-01	0.921
B11	Glucagon	4.21464	4.0209	7.29E-01	7.29E-01	0.954

PMM7_Pre-C-19 Control to LC-19 more than 4 symptoms

Well	Well compound	Mean Pre-C-19 Control	Mean LC-19 >4 Symptoms	Unadjusted p-value	Adjusted p-value	Fold difference in NADH production
A01	NegativeControl	5.14678	2.39883	1.07E-03	7.43E-03	0.466
A02	NegativeControl	5.1383	1.73783	1.60E-04	7.43E-03	0.338
A03	NegativeControl	4.90144	2.06717	4.50E-04	7.43E-03	0.422
A04	NegativeControl	5.06454	2.195	1.17E-03	7.43E-03	0.433
A05	NegativeControl	6.76239	2.8415	1.17E-03	7.43E-03	0.420
B01	Resistin	5.92037	2.98183	1.29E-03	7.43E-03	0.504
B03	Resistin	7.6193	3.85633	1.07E-03	7.43E-03	0.506
B04	Resistin	7.41037	3.8035	8.90E-04	7.43E-03	0.513
B05	Resistin	5.05531	2.64133	1.41E-03	7.43E-03	0.522
C02	Ghrelin	4.79666	2.49183	1.07E-03	7.43E-03	0.519
C04	Ghrelin	6.38197	3.13283	9.70E-04	7.43E-03	0.491
C05	Ghrelin	6.61624	3.33633	1.55E-03	7.43E-03	0.504
D01	Gastrin	6.05677	3.13583	1.55E-03	7.43E-03	0.518
D04	Gastrin	6.4633	3.092	1.55E-03	7.43E-03	0.478
D05	Gastrin	4.85405	2.38133	1.17E-03	7.43E-03	0.491
F04	FGF-1(aFGF)	6.23385	2.98983	9.70E-04	7.43E-03	0.480
F05	FGF-1(aFGF)	7.90533	3.6975	1.55E-03	7.43E-03	0.468
F07	PDGF-AB	4.71016	2.47017	1.55E-03	7.43E-03	0.524
G01	IL-1beta	5.11415	2.628	8.90E-04	7.43E-03	0.514
H04	IL-6	5.62322	2.7845	9.70E-04	7.43E-03	0.495
D06	Gastrin	6.60523	3.34367	1.69E-03	7.75E-03	0.506
A06	NegativeControl	6.09062	2.712	2.42E-03	8.18E-03	0.445
B02	Resistin	4.81089	2.4985	2.64E-03	8.18E-03	0.519
B06	Resistin	6.91427	3.85117	2.42E-03	8.18E-03	0.557
C03	Ghrelin	5.85501	3.03817	2.42E-03	8.18E-03	0.519
D03	Gastrin	6.4365	3.29117	2.64E-03	8.18E-03	0.511
D07	Exendin-3	6.40514	3.33233	2.64E-03	8.18E-03	0.520
F01	FGF-1(aFGF)	5.94069	3.2785	2.03E-03	8.18E-03	0.552
F03	FGF-1(aFGF)	8.33372	4.1625	2.03E-03	8.18E-03	0.499
G04	IL-1beta	7.52447	3.785	2.42E-03	8.18E-03	0.503
G08	IL-2	4.91022	2.55933	2.42E-03	8.18E-03	0.521
C06	Ghrelin	7.38307	3.86317	2.88E-03	8.38E-03	0.523
H03	IL-6	5.56743	3.026	2.88E-03	8.38E-03	0.544
F02	FGF-1(aFGF)	7.84396	4.17667	3.14E-03	8.61E-03	0.532
G05	IL-1beta	7.95125	4.16983	3.14E-03	8.61E-03	0.524
H08	IL-8	4.5062	2.5615	3.42E-03	9.12E-03	0.568
F06	FGF-1(aFGF)	7.81416	4.05033	3.72E-03	9.16E-03	0.518
G03	IL-1beta	7.85544	4.1975	3.72E-03	9.16E-03	0.534
H01	IL-6	5.50475	3.05	3.72E-03	9.16E-03	0.554
A07	Insulin	5.02413	2.27967	4.05E-03	9.72E-03	0.454
D02	Gastrin	5.14458	2.71867	4.40E-03	1.03E-02	0.528
H05	IL-6	5.52751	3.00567	4.78E-03	1.09E-02	0.544
G06	IL-1beta	6.09258	3.3445	5.19E-03	1.13E-02	0.549
H06	IL-6	5.94409	3.30433	5.19E-03	1.13E-02	0.556
C01	Ghrelin	5.92672	3.45967	5.64E-03	1.15E-02	0.584
E07	IGF-I	5.44121	3.104	5.64E-03	1.15E-02	0.570
F08	PDGF-AB	7.30249	3.8955	5.64E-03	1.15E-02	0.533
H02	IL-6	4.64788	2.65267	6.11E-03	1.22E-02	0.571
G07	IL-2	7.24591	4.1205	6.62E-03	1.30E-02	0.569
B07	Glucagon	5.95829	3.37433	7.17E-03	1.35E-02	0.566
E03	hGH(Somatotropin)	7.3609	4.36017	7.17E-03	1.35E-02	0.592
A08	Insulin	4.47747	2.29	9.07E-03	1.68E-02	0.511
H07	IL-8	5.96425	3.6035	9.80E-03	1.78E-02	0.604
G02	IL-1beta	7.40573	4.26033	1.06E-02	1.88E-02	0.575
D08	Exendin-3	5.02829	2.84033	1.14E-02	1.99E-02	0.565
E01	hGH(Somatotropin)	4.74702	2.81917	1.23E-02	2.11E-02	0.594

E02	hGH(Somatotropin)	7.92334	4.72883	1.43E-02	2.40E-02	0.597
E08	IGF-I	6.80794	4.11217	1.54E-02	2.50E-02	0.604
G09	IL-2	5.48638	3.519	1.54E-02	2.50E-02	0.641
E06	hGH(Somatotropin)	4.98068	2.943	1.65E-02	2.64E-02	0.591
E04	hGH(Somatotropin)	6.66386	4.1955	1.77E-02	2.79E-02	0.630
C07	Leptin	7.18505	4.3095	1.91E-02	2.95E-02	0.600
E05	hGH(Somatotropin)	4.47901	2.8575	2.35E-02	3.58E-02	0.638
F09	PDGF-AB	7.26154	4.65133	3.30E-02	4.94E-02	0.641
H09	IL-8	4.24917	2.88233	4.01E-02	5.92E-02	0.678
A09	Insulin	3.23996	2.0055	4.27E-02	6.03E-02	0.619
A10	Insulin	3.11838	2.05167	4.27E-02	6.03E-02	0.658
E09	IGF-I	5.38767	3.55917	4.27E-02	6.03E-02	0.661
A11	Insulin	3.88037	2.54417	5.48E-02	7.62E-02	0.656
C09	Leptin	3.90426	2.7375	5.82E-02	7.87E-02	0.701
G10	IL-2	6.42624	4.4435	5.82E-02	7.87E-02	0.691
C08	Leptin	5.17193	3.42683	6.18E-02	8.13E-02	0.663
F10	PDGF-AB	5.49713	3.7085	6.18E-02	8.13E-02	0.675
A12	Insulin	3.48844	2.20983	7.38E-02	9.20E-02	0.633
B08	Glucagon	4.98954	3.44783	7.38E-02	9.20E-02	0.691
B12	Glucagon	4.56515	3.209	7.38E-02	9.20E-02	0.703
H10	IL-8	3.90959	2.78	7.38E-02	9.20E-02	0.711
D11	Exendin-3	4.87217	3.50833	8.27E-02	1.01E-01	0.720
F11	PDGF-AB	5.78275	4.041	8.27E-02	1.01E-01	0.699
D09	Exendin-3	5.79135	3.95317	8.75E-02	1.05E-01	0.683
G11	IL-2	4.60498	3.33533	9.78E-02	1.16E-01	0.724
F12	PDGF-AB	4.7518	3.5695	1.15E-01	1.35E-01	0.751
B10	Glucagon	3.84487	2.7875	1.21E-01	1.39E-01	0.725
E10	IGF-I	5.8049	4.434	1.21E-01	1.39E-01	0.764
H11	IL-8	3.90641	2.8745	1.28E-01	1.44E-01	0.736
D10	Exendin-3	4.7185	3.56017	1.42E-01	1.58E-01	0.755
B09	Glucagon	5.31598	4.0255	1.49E-01	1.62E-01	0.757
E11	IGF-I	4.59369	3.45767	1.49E-01	1.62E-01	0.753
E12	IGF-I	4.18656	3.35233	2.28E-01	2.46E-01	0.801
C10	Leptin	5.89552	4.88383	2.95E-01	3.15E-01	0.828
D12	Exendin-3	5.24673	4.30533	3.21E-01	3.38E-01	0.821
C11	Leptin	5.07094	4.22267	3.47E-01	3.54E-01	0.833
C12	Leptin	4.26935	3.54817	3.47E-01	3.54E-01	0.831
H12	IL-8	4.84935	3.99683	3.47E-01	3.54E-01	0.824
B11	Glucagon	4.21464	3.64833	4.19E-01	4.19E-01	0.866
G12	IL-2	4.69797	4.01233	4.19E-01	4.19E-01	0.854

PMM7_Pre-C-19 Control to LC-19 Official Diagnosis

Well	Well compound	Mean Pre-C-19 Control	Mean LC-19 official Diagnosis	Unadjusted p-value	Adjusted p-value	Fold difference in NADH production
A01	NegativeControl	5.147	1.778	5.92E-03	5.49E-02	0.345
A02	NegativeControl	5.138	1.523	5.26E-03	5.49E-02	0.296
A03	NegativeControl	4.901	1.457	4.67E-03	5.49E-02	0.297
A04	NegativeControl	5.065	1.439	5.26E-03	5.49E-02	0.284
A05	NegativeControl	6.762	1.711	4.14E-03	5.49E-02	0.253
A06	NegativeControl	6.091	1.594	5.26E-03	5.49E-02	0.262
A07	Insulin	5.024	1.230	4.67E-03	5.49E-02	0.245
A08	Insulin	4.477	1.356	7.47E-03	5.49E-02	0.303
A09	Insulin	3.240	1.216	1.61E-02	5.49E-02	0.375
A10	Insulin	3.118	1.321	1.45E-02	5.49E-02	0.424
A11	Insulin	3.880	1.716	2.20E-02	5.49E-02	0.442
A12	Insulin	3.488	1.078	1.61E-02	5.49E-02	0.309
B01	Resistin	5.920	2.793	1.99E-02	5.49E-02	0.472
B03	Resistin	7.619	3.453	1.61E-02	5.49E-02	0.453
B04	Resistin	7.410	3.379	1.45E-02	5.49E-02	0.456
B05	Resistin	5.055	2.314	1.79E-02	5.49E-02	0.458
B06	Resistin	6.914	3.367	2.69E-02	5.49E-02	0.487
C02	Ghrelin	4.797	2.219	1.61E-02	5.49E-02	0.463
C03	Ghrelin	5.855	2.796	2.69E-02	5.49E-02	0.478
C04	Ghrelin	6.382	2.938	2.43E-02	5.49E-02	0.460
C05	Ghrelin	6.616	3.017	2.43E-02	5.49E-02	0.456
D01	Gastrin	6.057	2.975	2.43E-02	5.49E-02	0.491
D03	Gastrin	6.437	2.897	2.43E-02	5.49E-02	0.450
D04	Gastrin	6.463	2.679	1.45E-02	5.49E-02	0.414
D05	Gastrin	4.854	2.097	1.79E-02	5.49E-02	0.432
D06	Gastrin	6.605	2.978	2.20E-02	5.49E-02	0.451
F01	FGF-1(aFGF)	5.941	3.014	2.43E-02	5.49E-02	0.507
F02	FGF-1(aFGF)	7.844	3.785	2.69E-02	5.49E-02	0.482
F03	FGF-1(aFGF)	8.334	3.517	1.30E-02	5.49E-02	0.422
F04	FGF-1(aFGF)	6.234	2.500	1.17E-02	5.49E-02	0.401
F05	FGF-1(aFGF)	7.905	3.052	1.61E-02	5.49E-02	0.386
F06	FGF-1(aFGF)	7.814	3.501	2.69E-02	5.49E-02	0.448
F07	PDGF-AB	4.710	2.054	1.30E-02	5.49E-02	0.436
G01	IL-1beta	5.114	2.321	1.17E-02	5.49E-02	0.454
G03	IL-1beta	7.855	3.555	1.79E-02	5.49E-02	0.453
G04	IL-1beta	7.524	3.023	1.30E-02	5.49E-02	0.402
G05	IL-1beta	7.951	3.449	1.45E-02	5.49E-02	0.434
G06	IL-1beta	6.093	2.767	1.99E-02	5.49E-02	0.454
G07	IL-2	7.246	3.285	1.99E-02	5.49E-02	0.453
G08	IL-2	4.910	2.211	1.99E-02	5.49E-02	0.450
G09	IL-2	5.486	2.818	2.69E-02	5.49E-02	0.514
H01	IL-6	5.505	2.554	1.61E-02	5.49E-02	0.464
H02	IL-6	4.648	2.244	2.69E-02	5.49E-02	0.483
H03	IL-6	5.567	2.590	1.61E-02	5.49E-02	0.465
H04	IL-6	5.623	2.537	1.61E-02	5.49E-02	0.451
H05	IL-6	5.528	2.587	2.43E-02	5.49E-02	0.468
H08	IL-8	4.506	2.068	1.45E-02	5.49E-02	0.459
B02	Resistin	4.811	2.280	2.97E-02	5.81E-02	0.474
D07	Exendin-3	6.405	2.997	2.97E-02	5.81E-02	0.468
E08	IGF-I	6.808	3.296	3.27E-02	6.03E-02	0.484
H06	IL-6	5.944	2.949	3.27E-02	6.03E-02	0.496
H07	IL-8	5.964	3.082	3.27E-02	6.03E-02	0.517
C06	Ghrelin	7.383	3.432	3.59E-02	6.51E-02	0.465
C01	Ghrelin	5.927	3.201	3.95E-02	7.02E-02	0.540
E07	IGF-I	5.441	2.752	4.33E-02	7.56E-02	0.506
B07	Glucagon	5.958	2.978	4.75E-02	7.72E-02	0.500
D02	Gastrin	5.145	2.561	4.75E-02	7.72E-02	0.498
D08	Exendin-3	5.028	2.328	4.75E-02	7.72E-02	0.463
G02	IL-1beta	7.406	3.759	4.75E-02	7.72E-02	0.508
F08	PDGF-AB	7.302	3.872	5.68E-02	9.08E-02	0.530

C07	Leptin	7.185	3.717	6.75E-02	1.06E-01	0.517
B10	Glucagon	3.845	2.208	7.35E-02	1.12E-01	0.574
E01	hGH(Somatotropin)	4.747	2.594	7.35E-02	1.12E-01	0.547
E03	hGH(Somatotropin)	7.361	4.286	9.41E-02	1.37E-01	0.582
E04	hGH(Somatotropin)	6.664	4.076	9.41E-02	1.37E-01	0.612
G10	IL-2	6.426	3.794	9.41E-02	1.37E-01	0.590
F09	PDGF-AB	7.262	4.024	1.02E-01	1.44E-01	0.554
H09	IL-8	4.249	2.577	1.02E-01	1.44E-01	0.607
C09	Leptin	3.904	2.330	1.10E-01	1.53E-01	0.597
C08	Leptin	5.172	2.944	1.28E-01	1.74E-01	0.569
F10	PDGF-AB	5.497	3.159	1.28E-01	1.74E-01	0.575
E05	hGH(Somatotropin)	4.479	2.862	1.38E-01	1.82E-01	0.639
E09	IGF-I	5.388	3.155	1.38E-01	1.82E-01	0.586
D11	Exendin-3	4.872	3.005	1.49E-01	1.88E-01	0.617
E02	hGH(Somatotropin)	7.923	4.664	1.49E-01	1.88E-01	0.589
H10	IL-8	3.910	2.438	1.49E-01	1.88E-01	0.624
B08	Glucagon	4.990	3.031	1.60E-01	1.97E-01	0.607
F11	PDGF-AB	5.783	3.522	1.60E-01	1.97E-01	0.609
G11	IL-2	4.605	2.912	1.72E-01	2.09E-01	0.632
E06	hGH(Somatotropin)	4.981	3.028	1.84E-01	2.21E-01	0.608
E10	IGF-I	5.805	3.894	1.97E-01	2.31E-01	0.671
H11	IL-8	3.906	2.510	1.97E-01	2.31E-01	0.642
E11	IGF-I	4.594	2.938	2.11E-01	2.41E-01	0.640
E12	IGF-I	4.187	2.818	2.11E-01	2.41E-01	0.673
D09	Exendin-3	5.791	3.590	2.56E-01	2.89E-01	0.620
B09	Glucagon	5.316	3.726	2.73E-01	3.04E-01	0.701
C11	Leptin	5.071	3.727	2.90E-01	3.20E-01	0.735
D10	Exendin-3	4.719	3.282	3.08E-01	3.36E-01	0.695
C10	Leptin	5.896	4.543	3.26E-01	3.48E-01	0.771
H12	IL-8	4.849	3.543	3.26E-01	3.48E-01	0.731
F12	PDGF-AB	4.752	3.312	3.46E-01	3.65E-01	0.697
B12	Glucagon	4.565	3.296	3.66E-01	3.82E-01	0.722
B11	Glucagon	4.215	3.301	3.86E-01	3.95E-01	0.783
C12	Leptin	4.269	3.216	3.86E-01	3.95E-01	0.753
D12	Exendin-3	5.247	3.874	4.08E-01	4.12E-01	0.738
G12	IL-2	4.698	3.721	4.30E-01	4.30E-01	0.792
