# Supplementary material for: Cellular Metabolic Signatures of Long COVID-19
Source: Infect Dis Rep. 2026 May 26;18(3):50. doi: 10.3390/idr18030050 (PMC13299657; doi:10.3390/idr18030050)
Supplement: Supplementary file 1 [file idr-18-00050-s001.zip › Supp. Folder tables.pdf]

Supplementary Table S1 (Table S1): Reagents for COVID-19 diagnostic assay

Reagent	Vendor	Part Number	Description
HS_RPP30 Synthetic DNA	Integrated DNA Technologies	299788131	P1 positive control
Luna Buffer Probe One-Step Reaction	New England Biolabs	M3006B	
Luna WarmStart RT Enzyme Mix	New England Biolabs	M3002B	
nCOV_N1 Probe Aliquot, 50 nmol	Integrated DNA Technologies	10006832	FAM-ACC CCG CAT /ZEN/ TAC GTTTGGTGGACC-3IABkFQ
nCOV_N1 Forward Primer, 100 nmol	Integrated DNA Technologies	10006830	GACCCCAAAATCAGCGAAAT
nCOV_N1 Reverse Primer, 100 nmol	Integrated DNA Technologies	10006831	TCTGGTTACTGCCAGTTGAATCTG
RNAse P (ATTO 647) Probe, 50 nmol	Integrated DNA Technologies	10007062	5Cy5-TTC TGA CCT /ZEN/ GAA GGCTCTGCGCG-3IABkFQ
RNAse P Forward Primer, 100nmol	Integrated DNA Technologies	10006836	AGATTTGGACCTGCGAGCG
RNAse P Reverse Primer, 100 nmol	Integrated DNA Technologies	10006837	GAGCGGCTGTCTCCACAA GT
Sars-CoV-2 Synthetic RNA Control 2	Twist Biosciences	102024 / 103907 / 103909	N1 Positive Control

Supplementary Table S2 (Table S2): Touchdown RT-qPCR protocol. Thermocycling conditions for one-step RT-qPCR SARS-CoV-2 diagnostic assay.

Stage	Temperature (°C)	Duration	Number of Cycles
Reverse Transcription	55	10 min	1
Initial Denaturation	95	1 min	1
Touchdown	95	10 sec	3
	72	30 sec	
	95	10 sec	3
	69	30 sec	
	95	10 sec	3
	66	30 sec	
Main Amplification	95	10 sec	40
	65	30 sec	

Supplementary Table S3 (Table S3): Reagents for EBV and HHV-6 PCR assay

Reagent	Vendor	Part Number	Description
HS_RPP30 Synthetic DNA	Integrated DNA Technologies	299788131	P1 positive control
Luna Buffer Probe One-Step Reaction	New England Biolabs	M3006B	
Luna WarmStart RT Enzyme Mix	New England Biolabs	M3002B	
EBV Probe Aliquot, 50 nmol	Integrated DNA Technologies	Custom	/5SUN/GTTTGGCGT /ZEN/ CTCAGGCTATGAAG-3IABkFQ
EBV Forward Primer, 100 nmol	Integrated DNA Technologies	Custom	GGTAATGATTACGCAGGCC
EBV Reverse Primer, 100 nmol	Integrated DNA Technologies	Custom	CAACAGATTTCCGAACCTTGTC
RNAse P (ATTO 647) Probe, 50 nmol	Integrated DNA Technologies	10007062	5Cy5-TTC TGA CCT /ZEN/ GAA GGCTCTGCGCG-3IABkFQ
RNAse P Forward Primer, 100nmol	Integrated DNA Technologies	10006836	AGATTTGGACCTGCGAGCG
RNAse P Reverse Primer, 100 nmol	Integrated DNA Technologies	10006837	GAGCGGCTGTCTCCACAA GT
Sars-CoV-2 Synthetic RNA Control 2	Twist Biosciences	102024 / 103907 / 103909	N1 Positive Control
HHV6 Probe Aliquot, 50 nmol	Integrated DNA Technologies	Custom	/56-FAM/CG CAA ACG A/ZEN/C AAA GCC A/3IABkFQ/
HHV6 Forward Primer, 100 nmol	Integrated DNA Technologies	Custom	TAA ATA TCG ATG CCG CTC TG
HHV6 Reverse Primer, 100 nmol	Integrated DNA Technologies	Custom	ACG TTC TAG CCA TCT TCT TTG

Supplementary Table S4 (Table S4): Touchdown qPCR protocol. Thermocycling conditions for PCR for EBV and HHV-6

Stage	Temperature (°C)	Duration	Number of Cycles
Reverse Transcription	55	10 min	1
Initial Denaturation	95	1 min	1
Touchdown	95	10 sec	3
	72	30 sec	
	95	10 sec	3
	69	30 sec	
	95	10 sec	3

Supplementary Table S5 (Table S5): PM-M1: Significant ($p < 0.05$) changes in metabolic activity in the utilization of carbon and nitrogen energy sources in PM-M1 by individuals with LC-19 compared to pre-C-19 controls.

Well: Differential Compounds	Difference in NADH between LC-19 and Pre-C-19 controls	Significance (p-values)		Well: Differential Compounds	Difference in NADH between LC-19 and Pre-C-19 controls	Significance (p-values)
**PM-M1:**						
B04:D-(+)-Glucose	↑	1 x 10-5		E09: Thymidine	↑	1.6 x 10-4
B09:b-Methyl-D-Glucoside	↑	1 x 10-5		B03: L-Glucose	↑	4.7 x 10-4
B10:Salicin	↑	1 x 10-5		H09: 3-Hydroxy 2-Butanone	↑	4.7 x 10-4
C01:D-Glucosaminic Acid	↑	1 x 10-5		E01: Melibionic Acid	↑	5.0 x 10-4
D03:L-Rhamnose	↑	1 x 10-5		H04: D,L-a-Hydroxy-ButyricAcid	↑	1.13 x 10-3
D09:D-Raffinose	↑	1 x 10-5		A10: D-Trehalose	↑	1.37 x 10-3
F06:m-Inositol	↑	1 x 10-5		B08: a-Methyl-D-Glucoside	↑	1.62 x 10-3
F10:D,L-a-GlycerolPhosphate	↑	1 x 10-5		G06: a-Keto-GlutaricAcid	↓	1.62 x 10-3
G04:MethylPyruvate	↑	1 x 10-5		E02: D-Melibiose	↑	2.10 x 10-3
G07:Succinamic Acid	↑	1 x 10-5		G01: Tricarballylic Acid	↑	2.36 x 10-3
G12:m-Tartaric Acid	↑	1 x 10-5		A06: Glycogen	↓	2.65 x 10-3
H03:a-Keto-Butyric Acid	↑	1 x 10-5		E08: Sedoheptulosan	↑	2.77 x 10-3
B05:D-(+)-Glucose	↓	2 x 10-5		F08: 1,2-Propanediol	↑	3.32 x 10-3
C05:D-Mannose	↓	2 x 10-5		B11: D-Sorbitol	↑	3.40 × 10?^{3}
E04:a-Methyl-D-Galactoside	↑	2 x 10-5		E11: Adenosine	↑	3.40 × 10?^{3}
E06:n-acetyl-neuraminic acid	↑	2 x 10-5		B07: 3-Methyl Glucose	↑	3.81 × 10?^{3}
F03:D-Arabinose	↑	2 x 10-5		H01: Acetoacetic Acid	↑	3.98 × 10?^{3}
G10:L-MalicAcid	↑	2 x 10-5		G08: SuccinicAcid	↑	9.03 × 10?^{3}
C07:D-Mannitol	↑	4 x 10-5		C04: Mannan	↑	9.41 × 10?^{3}
E12:Inosine	↑	5 x 10-5		D02: L-Sorbose	↓	1.888 × 10?^{2}
G03:MethylD-Lactate	↑	5 x 10-5		D05: D-Fucose	↓	2.075 × 10?^{2}
C12:Turanose	↑	7 x 10-5		H06: g-Hydroxy-ButyricAcid	↑	2.153 × 10?^{2}
B01:D-Glucose-6-Phosphate	↑	1.2 x 10-4		D10: Lactitol	↑	2.234 × 10?^{2}
D12:a-D-Lactose	↑	1.2 x 10-4		D07: D-Fructose	↑	2.318 × 10?^{2}
E07:Pectin	↑	1.2 x 10-4		E10: Uridine	↑	2.405 × 10?^{2}
H12:Hexanoic Acid	↑	1.2 x 10-4		A08: Maltotriose	↓	2.496 × 10?^{2}
A05:Dextrin	↓	1.5 x 10-4		G09: MonoMethylSuccinate	↑	2.590 × 10?^{2}
B12:N-Acetyl-D-Glucosamine	↑	1.5 x 10-4		C10: Sucrose	↑	2.993 × 10?^{2}
F12:Citric Acid	↑	1.5 x 10-4		B02: D-Glucose-1-Phosphate	↑	4.033 × 10?^{2}

Supplementary Table S6 (Table S6): PM-M6: Wells with significant ($p < 0.05$) differences in metabolic activity in response to hormones and modulators in cells from individuals with the LC-19 compared to pre-C-19 controls. The red downward arrow shows reduced NADH production, and the green upward arrow shows increased production.

Well: Differential Compounds	Difference in NADH between LC-19 and Pre-C-19 controls	Significance (p-values)		Well: Differential Compounds	Difference in NADH between LC-19 and Pre-C-19 controls	Significance (p-values)
**PM-M6**						
F04: Dexamethasone	↓	4×10^{-4}		H05: 4,5-a-Dihydrotestosterone	↓	1.6 x 10-4
A07: Dibutyryl-cAMP	↓	4.6×10^{-4}		D02: L-Leucine	↓	4.7 x 10-4
C01: Epinephrine	↓	5.1×10^{-4}		E02: Triiodothyronine	↓	4.7 x 10-4
C06: Epinephrine	↓	5.1×10^{-4}		G02: Progesterone	↓	5.0 x 10-4
D01: L-Leucine	↓	5.1×10^{-4}		D08: Creatine	↓	1.13 x 10-3
E03: Triiodothyronine	↓	5.1×10^{-4}		H01: 4,5-a-Dihydrotestosterone	↓	1.37 x 10-3
E04: Triiodothyronine	↓	5.1×10^{-4}		H02: 4,5-a-Dihydrotestosterone	↓	1.62 x 10-3
F01: Dexamethasone	↓	5.1×10^{-4}		G03: Progesterone	↓	1.62 x 10-3
F05: Dexamethasone	↓	5.1×10^{-4}		E07: Thyroxine	↓	2.10 x 10-3
F06: Dexamethasone	↓	5.1×10^{-4}		C07: Norepinephrine	↓	2.36 x 10-3
G06: Progesterone	↓	5.1×10^{-4}		H06: 4,5-a-Dihydrotestosterone	↓	2.65 x 10-3
C02: Epinephrine	↓	5.6×10^{-4}		B01: 3-Isobutyl-1-Methylxanthine	↓	2.77 x 10-3
C05: Epinephrine	↓	5.6×10^{-4}		F08: Hydrocortisone	↓	3.32 x 10-3
D03: L-Leucine	↓	5.6×10^{-4}		G07: beta-Estradiol	↓	$3.40 \times 10?^{3}$
E06: Triiodothyronine	↓	5.6×10^{-4}		D06: L-Leucine	↓	$3.40 \times 10?^{3}$
F02: Dexamethasone	↓	5.6×10^{-4}		H08: Aldosterone	↓	$3.81 \times 10?^{3}$
F03: Dexamethasone	↓	5.6×10^{-4}		H07: Aldosterone	↓	$3.98 \times 10?^{3}$
G04: Progesterone	↓	6.3×10^{-4}		B02: 3-Isobutyl-1-Methylxanthine	↓	$9.03 \times 10?^{3}$
D07: Creatine	↓	6.9×10^{-4}		A09: Dibutyryl-cAMP	↓	$9.41 \times 10?^{3}$
E05: Triiodothyronine	↓	6.9×10^{-4}		C08: Norepinephrine	↓	$1.888 \times 10?^{2}$
F07: Hydrocortisone	↓	6.9×10^{-4}		B07: Caffeine	↓	$2.075 \times 10?^{2}$
H03: 4,5-a-Dihydrotestosterone	↓	6.9×10^{-4}		E08: Thyroxine	↓	$2.153 \times 10?^{2}$
C03: Epinephrine	↓	8.1×10^{-4}		D09: Creatine	↓	$2.234 \times 10?^{2}$
C04: Epinephrine	↓	8.1×10^{-4}		G08: beta-Estradiol	↓	$2.318 \times 10?^{2}$
A08: Dibutyryl-cAMP	↓	8.8×10^{-4}		B08: Caffeine	↓	$2.405 \times 10?^{2}$
G05: Progesterone	↓	8.8×10^{-4}		C09: Norepinephrine	↓	$2.496 \times 10?^{2}$
G01: Progesterone	↓	1×10^{-3}		D10: Creatine	↓	$2.590 \times 10?^{2}$
H04: 4,5-a-Dihydrotestosterone	↓	1×10^{-3}		B05: 3-Isobutyl-1-Methylxanthine	↑	$2.993 \times 10?^{2}$
D04: L-Leucine	↓	1×10^{-3}		B03: 3-Isobutyl-1-Methylxanthine	↓	$4.033 \times 10?^{2}$
E01: Triiodothyronine	↓	1×10^{-3}		F09: Hydrocortisone	↓	$4.033 \times 10?^{2}$
D02: L-Leucine	↓	1.3×10^{-3}				

Supplementary Table S7 (Table S7): PM-M7: Wells with significant ($p < 0.05$) changes in metabolic activity in response to hormones and modulators in cells from individuals with the LC-19 compared to pre-C-19 controls. The red downward arrow shows reduced NADH production.

Well: Differential Compounds	Difference in NADH between LC-19 and Pre-C-19 controls	Significance (p-values)		Well: Differential Compounds	Difference in NADH between LC-19 and Pre-C-19 controls	Significance (p-values)
**PM-M7**						
G01: IL-1beta	↓	1.1 x 10-3		G03: IL-1beta	↓	2×10^{-3}
A07: Insulin	↓	1.3 x 10-3		G07: IL-2	↓	2×10^{-3}
A08: Insulin	↓	1.3 x 10-3		E07: IGF-I	↓	2.8×10^{-3}
B04: Resistin	↓	1.3 x 10-3		G06: IL-1beta	↓	2.8×10^{-3}
C02: Ghrelin	↓	1.3 x 10-3		B02: Resistin	↓	2.9×10^{-3}
C04: Ghrelin	↓	1.3 x 10-3		D02: Gastrin	↓	4.2×10^{-3}
C05: Ghrelin	↓	1.3 x 10-3		F08: PDGF-AB	↓	4.2×10^{-3}
D04: Gastrin	↓	1.3 x 10-3		H06: IL-6	↓	4.2×10^{-3}
D05: Gastrin	↓	1.3 x 10-3		H02: IL-6	↓	4.4×10^{-3}
D06: Gastrin	↓	1.3 x 10-3		A10: Insulin	↓	4.6×10^{-3}
F01: FGF-1(aFGF)	↓	1.3 x 10-3		D08: Exendin-3	↓	4.6×10^{-3}
F04: FGF-1(aFGF)	↓	1.3 x 10-3		G09: IL-2	↓	4.6×10^{-3}
F05: FGF-1(aFGF)	↓	1.3 x 10-3		H05: IL-6	↓	4.6×10^{-3}
F07: PDGF-AB	↓	1.3 x 10-3		C01: Ghrelin	↓	4.8×10^{-3}
G04: IL-1beta	↓	1.3 x 10-3		B07: Glucagon	↓	54×10^{-3}
G05: IL-1beta	↓	1.3 x 10-3		E08: IGF-I	↓	6×10^{-3}
H04: IL-6	↓	1.3 x 10-3		A09: Insulin	↓	7.9×10^{-3}
B03: Resistin	↓	1.4 x 10-3		H07: IL-8	↓	7.9×10^{-3}
D01: Gastrin	↓	1.4 x 10-3		A11: Insulin	↓	8.2×10^{-3}
B01: Resistin	↓	1.5 x 10-3		E01: hGH(Somat	↓	1×10^{-2}
B05: Resistin	↓	1.5 x 10-3		G02: IL-1beta	↓	1×10^{-2}
C06: Ghrelin	↓	1.5 x 10-3		E03: hGH(Somat	↓	1×10^{-2}
D07: Exendin-3	↓	1.5 x 10-3		C07: Leptin	↓	1.5×10^{-2}
H01: IL-6	↓	1.5 x 10-3		F09: PDGF-AB	↓	2×10^{-2}
G08: IL-2	↓	1.6 x 10-3		A12: Insulin	↓	2.2×10^{-2}
F03: FGF-1(aFGF)	↓	1.7 x 10-3		E06: hGH(Somat	↓	2.3×10^{-2}
H08: IL-8	↓	2 x 10-3		H09: IL-8	↓	2.9×10^{-2}
B06: Resistin	↓	2 x 10-3		E04: hGH(Somat	↓	2.7×10^{-2}
C03: Ghrelin	↓	2 x 10-3		E02: hGH(Somat	↓	2.8×10^{-2}
D03: Gastrin	↓	2 x 10-3		E05: hGH(Somat	↓	3.3×10^{-2}
H03: IL-6	↓	2 x 10-3		G10: IL-2	↓	3.5×10^{-2}
F02: FGF-1(aFGF)	↓	2 x 10-3		E09: IGF-I	↓	3.8×10^{-2}
F06: FGF-1(aFGF)	↓	2 x 10-3				
